# Discovery of MK8383s with Antifungal Activity from Mangrove Endophytic Fungi *Medicopsis* sp. SCSIO 40440 Against Fusarium Wilt of Banana

**DOI:** 10.3390/md23020088

**Published:** 2025-02-18

**Authors:** Tianyu Zhou, Yulei Qiao, Lu Wang, Zifeng Li, Haibo Zhang, Liping Zhang, Shengrong Liao, Minhui Li, Changsheng Zhang, Wenjun Zhang

**Affiliations:** 1 CAS Key Laboratory of Tropical Marine Bio-Resources and Ecology/Guangdong Key Laboratory of Marine Materia Medica, South China Sea Institute of Oceanology, Chinese Academy of Sciences, 164 West Xingang Road, Guangzhou 510301, China; 18321667897@163.com (T.Z.); alei795@126.com (Y.Q.); 17722820628@163.com (L.W.); zhanghb@scsio.ac.cn (H.Z.); zhanglp@scsio.ac.cn (L.Z.); srliao@scsio.ac.cn (S.L.); 2 University of Chinese Academy of Sciences, Beijing 100049, China; 3 College of Plant Protection, South China Agricultural University, Guangzhou 510642, China; anime801lsa@gmail.com

**Keywords:** mangrove endophytic fungi, decalin carboxylic acids, antifungal activities against *Foc* TR4

## Abstract

Fusarium wilt of banana (FWB), caused by *Fusarium oxysporum* f. sp. *cubense* (*Foc*) tropical race 4 (TR4), poses a severe threat to the global banana industry. The screening of endophytic fungi from the mangrove plant led to the identification of *Medicopsis* sp. SCSIO 40440, which exhibited potent antifungal activity against Fusarium. The further fraction of the extract yielded ten compounds, including MK8383 (**1**) and nine new analogues, MK8383s B-J (**2**–**10**). The structures of **1**–**10** were elucidated using extensive spectroscopic data and single-crystal X-ray diffraction analysis. In vitro antifungal assays revealed that **1** showed strongly antifungal activities against Foc TR4, with an EC_50_ of 0.28 μg/mL, surpassing nystatin and hygromycin B (32 and 16 μg/mL, respectively). Pot experiments showed that **1** or spores of SCSIO 40440 could significantly reduce the virulence of *Foc* TR4 on Cavendish banana.

## 1. Introduction

Fusarium wilt of banana (FWB) is widely recognized as the most challenging epidemic affecting bananas, which are considered the fourth most important staple crop globally, following corn, rice, and wheat [1]. The FWB-causing pathogen is *Fusarium oxysporum* f. sp. *cubense* (*Foc*), a member of the *Fusarium oxysporum* species complex (FOSC) [2]. *Foc* can survive in soil for decades and spreads through contaminated plants, soil, tools, and water [3]. Four races of *Foc* have been identified based on the host banana cultivars, with tropical race 4 (TR4) being the most devastating [1,4]. TR4 can infect nearly all banana cultivars and poses a significant threat to global banana trade and food security, potentially exacerbating poverty in developing nations and intensifying world hunger [1,5]. Initially confined to East Asia and parts of Southeast Asia for over two decades, *Foc* TR4 has spread westward since 2010 to five additional countries in Southeast and South Asia (Vietnam, Laos, Myanmar, India, and Pakistan) and transcontinentally to the Middle East (Oman, Jordan, Lebanon, and Israel) and Africa (Mozambique) [3]. In China and Australia, FWB causes an approximately 40% annual yield loss, resulting in economic losses of USD 91 million and USD 138 million, respectively [6]. Projections estimate FWB will affect 17% of global banana-growing areas by 2040, equating to 36 million tons of production loss valued at over USD 1 billion [7].

Mangrove-associated microorganisms are a proven source of bioactive metabolites [8,9,10], particularly endophytic fungi, which are the second-largest ecological group and play a pivotal role in the biosphere’s creation and maintenance [10]. These fungi are a rich source of diversity of bioactive natural products, attracting significant interest from organic chemists and pharmacologists [10]. Recent research has highlighted their potential in producing antifungal compounds. For instance, symmetrical dimer coumarins showed significant antifungal activities against *Fusarium oxysporum*, surpassing the positive control, triadimefon [11]. Additionally, fusaridioic acid, characterized by a β-lactone ring, exhibited antifungal activities against pathogenic fungus *pestalotiopsis theae* [12].

In our efforts to explore bioactive natural products from mangrove endophytic fungi, *Medicopsis* sp. SCSIO 40440 was isolated from the healthy stem of *Pluchea indica* of mangrove [13]. The strain’s fermentation extract exhibited potent antifungal properties against the FOSC. Subsequently, ten decalin carboxylic acids were isolated from the extract of *Medicopsis*, including MK8383 (**1**) and nine new analogues, MK8383s B–J (**2**–**10**). The structures of **1**–**10** were elucidated by NMR spectroscopic data analyses and X-ray single-crystal diffraction. In vitro antifungal assays against *Fusarium* sp. compound **1** was found to display a strong inhibitory effect on the mycelial growth of *Foc* TR4, with an EC_50_ of 0.28 μg/mL, 57 or 114 times more potent than hygromycin B (16 μg/mL) and nystatin (32 μg/mL), respectively. Pot experiments were carried out to further investigate the biocontrol efficiency of compound **1** and *Medicopsis* sp. SCSIO 40440 against *Foc* TR4. The results showed a considerable reduction in the disease index and effective inhibition of root infection. Herein, we report the isolation, structure elucidation, and antifungal bioactivity of compounds **1**–**10**.

## 2. Results

### 2.1. Antifungal Activity and Isolation

**Figure 1:**
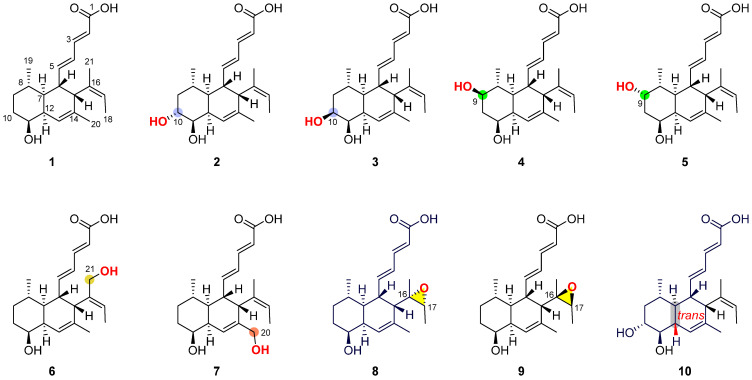
Structures of compounds **1**–**10**. Carbon atom color-coding indicates distinct hydroxylation modifications or a *tran*-decalin ring.

The endophytic fungus *Medicopsis* sp. SCSIO 40440 was isolated from the fresh, healthy stems of *Pluchea indica* and identified through a sequence analysis of the ITS region of its rDNA [13]. The antifungal activity of extracts of *M*. sp. SCSIO 40440 was evaluated, showing strong inhibitory activity against five FOSCs (Table S1). To further characterize the antifungal metabolites, *Medicopsis* sp. SCSIO 40440 was subsequently fermented in oat media. The organic phase was extracted four times using butanone, and the extracts were subjected to a variety of chromatographic steps, including column chromatography, gel chromatography, and semi-preparative HPLC. This process led to the isolation of ten decline carboxylic acids (**1**–**10**), nine of which were new (**2**–**10**) (Figure 1). The structures of isolated compounds were elucidated by HRESIMS, spectroscopic data, and X-ray analysis.

### 2.2. Structure Elucidation

**Figure 2:**
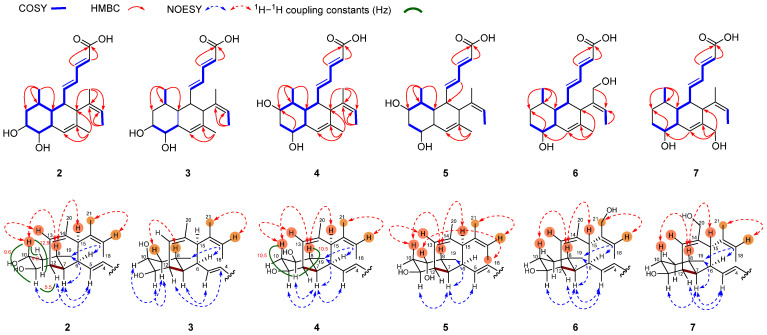
Key COSY, HMBC, and NOESY correlations of **2**–**7**, and ^1^H–^1^H coupling constants (Hz) of **2** and **4**.

**Figure 3:**
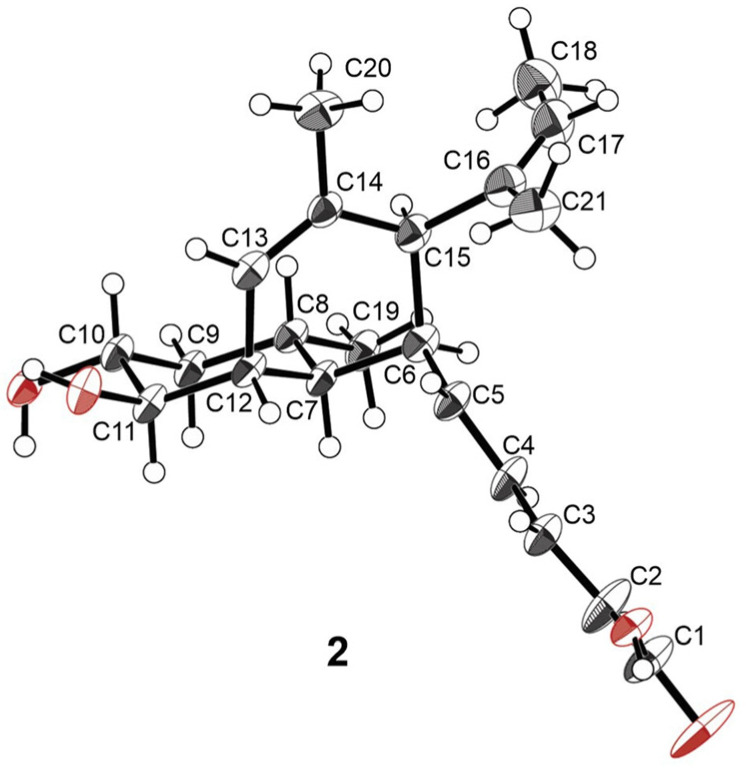
The X-ray crystal structure of **2**. The ellipsoids of non-hydrogen atoms of **2** are shown at 50% probability levels.

Compound **1** was assigned as MK8383 by comparing its HRESIMS and NMR data with those reported in the literature (Table S2, Figure S1) [14,15]. MK8383 B (2) was isolated as a colorless solid. The HR-ESI(+)MS analysis determined a molecular formula of C_21_H_30_O_4_, containing an additional oxygen atom compared to **1**. The ^1^H and ^13^C NMR data in **2** (Table 1 and Table 2, Figure S2) were highly similar to those of **1**. The significant difference was the presence of an additional hydroxyl group (*δ*_H_ 3.42; *δ*_C_ 70.4) at C-10 in **2**, which was supported by the COSY correlations of H-10/H-11, and HMBC correlations from H-8/H-12 to C-10 (Figure 2). The relative configuration of **2** was determined by its ^1^H NMR data and NOESY correlations. The geometries of Δ^2^ and Δ^4^ were determined to be *E* by the corresponding coupling constants of 15.0 Hz and 15.4 Hz. The Δ^16^ was assigned as *Z* due to the observed NOESY correlation of H-17 and H_3_-21. The coupling constants of H-10 (*δ*_H_ 3.42) with H-9_ax_, H-11, and H-9_eq_ were observed to be 12.9 Hz, 9.6 Hz, and 4.2 Hz, respectively (Table 1, Table S3), suggesting an *axial* orientation for H-10, H-9_ax_, and H-11, and an *equatorial* orientation for H-9_eq_. The observed signal at H-11 (*δ*_H_ 3.36) displayed a coupling constant of 5.5 Hz with H-12, implying an *equatorial* orientation for H-12. The NOESY correlations of H-11/H-7, H-7/H_3_-19, H-6/H_3_-19, and H-7/H-5 indicated an *axial* orientation for H-7 and H-11 and an *equatorial* orientation for H_3_-19 and H-6. The NOESY correlations of H-15/H-8 and H-8/H-10 revealed a pseudo-*axial* orientation for H-15. Finally, compound **2** was crystallized in a methanol solution. The single X-ray crystal diffraction analysis using Cu K*α* radiation (CCDC no. 2400777, flack parameter value 0.1 (2)) (Figure 3, Table S4) confirmed the planar structure and the relative configuration of **2**. Given that **2** was proposed to be biosynthetically derived from **1** by hydroxylation at C-11 [16], the absolute configurations of **2** was assigned as 6*S*, 7*S*, 8*S*, 10*R*, 11*R*, 12*S*, and 15*R*, consistent with that of **1**.

10-*epi*-MK8383 B (**3**) was isolated as a white amorphous powder. The HRESIMS analysis of **3** established a molecular formula of C_21_H_30_O_4_, which is an isomer of **2**. The ^1^H and ^13^C NMR data for **3** (Table 1 and Table 2, Figure S3) were almost identical to those of **2**. An extensive NMR analysis suggested that **3** and **2** should have the same planar structure. The NOESY correlations of H-10/H-9_ax_, H-9_ax_/H-11, and H-9_aq_/H-8 revealed the *β*-configuration of 10-OH, which was different from that in **2** (Figure 2, Table S3). Thus, the absolute configurations of **3** was assigned as 6*S*, 7*S*, 8*S*, 10*S*, 11*R*, 12*S*, and 15*R*.

MK8383 C (**4**) was isolated as a white powder. The HRESIMS analysis of **4** demonstrated a molecular formula of C_21_H_30_O_4_, which was the same as that of **2**. The ^1^H and ^13^C NMR data of **4** (Table 1 and Table 2, Figure S4) were similar to those of **2**. The HMBC correlations from H_3_-19 to C-9 supported an OH group at C-9 in **4**, different from the 10-OH in **2**. The ^1^H NMR data of **4** revealed the 9-OH was in an *equatorial* orientation, supported by the large coupling constants of H-9 with H-10_ax_ (*J* = 10.5 Hz) and H-8 (*J* = 10.5 Hz) (Table S5). Furthermore, the relative configuration of **4** were assigned by NOESY correlations (Figure 2), and its absolute configuration was thus assigned as 6*S*, 7*R*, 8*R*, 9*R*, 11*S*, 12*S*, and 15*R*.

9-*epi*-MK8383 C (**5**) was isolated as a white amorphous powder. The HRESIMS analysis determined a molecular formula of C_21_H_30_O_4_ for **5**, which indicated **5** to be an isomer of **4**. The ^1^H and ^13^C NMR data for **5** (Table 1 and Table 2, Figure S5) were almost identical to those of **4**. An extensive NMR analysis suggested that **5** had the same planar structure as that of **4**. The NOESY correlations of H-9/H_3_-18 revealed the *α*-orientation of 9-OH in **5**, different from the *β*-orientation in **4** (Figure 2). Thus, the absolute configurations of **5** was assigned as 6*S*, 7*R*, 8*R*, 9*S*, 11*S*, 12*S*, and 15*R*.

MK8383 D (**6**) was isolated as a white powder. The HRESIMS analysis established a molecular formula of C_21_H_31_O_4_. The ^1^H and ^13^C NMR data of **6** (Table 1 and Table 2, Figure S6) and **1** were highly similar. The difference was that **6** has an additional hydroxyl group at C-21, which was assigned by the HMBC correlations from H_2_-21/H_3_-18 to C-17 (Figure 2). Finally, the relative configuration of **6** was assigned by coupling constants and NOESY correlations, and the absolute configuration assigned as 6*S*, 7*S*, 8*S*, 11*S*, 12*S*, and 15*S*, which was the same as **1** according to the biosynthetic pathway [16].

MK8383 E (**7**) was isolated as a white powder. The HRESIMS analysis established a molecular formula of C_21_H_30_O_4_, which included one oxygen atom more than **1**. The ^1^H and ^13^C NMR data of **7** (Table 1 and Table 2, Figure S7) was similar to those of **1**. The difference was the presence of an additional hydroxyl group at C-20, as evidenced by HMBC correlations from H_2_-20 to C-13/C-14/C-15 (Figure 2). The relative configuration of **7** was determined by coupling constants and NOESY correlations, with the absolute configuration assigned as the same as that of **1**.

**Figure 4:**
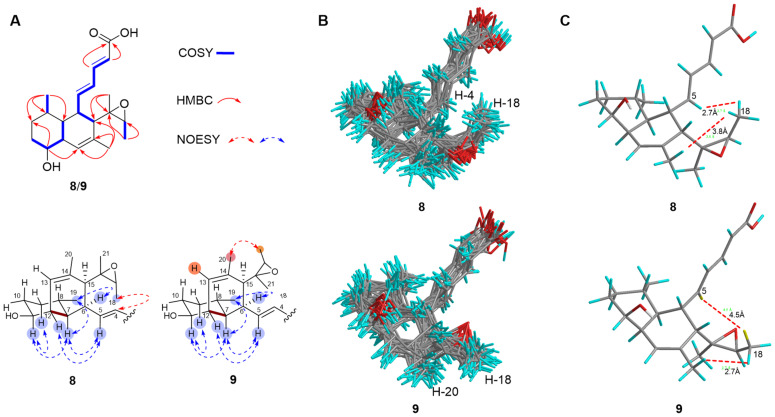
(**A**) Key COSY, HMBC, and NOESY correlations of **8** and **9**. (**B**) MMFF94x energy-minimized 3D structures of **8** (16S*, 17R*) and **9** (16R*, 17R*). (**C**) The distances between H_3_-18/H-4 and H_3_-18/H_3_-19 (The shown distances are the Boltzmann-weighted averages of all conformers within 5 kcal/mol of the global minimum energy geometry).

MK8383 F (**8**) was isolated as a white powder. The HRESIMS analysis established a molecular formula of C_21_H_30_O_4_, having one more oxygen atom than **1**. The NMR data of **8** and **1** (Table 1 and Table 2, Figure S8) were highly similar. The difference was the observation of two sp^3^ oxygen-bearing carbons (*δ*_H_ 2.82/*δ*_C_ 59.4, H-17/C-17; *δ*_C_ 62.7, C-16) in **8**, instead of the sp^2^ methine (*δ*_H_ 5.36/*δ*_C_ 122.5, H-17/C-17) and olefinic quaternary carbon (*δ*_C_ 135.6, C-16) in **1**. This indicated the presence of an epoxide group at C-16/C-17 in **8**, instead of the double bond in **1**, which was further supported by HMBC correlations from H_3_-18/H_3_-21 to C-16/C-17 (Figure 4).

MK8383 G (**9**) was isolated as a white powder. The molecular formula of **9** was established as C_21_H_30_O_4_ by HRESIMS. A detailed NMR data (Table 1 and Table 2, Figure S9) analysis established that the planar structures of **9** and **8** were identical. The stereochemistry of 6*S*, 7*S*, 8*S*, 11*S*, 12*S*, and 15*S* in **8** and **9** were assigned according to their shared biosynthetic pathway, which was the same as that of **1** [16]. Due to the considerable conformational flexibility of the epoxide group, assigning the stereochemistry of C-16/C-17 proved challenging. The careful analysis of the NOESY correlations of **8** and **9** showed a correlation between H_3_-18 and H-4 in **8**, and a correlation between H_3_-18 and H_3_-20 in **9** (Figure 4). Subsequently, molecular models of the *α*-oriented and *β*-oriented epoxide at C-16/C-17 were subjected to a Low-Mode MD conformational search (MMFF94x force field) to identify conformers within 5 kcal/mol of the energy geometry (Figure 4). The Boltzmann-weighted average distance between H_3_-18 and H-4 was found to be shorter in an *α*-oriented epoxide, while the distance between H_3_-18 and H_3_-20 was shorter in a *β*-oriented epoxide (Figure 4). Based on these data, the relative configurations were tentatively assigned as 16S*, 17R* for **8**, and 16R*, 17S* for **9**.

**Figure 5:**
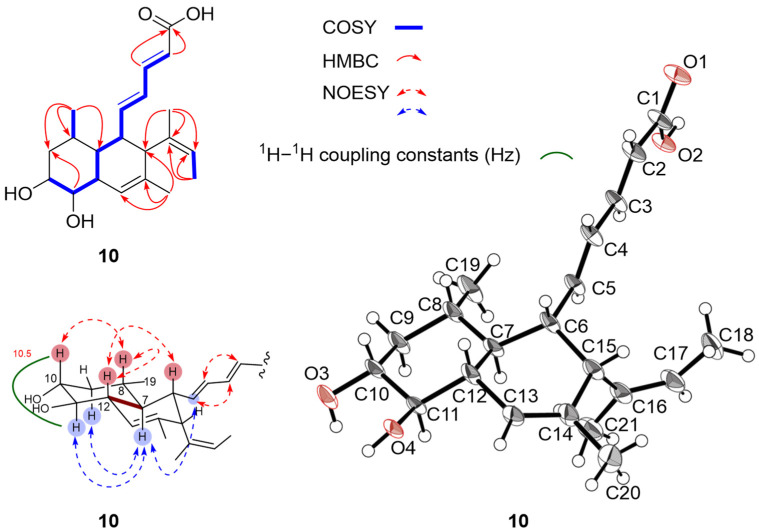
Key COSY, HMBC, and NOESY correlations, ^1^H–^1^H coupling constants (Hz) and X-ray crystal structure of **10**. The ellipsoids of non-hydrogen atoms of **10** are shown at 50% probability.

MK8383 I (**10**) was isolated as a white powder. The HRESIMS analysis established a molecular formula of C_21_H_30_O_4_. Detailed NMR data (Table 1 and Table 2, Figure 5 and Figure S10) suggested that **10** and **2** shared the same planar structure. The large couplings for H-2/H-3 (*J* = 15.3 Hz), as well as the NOESY correlation for H-2/H4 and H-3/H-5, confirmed the *E*-geometry for ^2^Δ and ^4^Δ in **10**. The large couplings for H-10/H-11 (*J* = 10.5 Hz) indicated that both 10-OH and 11-OH were in an *equatorial* orientation, the same as those in **2**. The NOESY correlations of H-10/H-8, H-12/H-8, and H-12/H-6 suggested that H-10/H-8/H-12/H-6 were on the same side of the decalin ring. In contrast, the NOESY correlations of H-11/H-7 and H-7/H-5 indicated that H-11/H-7/H-5 were on the opposite side. This assignment revealed a *trans* configuration between H-7 and H-12 in **10**, instead of a *cis* configuration in **2**. Finally, compound **10** was crystallized from a mixture of MeOH and H_2_O (9:1), which allowed the confirmation of the planar structure of **10** and the unequivocal assignment of its absolute configuration as 6*S*,7*S*,8*S*,10*R*,11*R*,12*R*, and 15*R* by single X-ray crystal diffraction analysis with the Flack parameter of 0.06(11) using Cu K*α* radiation (CCDC no. 2400778) (Table S4).

**Figure 6:**
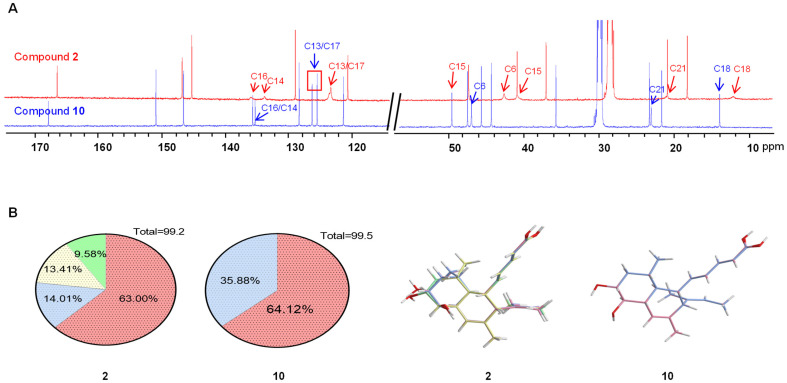
(**A**) ^13^C NMR spectra (175 MHz) of compounds **2** and **10** were recorded under identical conditions using the same instrument. (**B**) The Boltzmann populations of compounds **2** and **10** were determined by DFT calculations at the B3LYP/6-31+G(d,p) level. A pie chart representing the statistical distribution of all conformations was constructed, highlighting those with a population greater than 9%. The colors in the pie chart (left) correspond to the colors assigned to each conformation (right) in the charts.

Compounds **1**–**9** feature a rare *cis*-decalin ring, while **10** has a *trans*-decalin ring. The NMR spectra of **1**–**9** were significantly broadened or even diminished to be unobservable in various solvents (Figure S1B and Figure 6A), whereas **10** displayed sharp resonance signals (Figure 6A). Notable, **2** differs from **10** only in the configuration of C-12. We hypothesized that the broadening NMR signals for **1**–**9** are due to the increased flexibility of the *cis*-decalin ring, as described recently [17,18]. Thus, we moved to DFT calculations to substantiate our hypothesis. We computed the Boltzmann populations of **2** and **10** using DFT calculation at the B3LYP/6- 31+G(d,p) level. The results showed that the conformational space of **2** is occupied by four conformations (99.2%), while **10** exhibits less flexibility, with its conformational space primarily populated by two conformations (99.5%) (Figure 6B). This calculation explains why the ^1^H and ^13^C NMR spectra of **1**–**9** were significantly broadened or even diminished to be unobservable, whereas **10** displayed sharp resonance signals.

### 2.3. Activity of Isolated Compounds Against Fusarium Species

**Figure 7:**
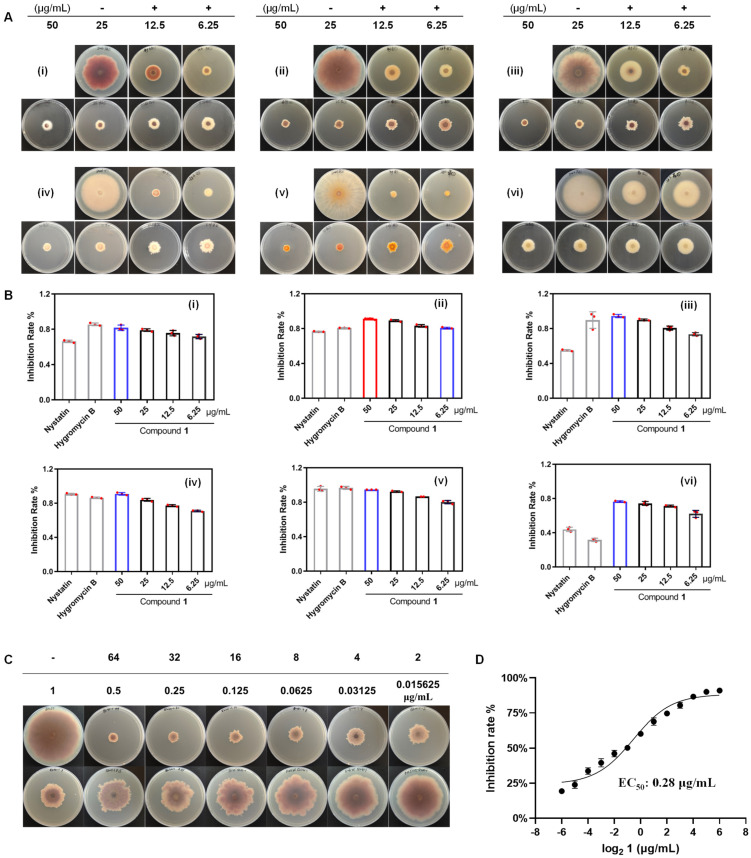
Effect of compound **1** at 6.25, 12.5, 25, and 50 ppm (μg/mL) on mycelial growth of FOSC. (**A**) Colony morphology of FOSC on nystatin and hygromycin B and **1**; (**B**) inhibition rate of **1** on mycelial growth of FOSC; (**C**) colony morphology of *Foc* TR4 on nystatin and hygromycin B and **1**; (**D**) EC_50_ of **1** on mycelial growth of *Foc* TR4. (i. *Fusarium oxysporum* f. sp. *Vasinfectum* (*Fov*); ii. *Fusarium oxysporum* f. sp. *Cubense* (*Foc*) race 4; iii. *Fusarium oxysporum* f. sp. *vasinfectum* (*Fov*) Atk. Sny & Hans; iv. *Fusarium oxysporum* f. sp. *momdicae* (*Fom*) Sun & Huang; v. *Fusarium. oxysporum* f. sp. *Cucumerinu* (*Foc*) Owen; vi. *Fusarium solani* f. sp. *pisi* (*Fsp*); -: DMSO; +: nystatin, hygromycin B).

Compounds **1**, **2**, **4**, **6**, and **10** were evaluated for their antifungal activities using the Kirby–Bauer disc diffusion method. Among these, compounds **1** and **2** demonstrated a range of antifungal effects against various the FOSC (Figure S11). Notably, compound **1** displayed strong antifungal activity against multiple FOSCs, including *Fov* [19], *Foc* TR4 [1], *Fov* Atk. Sny & Hans [20], *Fom* Sun & Huang [21], *Foc* Owen [22], and *Fsp* [23] (Figure S11). Furthermore, the inhibitory effect of compound **1** on the mycelial growth of six different FOSCs was assessed. As shown in Figure 7, at a concentration of 50 ppm (50 μg/mL), the inhibition rate of compound **1** against *Fom* Sun & Huang and *Foc* Owen was comparable to nystatin and hygromycin B (Figure 7A(iv,v),B(iv,v)). Compound **1** showed comparable inhibition activity to hygromycin B and a stronger inhibition effect than that of nystatin against *Fov* and *Fov* Atk. Sny & Hans (Figure 7A(i,iii),B(i,iii)). Significantly, at the same concentration (50 ppm), compound **1** exhibited superior inhibitory effects against *Foc* TR4 and *Fsp* compared to nystatin and hygromycin B (Figure 7A(ii,vi),B(ii,vi)). Notably, even at a lower concentration of 6.75 ppm, compounds **1** demonstrated an inhibition rate of 80.6% against *Foc* TR4, similar to that of hygromycin B (80.0%) and surpassing nystatin (76.5%) at 50 ppm (Figure 7A(ii),B(ii)). Given the potence of compound **1** against *Foc* TR4, we depicted the relationship between the dosage of **1** and the corresponding reduction in mycelial growth (Figure 7C). This graph demonstrated an inverse proportionality between the inhibition rate and **1**. As shown in this figure, the inhibition of mycelial growth decreases at a lower concentration of compound **1**. Based on these data, the EC_50_ (concentration of the compound to inhibit 50% of the mycelial growth) was calculated and presented in Figure 7D. It is noteworthy that pathogen inhibition almost reached 100% at doses of 64 ppm. According to the scale proposed by Edgington et al. in 1971 [24], the efficacy of a compound in reducing a variable associated with the studied pathogen is delineated by the estimated value of its EC_50_. Specifically, when EC_50_ < 1 μg/mL, the compound is classified as having high efficiency (HE). In our investigation, **1** exhibited high efficiency in reducing the mycelial growth of *Foc* TR4, with EC_50_ values of 0.28 μg/mL, while the EC_50_ values for nystatin and hygromycin B are 32 μg/mL and 16 μg/mL (Figure S12).

**Figure 8:**
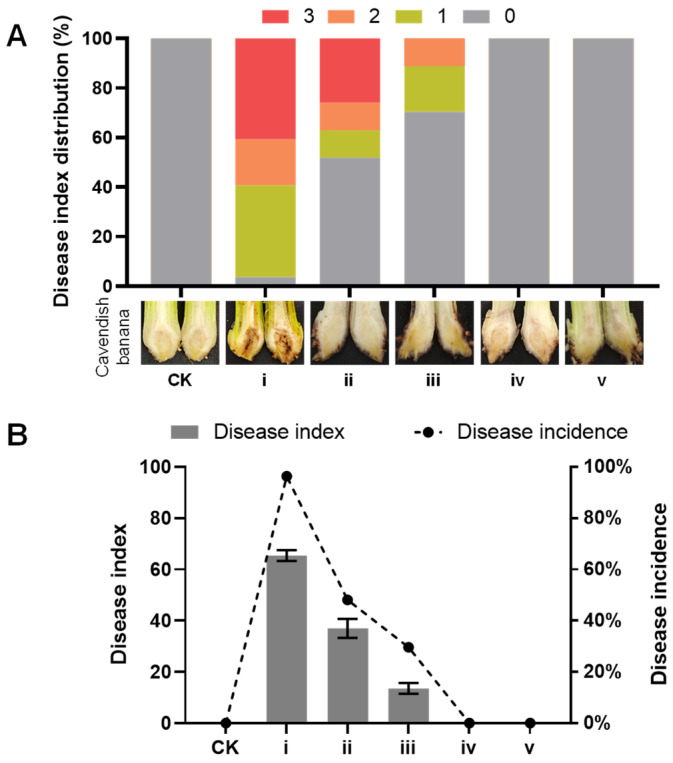
(**A**) Disease symptom on rhizomes of banana tissue culture plantlets; (**B**) disease index and disease incidence on rhizomes of banana tissue culture plantlets. CK: water; i: treated with spores of *Foc* race 4; ii: treated with spores of *Foc* race 4 and *M*. sp. SCSIO 40440; iii: treated with spores of *Foc* race 4 and compound **1**; iv: treated with spores of *M*. sp. SCSIO 40440; v: treated with compound **1**. The data recorded at 30 days after inoculation with compound **1** and spores of SCSIO 40440. The inoculation data of 27 plantlets were randomly divided into three groups for statistical analysis and the mean ± S.D. (*n* = 3).

Subsequently, the disease symptom on rhizomes of banana tissue plantlets were recorded at 30 days after inoculation with **1** and spores of the strain SCSIO 40440. The results showed that **1** and spores of *M.* sp. SCSIO 40440 appeared to be safe to the banana plantlets, since they did not induce obvious vascular discoloration in the corm of the banana plantlets (Figure 8A(iv,v)). In contrast, the pathogen fungus *Foc* TR4 typically causes pronounced vascular discoloration (Figure 8A(i)). When a co-inoculation with **1** or spores of SCSIO 40440 is administered, the virulence of *Foc* TR4 on Cavendish bananas was significantly reduced compared to treatments with *Foc* TR4 alone (Figure 8A(ii,iii)). The disease indexes were 0.14 for the inoculation with compound **1** and 0.37 for the inoculation with spores of SCSIO 40440, respectively, both of which were notably lower than those for the control group (0.65) (Figure 8B).

## 3. Materials and Methods

### 3.1. General Experimental Procedures

Optical rotations were measured with an MCP 500 polarimeter (Anton, Graz, Austria). UV spectra were recorded on a UV-2600 spectrophotometer (Shimadzu, Kyoto, Japan). IR spectra were measured on an IR Affinity-1 FT-IR spectrometer (Shimadzu, Kyoto, Japan). ^1^H, ^13^C, and 2D NMR spectra were recorded on a Bruker AVANCE III HD 500 MHz or 700 MHz NMR spectrometer (Bruker Company, Karlsruhe, Germany) with tetramethylsilane (TMS) as an internal standard. Deuterated NMR solvents were purchased from Cambridge Isotopes. High-Resolution Electrospray Ionization Mass Spectrometry (HRESIMS) data were measured on a Bruker Maxis 4G UHR-TOFMS spectrometer (Bruker Company, Karlsruhe, Germany). Materials for column chromatography (CC) were silica gel (100–200 mesh; Jiangyou Silica Gel Development, Inc, Qingdao, China), Sephadex LH-20 (40–70 μm; Amersham Pharmacia Biotech AB, Uppsala, Sweden), and YMC*GEL ODS-A-HG (12 nm S-50 μm; YMC Company Ltd. Kyoto, Japan). Thin-layer chromatography (TLC, 0.1–0.2 or 0.3–0.4 mm) was conducted with precoated glass plates (silica gel GF254, 10–40 nm, Jiangyou Silica Gel Development, Inc., Qingdao, China). Medium-pressure liquid chromatography (MPLC) was performed with automatic flash chromatography (Cheetahtmmp 200, Bonna-Agela Technologies Co., Ltd., Tianjin, China) with a monitoring wavelength of 254 nm and a collecting wavelength of 304 nm. Semipreparative HPLC was performed on a Hitachi-L2130 HPLC workstation with Hitachi L-2455 detector using a reversed-phase column (Luna C_18_, 250 mm × 10.0 mm, 5 μm; Phenomenex, CA, USA); flow rate 2.5 mL/min. General HPLC analysis was carried out on a reversed-phase column (Luna C_18_, 150 mm × 4.6 mm, 5 μm; Phenomenex, CA, USA) or a polar column (Polar BiPFP 250 × 4.6 mm; 5 μm, Comixsep^®^, P/N FMG-BPF5-EONU) with UV detection at 304 nm on a Agilent series 1200 workstation under the following program: solvent A, 10% CH_3_CN in H_2_O supplementing with 0.1% formic acid; solvent B, 90% CH_3_CN in H_2_O; 5% B to 80% B (0–20 min), 80% B to 100% B (20–21 min), 100% B (21–24 min), 100% B to 5% B (24–25 min), 5% B (25–30 min); flow rate 1 mL/min.

### 3.2. Fungal Material

The strain SCSIO 40440 was isolated from a fresh leaf of the mangrove plant *Pluchea indica* (L.) Less. (E 109.7595°, N 21.5676°) in Zhanjiang Mangrove National Nature Reserve (E 109.7595°, N 21.5676° Zhanjiang, China; With permission). It was incubated on a PDA medium plate (Potato dextrose broth 24.0 g/L, artificial sea salt 15.0 g/L, agar 20.0 g/L, pH 7.0–7.4) at 28 °C for 7 days and then was preserved in the type culture collection of the Center for Marine Microbiology of the South China Sea Institute of Oceanology, Chinese Academy of Sciences. The strain was identified as a species of *Medicopsis* sp. by the ITS gene sequence analysis (GenBank accession number MH865072.1) [13].

### 3.3. Fermentation, Extraction and Isolation

The strain SCSIO 40440 was cultivated on a PDA medium (Potato dextrose broth 24.0 g/L, artificial sea salt 15.0 g/L, agar 20.0 g/L, pH 7.0–7.4) plate at 28 °C for 7 days and then inoculated into 50 mL of PDB medium (Potato dextrose broth 24.0 g/L, artificial sea salt 15.0 g/L, pH 7.0) for incubation at 28 °C and 200 rpm for 3 days in a 250 mL Erlenmeyer flask. After growing to the logarithmic growth phase, a 20 mL portion of the seed cultures was transferred to 250 mL fermenters containing 100 mL of oat medium (oats 500.0 g/L, artificial sea salt 15.0 g/L, pH 7.0). The fermentation (a total of 20 kg) was incubated statically at 28 °C for 35 days. The mycelia were extracted 5 times with 20 L of acetone, and the acetone fractions were concentrated under vacuum to afford the aqueous residues, which were combined with the supernatants and further extracted 3 times with 5 L of butanone. The butanone extracts were concentrated under vacuum to afford the crude extracts (100 g). The extracts were subjected to a normal phase silica gel (200–300 mesh) column and eluted with CHCl_3_/MeOH (90/10, 80/20, 70/30, 50/50, 0/100, *v*/*v*) to obtain four fractions (Fr.1 to Fr.4). Fr.3 was separated by MPLC using a reversed-phase C-18 column to obtain **1** (298.6 mg). Fr.4 was subjected to a normal phase silica gel (200–300 mesh) column and eluted with petroleum ether/ethyl acetate (100/0, 90/10, 80/20, 50/50, 0/100, *v*/*v*) to obtain four fractions (Fr.4-A to Fr.4-G). Subfraction Fr.4-D was purified on a Sephadex LH-20 column, eluted with CHCl_3_/MeOH (1/1, *v*/*v*) to yield six fractions (Fr.4-D-L1 to Fr.4-D-L6). Then, Fr.4-D-L4 was purified by semipreparative HPLC (H_2_O/CH_3_CN, 45/55, *v*/*v*) to obtain **2** (15.4 mg), **4** (17.2 mg), **6** (41.0 mg), and **10** (7.2 mg). Subfraction Fr.4-C was purified on a Sephadex LH-20 column, eluted with CHCl_3_/MeOH (1/1, *v*/*v*) to yield six fractions (Fr.4-C-L1 to Fr.4-C-L6). Then, Fr.4-C-L4 was purified by semipreparative HPLC (H_2_O/CH_3_CN, 45/55, *v*/*v*) to obtain **3** (5.2 mg). Subfraction Fr.4-F was purified on a Sephadex LH-20 column, eluted with CHCl_3_/MeOH (1/1, *v*/*v*) to yield five fractions (Fr.4-F-L1 to Fr.4-F-L5). Then, Fr.4-F-L3 was purified by semipreparative HPLC (H_2_O/CH_3_CN, 45/55, *v*/*v*) to obtain **8** (6.1 mg). Subfraction Fr.4-G was purified on a Sephadex LH-20 column, eluted with CHCl_3_/MeOH (1/1, *v*/*v*) to yield six fractions (Fr.4-G-L1 to Fr.4-G-L6). Then, Fr.4-G-L5 was purified by semipreparative HPLC (H_2_O/CH_3_CN, 45/55, *v*/*v*) to obtain **5** (6.1 mg), **7** (12.1 mg), and **9** (5.8 mg).

*MK8383* (**1**): colorless solid; [*α*${]}_{D}^{25}$+82.8 (*c* 0.1, MeOH); UV (MeOH) λmax (log *ε*) 263 (4.28) nm; IR (film) *ν*_max_ 3368, 2922, 1684, 1636, 1267, 1005 cm^−1^; ^1^H and ^13^C NMR data, see Table 1; HRESIMS *m*/*z* 331.2281 [M + H]^+^; 348.2545 [M + NH_4_]^+^ (calcd for C_21_H_31_O_3_, 331.2268; C_21_H_34_NO_3_, 348.3533).

*MK8383 B* (**2**): colorless solid; [*α*${]}_{D}^{25}$+98.3 (*c* 0.1, MeOH); UV (MeOH) λmax (log *ε*) 262 (4.27) nm; IR (film) *ν*_max_ 3358, 2920, 2359, 1684, 1636, 1024 cm^−1^; ^1^H and ^13^C NMR data, see Table 1; HRESIMS *m*/*z* 347.2220 [M + H]^+^; 364.2474 [M + NH_4_]^+^ (calcd for C_21_H_31_O_4_, 347.2217; C_21_H_34_NO_4_, 364.2482).

*10-epi-MK8383 B* (**3**): colorless solid; [*α*${]}_{D}^{25}$+67.2 (*c* 0.1, MeOH); UV (MeOH) λmax (log *ε*) 263 (4.56) nm; IR (film) *ν*_max_ 3391, 2928, 2359, 1684, 1636, 1003 cm^−1^; ^1^H and ^13^C NMR data, see Table 1; HRESIMS *m*/*z* 347.2218 [M + H]^+^, 364.2491 [M + NH_4_]^+^ (calcd for C_21_H_31_O_4_, 347.2217; C_21_H_34_NO_4_, 364.2482).

*MK8383 C* (**4**): colorless solid; [*α*${]}_{D}^{25}$+57.3 (*c* 0.1, MeOH); UV (MeOH) λmax (log *ε*) 262 (4.30) nm; IR (film) *ν*_max_ 3356, 2920, 2359, 1636, 1013, 689 cm^−1^; ^1^H and ^13^C NMR data, see Table 1; HRESIMS *m*/*z* 347.2221 [M + H]^+^, 364.2484 [M + NH_4_]^+^ (calcd for C_21_H_31_O_4_, 347.2217; C_21_H_34_NO_4_, 364.2482).

*9-epi-MK8383 C* (**5**): colorless solid; [*α*${]}_{D}^{25}$-27.3 (*c* 0.1, MeOH); UV (MeOH) λmax (log *ε*) 262 (4.30) nm; IR (film) *ν*_max_ 3360, 2359, 1653, 1015, 659 cm^−1^; ^1^H and ^13^C NMR data, see Table 1; HRESIMS *m*/*z* 345.2073 [M − H]^-^ (calcd for C_21_H_29_O_4_, 345.2071).

*MK8383 D* (**6**): colorless solid; [*α*${]}_{D}^{25}$+29.4 (*c* 0.1, MeOH); UV (MeOH) λmax (log *ε*) 262 (4.32) nm; IR (film) *ν*_max_ 3358, 2945, 2359, 1668, 1456, 1020 cm^−1^; ^1^H and ^13^C NMR data, see Table 1; HRESIMS *m*/*z* 364.2481 [M + NH_4_]^+^ (calcd for C_21_H_34_NO_4_, 364.2482).

*MK8383 E* (**7**): colorless solid; [*α*${]}_{D}^{25}$+7.2 (*c* 0.1, MeOH); UV (MeOH) λmax (log *ε*) 263 (4.56) nm; IR (film) *ν*_max_ 3359, 2938, 2359, 1658, 1471, 1020 cm^−1^; ^1^H and ^13^C NMR data, see Table 1; HRESIMS *m*/*z* 345.2074 [M − H]^-^ (calcd for C_21_H_29_O_4_, 345.2071).

*MK8383 F* (**8**): colorless solid; [*α*${]}_{D}^{25}$+29.4 (*c* 0.1, MeOH); UV (MeOH) λmax (log *ε*) 259 (4.16) nm; IR (film) *ν*_max_ 3379, 29223, 2361, 1684, 1009, 669 cm^−1^; ^1^H and ^13^C NMR data, see Table 1; HRESIMS *m*/*z* 347.2225 [M + H]^+^, 364.2488 [M + NH_4_]^+^ (calcd for C_21_H_31_O_4_, 347.2217; C_21_H_34_NO_4_, 364.2482).

*MK8383 G* (**9**): colorless solid; [*α*${]}_{D}^{25}$+17.3 (*c* 0.1, MeOH); UV (MeOH) λmax (log *ε*) 259 (4.29) nm; IR (film) *ν*_max_ 3335, 2943, 2362, 1684, 1020, 667 cm^−1^; ^1^H and ^13^C NMR data, see Table 1; HRESIMS *m*/*z* 347.2222 [M + H]^+^, 347.2222 [M + NH_4_]^+^ (calcd for C_21_H_31_O_4_, 347.2217; C_21_H_34_NO_4_, 364.2482).

*MK8383 H* (**10**): colorless solid; [*α*${]}_{D}^{25}$+15.3 (*c* 0.1, MeOH); UV (MeOH) λmax (log *ε*) 263 (4.04) nm; IR (film) *ν*_max_ 3362, 2920, 2359, 1636, 1015, 669 cm^−1^; ^1^H and ^13^C NMR data, see Table 1; HRESIMS *m*/*z* 347.2225 [M + H]^+^, 364.2486 [M + NH_4_]^+^ (calcd for C_21_H_31_O_4_, 347.2217; C_21_H_34_NO_4_, 364.2482).

### 3.4. X-Ray Crystallographic Analysis

Single crystals of **2** and **10** were obtained in methanol or methanol–H_2_O (*v*/*v* 10:1). The suitable crystals were selected, and the crystal data were collected on an XtaLAB AFC12 (RINC): Kappa single diffractometer with Cu K*α* radiation (*λ* = 1.54184 Å). The crystal was kept at 100.0(3) K during the data collection. Using Olex2 [25], the crystal structure was solved with the SHELXT 2018/2 [26] structure solution program with Intrinsic Phasing and refined with the SHELXL refinement package using Least Squares minimization. Crystallographic data have been deposited in the Cambridge Crystallographic Data Center with the deposition number CCDC 2400777 for **2** and 2400778 for **10**.

Crystal Data for **2** C_84_H_118_O_16_ (M =1383.78 g/mol): monoclinic, space group *P*2_1_ (no. 4), *a* = 9.1600(5) Å, *b* = 25.1362(14) Å, *c* = 10.6058(10) Å, *β* = 106.507(9)°, V = 2341.3(3) Å^3^, Z = 4, T = 100.0(3) K, *μ*(Cu K*α*) = 0.534 mm^−1^, D*_calc_* = 0.981 g/cm^3^, 31740 reflections measured (7.034° ≤ 2Θ ≤ 149.82°), 9035 unique (*R*_int_ = 0.0913, *R*_sigma_ = 0.0818), which were used in all calculations. The final *R*_1_ was 0.0918 (I > 2σ(I)) and *w*R_2_ was 0.2769 (all data).

Crystal Data for **10** C_21_H_30_O_4_ (M =346.45 g/mol): tetragonal, space group *P*4_1_2_1_2 (no. 92), *a* = 9.1899(2) Å, *c* = 53.8671(13) Å, V = 4549.3(2) Å^3^, Z = 8, T = 99.98(12) K, *μ*(Cu K*α*) = 0.549 mm^−1^, D*_calc_* = 1.012 g/cm^3^, 21859 reflections measured (6.564° ≤ 2Θ ≤ 148.182°), 4561 unique (*R*_int_ = 0.0410, *R*_sigma_ = 0.0329), which were used in all calculations. The final *R*_1_ was 0.0708 (I > 2σ(I)) and *wR*_2_ was 0.2300 (all data).

### 3.5. DFT Calculations

The conformational analysis of **2** and **10** were performed in Sybyl 8.1 software using the MMFF94s force field, which afforded the conformers for **2** and **10** with an energy cutoff of 3.0 kcal/mol to the global minima. All of the obtained conformers were optimized using the B3LYP/6-31+G(d) level in a gas phase by using Gaussian09 software [27]. The Boltzmann populations of **2** and **10** were determined by DFT calculation at the B3LYP/6-31+G(d,p) level.

### 3.6. Antifungal Activity of Extracts of Medicopsis sp. SCSIO 40440

The antifungal activity against twenty phytopathogenic fungi, including *Altemaria solani*, *Alternaria tenuissima*, *Bipolaris sorokiniana*, *Botrytis cinerea* Pers, *Ceratobasidium cornigerum*, *Colletotrichum fructicola*, *Colletotrichum gloeosporioides*, *Dothiorella gregaria*, *Fusarium culmorum*, *Fusarium graminearum*, *Fusarium oxysporum*, *Fusarium oxysporum* f. sp. *cucumerinu*, *Fusarium oxysporum* f. sp. *momordicae*, *Fusarium oxysporum* f. sp. *vasinfectum*, *Fusarium solani* f. sp. *pisi*, *Haematonectria*, *Peronophythora litchii*, *Phoma herbarum*, *Physalospora piricola* Nose, and *Valsa mali* (Strains obtained from CAMS Collection Center of Pathogenic Microorganisms), was evaluated by the modified Kirby–Bauer disc diffusion method [28]. Pathogenic fungi were cultured on potato dextrose agar (PDA) plates. From the actively growing edge of each colony, 6 mm agar discs were punched out and placed in the center of a new PDA plate. The plates were incubated upside down at 28 °C for 1–2 days. A standard paper disk (6 mm) was placed at the edge of PDA agar plates, ~1 cm away from the fungal colony. Each paper disk was impregnated with 10 μL of freshly prepared extracts of *Medicopsis* sp. SCSIO 40440 at a concentration of 20 μg/μL. Dimethylsulfoxide (DMSO) was used as the negative control, while nystatin (10 μg/mL) was used as the positive control. The plates were incubated at 25 °C for 2–3 days. The inhibition zones were measured to evaluate the antifungal activity. The experiments were performed in triplicate. For each phytopathogenic fungi, antifungal activity was recorded and graded as 0–3 based on the zone of inhibition of DMSO and nystatin. Grade 0 indicated no inhibition zone was observed; grade 1 indicated an inhibition zone less than that of nystatin; grade 2 indicated that the inhibition zone approximates to that of nystatin; and grade 3 indicated that the inhibition zone was larger than that of nystatin.

### 3.7. Antifungal Activity of Isolated Compounds Against Fusarium Species

The in vitro antifungal effects of isolated compounds against *Fusarium* species were evaluated using either the Kirby–Bauer disc diffusion method [28] or the mycelium growth rate method [29,30]. The mycelium growth rate method is briefly described as follows: 50 mg of the tested compound was dissolved in 5 mL DMSO to prepare an initial solution with a concentration of 10 mg/mL, which was then diluted to 50, 25, 12.5, and 6.75 μg/mL in the PDA medium. DMSO was used as the negative control, while nystatin and hygromycin B (50 μg/mL) were used as a positive control. Activated pathogens were punched with a 6 mm diameter puncher and inoculated in the center of the medicated medium (PDA) in a 28 °C incubator for 3–4 days. Each experiment was run in triplicate. The antifungal activities were expressed by the inhibition rate of mycelium growth (*IR*) in the following Equation:

*IR* (%) = (*B* − *T*)/*B* × 100

where *B* and *T* represent the growth diameters of the colony of the blank control and the colony which was treated with compounds, respectively.

### 3.8. Virulence Assays

Compound **1** and the strain *Medicopsis* sp. SCSIO 40440 were both evaluated for the ability to control the disease through pathogenicity tests on roots of tissue culture-derived banana plantlets (Cavendish banana, AAA) at the 4–5 leaf stage. The isolate XJZ2 of *F. oxysporum* f. sp. *cubense* tropical race 4 (*Foc* TR4) was used as the target pathogen [31]. Banana root inoculation assays were performed in a growth chamber, as previously described [32]. Disease symptoms were assessed 30 days after inoculation. For each plantlet, the severity of disease was recorded as grades 1–4 based on the extent of the vascular discoloration in the rhizome. Grade 1 (with value = 0) indicated no vascular discoloration was observed; grade 2 (with value = 1) indicated a little vascular discoloration, expressed as brown dots; grade 3 (with value = 2) indicated that vascular discoloration accounted for up to 50%; and grade 4 (with value = 3) indicated that vascular discoloration was over 50% in the corm of the banana plantlet. Thirty plantlets were inoculated with each fungal strain in an inoculation experiment, and the experiments were performed in triplicate. The disease incidence was calculated as the percentage of symptomatic plantlets (grade 1–3) over the total number of inoculated plantlets. The disease index was calculated using the following formula: the disease index = ∑(the number of plantlets × the grade value) × 100/(the total number of plantlets × the maximum grade value). Statistical analyses were performed using the *t* test with *p* ≤ 0.01 indicating a significant difference.

### 3.9. Statistical Analysis

All data are expressed as mean ±SEM and analyzed with GraphPad Prism 8.0 software. A one-way analysis of variance (ANOVA) followed by a Tukey multiple comparison test was performed to compare the differences among three or more groups. *p* < 0.05 was considered statistically significant.

## 4. Conclusions

In summary, ten decalin carboxylic acids (**1**–**10**) were isolated from the mangrove endophytic fungus *Medicopsis* sp. SCSIO 40440. These compounds encompass five planar structural groups, comprising nine new stereoisomeric compounds (**2**–**10**). Interesting, **2** and **10** are a pair of *cis*-*trans*-fused ring epimers. DFT calculations revealed that the *cis*-fused decalin ring exhibits greater flexibility than the *trans*-fused decalin counterpart. Compound **1** exhibited exceptional antifungal activity against *Foc* TR4, surpassing the positive controls nystatin and hygromycin B, as well as the recently reported niphimycin C (EC_50_ 1.20 μg/mL) [33] and R-prothioconazole (EC_50_ 0.78 μg/mL) [34]. These findings show the potential of **1** as a potential lead for biocontrol against FWB.

## Figures and Tables

**Table 1:** ^1^H NMR data of **2**–**10**.

No.	2 *^a,c^*	3 *^a,c^*	4 *^a,c^*	5 *^b,d^*	6 *^a,c^*	7 *^a,c^*	8 *^a,c^*	9 *^b,d^*	10 *^b,d^*
	*δ*_H_, mult. (*J* in Hz)	*δ*_H_, mult. (*J* in Hz)	*δ*_H_, mult. (*J* in Hz)	*δ*_H_, mult. (*J* in Hz)	*δ*_H_, mult. (*J* in Hz)	*δ*_H_, mult. (*J* in Hz)	*δ*_H_, mult. (*J* in Hz)	*δ*_H_, mult. (*J* in Hz)	*δ*_H_, mult. (*J* in Hz)
2	5.84, d (15.0)	5.79, d (15.5)	5.81, overlap	5.84, d (15.4)	5.79, d (15.3)	5.84, d (15.5)	5.88, d (15.5)	5.84, d (15.4)	5.82, d (15.3)
3	7.31, dd (15.4, 11.4)	7.11, dd (14.5, 10.8)	7.30, dd (15.0, 11.2)	7.27, dd (15.0, 10.5)	7.24, dd (13.8, 11.5)	7.28, dd (11.4, 15.5)	7.25, dd (15.5, 10.2)	7.17, dd (15.1, 11.1)	7.27, dd (15.3, 10.6)
4	6.27, dd (14.9, 11.3)	6.21, dd (15.0, 11.5)	6.27, dd (14.3, 10.7)	6.27, dd (14.3, 11.0)	6.26, dd (14.9, 11.4)	6.28, dd (10.5, 15.0)	6.35, dd (15.5, 9.8)	6.32, dd (14.9, 11.1)	6.26, overlap
5	6.47, dd (15.0, 10.4)	6.01, br s	6.47, dd (14.9, 10.5)	6.49, br s	6.40, br s	6.37, m	6.37, dd (15.5, 9.8)	6.46, dd (15.3, 10.7)	6.26, overlap
6	2.80, s	n	2.83, br s	2.83, overlap	2.97, s	2.95, br s	2.87, m	2.73, m	2.55, m
7	1.32, m	1.34, br s	1.29, br s	1.85, br s	1.34, m	1.40, br s	1.22, m	1.15, m	1.41, overlap
8	1.70, s	1.85, br s	1.42, br s	1.63, overlap	1.39, o	1.59, br s	1.35, m	1.21, overlap	1.40, overlap
9	1.06, m	1.09 br s;	3.11, dt (4.2, 10.5)	3.89, br s	1.02, m;	n	1.64, overlap	1.52, m;	1.17, m
9	1.82, dt (12.9, 3.9)	1.66 br s			1.64, m		1.01, overlap	0.93 m	1.83, m
10	3.42, ddd (12.9, 9.6, 4.2)	3.64, br s	1.46, q (10.5)	1.79, overlap	1.39, m;	n	1.60, overlap	1.48, overlap	3.39, ddd (11.2, 8.3, 4.2)
			1.95, dtd (10.6, 4.0, 1.0)	1.58, overlap	1.64, m		1.33, overlap	1.16, dd (12.5, 3.7)	
11	3.36, dd (5.5, 9.6)	3.57, br s	3.70, dt (10.5, 3.7)	4.09, br s	3.64, m	3.71, m	3.61, td (11.2, 4.5)	3.43	2.96, dd (10.8, 8.6)
12	2.86, s	2.42, br s	2.72, s	2.72, br s	2.78, m	2.76, br s	2.71, s	2.60, m	1.78, m
13	5.85, s	5.67, br s	5.84, br s	5.74, br s	5.76, s	6.13, br s	5.81, br s	5.72, br s	5.98, m
15	n	n	3.43, br s	3.41, br s	3.02, m	n	2.17, s	2.05, s	3.24, d (4.3)
17	5.36, m	5.34, br s	5.38, brs	5.38,	5.53, m	5.38, q (6.4)	2.82, q (6.0)	2.79, q (5.4)	5.44, dq (6.3. 1.3)
18	1.65, d (6.6)	1.49, br s	1.65, d (7.5)	1.62, overlap	1.64, overlap	1.63, overlap	1.41, d (5.7)	1.23, d (6.5)	1.48, d (6.2)
19	1.01, d (6.6)	0.94, d (6.59)	1.09, d (6.2)	1.00, d (5.94)	0.97, d (5.7)	1.00, d (6.0)	0.91, d (6.3)	0.81, d (6.4)	1.00, d (5.9)
20	1.59, s	1.49, overlap	1.61, s	1.79, overlap	1.64, overlap	3.94, d (13.0)	1.82, s	1.68, s	1.52, s
21	1.55, s	1.53, overlap	1.56, s	1.59, overlap	3.91, br s	3.87, d (13.0)	1.19, s	1.10, s	1.67, m

*^a^* Data were recorded on Bruker Avance 700 MHz NMR spectrometer; *^b^* on Bruker Avance 500 MHz NMR spectrometer; *^c^* in acetone-*d*_6_, *^d^* in methonal-*d*_4_, with TMS as an internal standard; n, signals not observed; the signals were assigned with the aid of COSY, HSQC, and HMBC data.

**Table 2:** ^13^C NMR data of **2**–**10**.

No.	2 *^a,c^*	3 *^a,c^*	4 *^a,c^*	5 *^b,d^*	6 *^a,c^*	7 *^a,c^*	8 *^a,c^*	9 *^b,d^*	10 *^b,d^*
	*δ*_C_, Type	*δ*_C_, Type	*δ*_C_, Type	*δ*_C_, Type	*δ*_C_, Type	*δ*_C_, Type	*δ*_C_, Type	*δ*_C_, Type	*δ*_C_, Type
1	167.1, C	168.4, C	167.1, C	169.4, C	168.2, C	167.7, C	171.2, C	168.6, C	168.0, C
2	119.8, CH	120.8, CH	119.8, CH	121.8, CH	120.3, CH	120.3, CH	122.6, CH	122.4, CH	120.0, CH
3	145.2, CH	144.8, CH	145.2, CH	146.3, CH	146.2, CH	144.8, CH	145.6, CH	144.3, CH	146.0, CH
4	128.3, CH	128.7, CH	128.3, CH	130.1, CH	129.4, CH	128.4, CH	129.9, CH	129.0, CH	127.2, CH
5	146.7, CH	147.7, CH	146.9, CH	148.0, CH	147.8, CH	146.8, CH	147.3, CH	147.0, CH	150.5, CH
6	43.2, CH	43.0, CH	43.1, CH	44.9, CH	44.0, CH	43.1, CH	42.3, CH	41.4, CH	49.7, CH
7	47.9, CH	44.7, CH	45.1, CH	43.1, CH	48.5, CH	47.2, CH	49.9, CH	48.6, CH	35.7, CH
8	28.1, CH	34.0, CH	36.9, CH	35.1, CH	29.8, CH	28.5, CH	29.9, CH	29.2, CH	45.7, CH
9	41.4, CH_2_	37.5, CH_2_	72.5, CH	73.1, CH	34.2, CH_2_	38.5, CH_2_	34.2, CH_2_	33.1, CH_2_	44.4, CH_2_
10	70.4, CH	67.3, CH	40.2, CH_2_	39.4, CH_2_	31.5, CH_2_	38.9, CH_2_	31.1, CH_2_	31.3, CH_2_	75.1, CH
11	76.8, CH	72.0, CH	69.0, CH	67.8, CH	72.5, CH	70.9, CH	73.3, CH	71.1, CH	78.4, CH
12	37.5, CH	36.4, CH	37.7, CH	39.3, CH	38.6, CH	37.3, CH	38.9, CH	38.2, CH	47.6, CH
13	122.7, CH	122.8, CH	122.7, CH	126.3, CH	122.4, CH	123.2, CH	125.0, CH	124.2, CH	125.1, CH
14	133.3, C	135.5, C	134.1, C	135.0, C	136.0, C	139.3, C	134.9, C	132.1, C	134.4, C
15	41.1, CH	n	41.3, CH	n	41.9, CH	n	45.5, CH	40.8, CH	47.1, CH
16	135.6, C	135.5, C	135.7, C	137.0, C	139.4, C	135.0, CH	62.7, C	60.9, C	134.8, C
17	122.5, CH	125.8, CH	122.6, CH	124.4, CH	123.3, CH	122.7, CH	59.4, CH	62.0, CH	124.3, CH
18	12.5, CH_3_	13.6, CH_3_	12.4, CH_3_	14.3, CH	13.1, CH_3_	12.7, CH_3_	14.7, CH_3_	15.0, CH_3_	13.7, CH_3_
19	18.7, CH_3_	20.3, CH_3_	14.2, CH_3_	16.6, CH_3_	19.5, CH_3_	18.7, CH_3_	19.6, CH_3_	19.1, CH_3_	23.2, CH_3_
20	21.2, CH_3_	22.4, CH_3_	21.3, CH_3_	23.1, CH_3_	22.2, CH_3_	64.6, CH_2_	22.5, CH_3_	22.2, CH_3_	21.5, CH_3_
21	21.6, CH_3_	22.7, CH_3_	21.3, CH_3_	23.2, CH_3_	68.9, CH_2_	22.1, CH_3_	22.7, CH_3_	21.9, CH_3_	22.9, CH_3_

*^a^* Data were recorded on Bruker Avance 175 MHz NMR spectrometer; *^b^* on Bruker Avance 125 MHz NMR spectrometer; *^c^* in acetone-*d*_6_; *^d^* in methonal-*d*_4_, with TMS as an internal standard. n, signal not observed; the signals were assigned with the aid of COSY, HSQC, and HMBC data.

## Data Availability

The authors declare that all relevant data supporting the findings of this study are available within the article and its Supplementary Materials files or from the corresponding authors upon request.

## Supplementary Materials

The following supporting information can be downloaded at: https://www.mdpi.com/article/10.3390/md23020088/s1, Figure S1: Spectral data for MK8383 (**1**); Figure S2: Spectral data for MK8383 B (**2**); Figure S3: Spectral data for 10-*epi*-MK8383 B (**3**) (**1**); Figure S4: Spectral data for MK8383 C (**4**); Figure S5: Spectral data for 9-*epi*-MK8383 C (**5**); Figure S6: Spectral data for MK8383 D (**6**); Figure S7: Spectral data for MK8383 E (**7**); Figure S8: Spectral data for MK8383 F (**8**); Figure S9: Spectral data for MK8383 G (**9**); Figure S10: Spectral data for MK8383 I (**10**); Figure S11: Antifungal activities of compounds **1**, **2**, **4**, **6**, and **10**; Figure S12: EC_50_ of nystatin and hygromycin B on mycelial growth of *Foc* TR4; Table S1: Antibacterial Activity grade of the extract of *M*. sp. SCSIO 40440 against twenty phytopathogenic fungi; Table S2: ^1^H and ^13^C NMR data of MK8383 (**1**) in acetone-d6; Table S3: H-9_eq_ and H-9_ax_ of MK8383B (**2**) and 10-*epi*-MK8383 B (**3**) in acetone-*d*_6_; Table S4: X-ray crystallographic data of MK8383 B (**2**) and I (**10**); Table S5: H-10_eq_ and H-10_ax_ of MK8383 C (**4**) and 9-*epi*-MK8383 C (**5**) in acetone-*d*_6_; Table S6: Disease severity expressed by disease incidence and disease index; check CIF report of **2**; check CIF report of **10**.
