# Supplementary material for: Discovery of MK8383s with Antifungal Activity from Mangrove Endophytic Fungi Medicopsis sp. SCSIO 40440 Against Fusarium Wilt of Banana
Source: Mar Drugs. 2025 Feb 18;23(2):88. doi: 10.3390/md23020088 (PMC11857101; doi:10.3390/md23020088)
Supplement: Supplementary file 1 [file marinedrugs-23-00088-s001.zip › marinedrugs-3475196-supplementary.pdf]

Supporting Information

Discovery of MK8383s with Antifungal Activity from mangrove *endophytic* fungi *Medicopsis* sp. SCSIO 40440 Against Fusarium Wilt of Banana

Tianyu Zhou, Yulei Qiao *et al*.

Content

Table S1. Antibacterial Activity grade of the extract of *M*. sp. SCSIO 40440 against twenty phytopathogenic fungi..S3
Table S2. ^{1}H and ^{13}C NMR data of MK8383 (**1**) in acetone-d_6. ....S5
Table S3. H-9$_{eq}$ and H-9$_{ax}$ of MK8383B (**2**) and 10-epi-MK8383 B (**3**) in acetone-d_6. ....S6
Table S4. X-ray crystallographic data of MK8383 B (**2**) and I (**10**). S7
Table S5. H-10$_{eq}$ and H-10$_{ax}$ of MK8383 C (**4**) and 9-*epi*-MK8383 C (**5**) in acetone-d_6. ....S8
Table S6. Disease severity expressed by disease incidence and disease index. S9
Figure S1. Spectral data for MK8383 (**1**)...S10
Figure S2. Spectral data for MK8383 B (**2**). S14
Figure S3. Spectral data for 10-*epi*-MK8383 B (**3**). S21
Figure S4. Spectral data for MK8383 C (**4**). S28
Figure S5. Spectral data for 9-*epi*-MK8383 C (**5**). S35
Figure S6. Spectral data for MK8383 D (**6**)..S42
Figure S7. Spectral data for MK8383 E (**7**). S49
Figure S8. Spectral data for MK8383 F (**8**). S56
Figure S9. Spectral data for MK8383 G (**9**)..S63
Figure S10. Spectral data for MK8383 I (**10**). S70
Figure S11. Antifungal activities of compounds **1**, **2**, **4**, **6**, and **10.**..........................S77

Table S1. Antibacterial Activity grade of the extract of *M*. sp. SCSIO 40440 against twenty phytopathogenic fungi.

	A	B	C	D	E	F	G	H	I	J
**-**	0	2	0	0	0	0	0	0	0	0
+	2	2	2	2	0	0	0	2	2	2
**1**	1	2	3	2	3	3	3	2	3	3
	**K**	**L**	**M**	**N**	**O**	**P**	**Q**	**R**	**S**	**T**
**-**	0	0	0	0	0	0	0	0	0	0
+	2	2	2	2	0	0	0	2	2	2
**1**	3	2	1	3	3	3	0	2	3	1

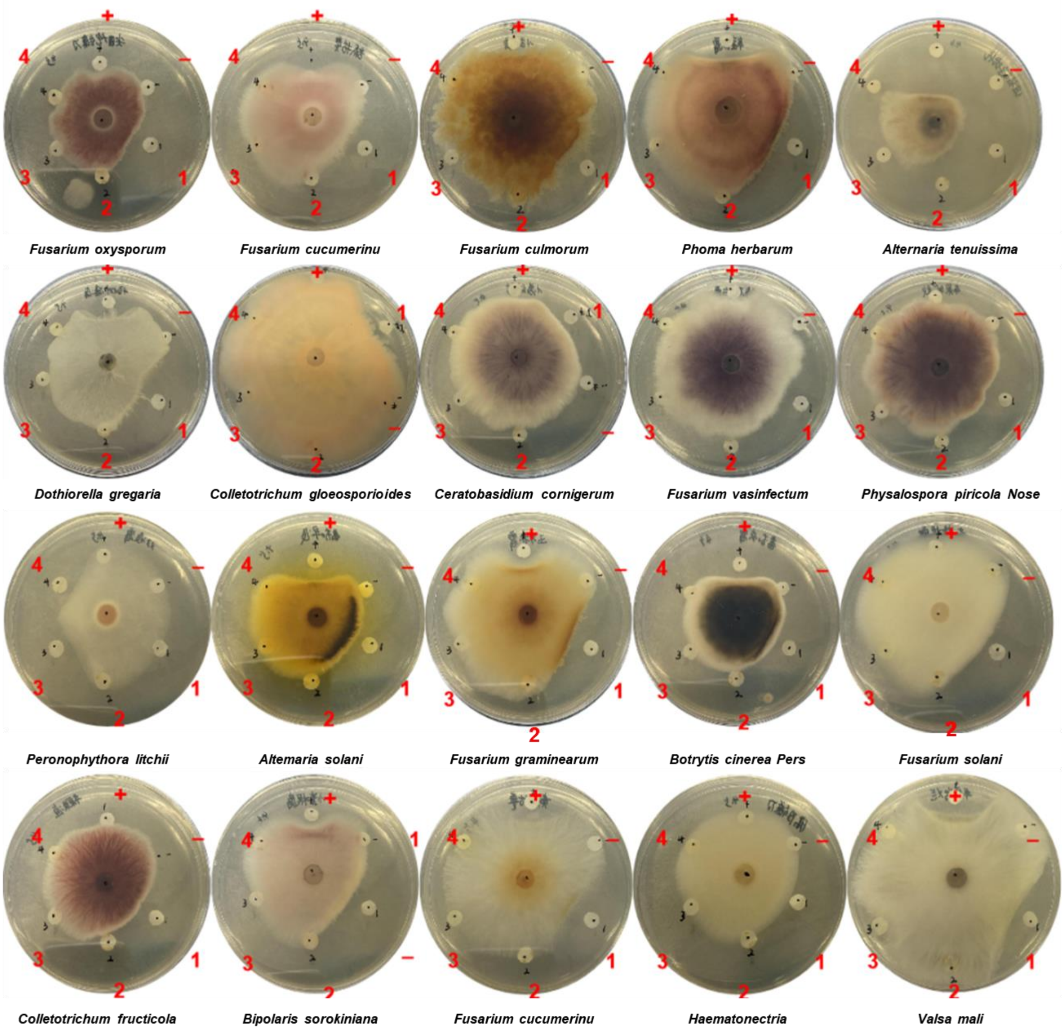

Antifungal activity was assessed for each fungus and graded from 0–3 based on the zone of inhibition compared to DMSO and nystatin. Grade 0: no inhibition; Grade 1: inhibition zone smaller than nystatin; Grade 2: inhibition zone comparable to nystatin; Grade 3: inhibition zone larger than nystatin.

A: *Altemaria solani*; B: *Alternaria tenuissima*; C: *Bipolaris sorokiniana*; D:*Botrytis cinerea* Pers; E: *Ceratobasidium cornigerum*; F: *Colletotrichum fructicola*; G: *Colletotrichum gloeosporioides*; H: *Dothiorella gregaria*; I: *Fusarium culmorum*; J: *Fusarium graminearum*; K: *Fusarium oxysporum*; L: *Fusarium cucumerinu*; M: *Fusarium momordicae*; N: *Fusarium vasinfectum*; O: *Fusarium solani*; P: *Haematonectria*; Q: *Peronophythora litchii*; R: *Phoma herbarum*; S: *Physalospora piricola Nose*; T: *Valsa mali*A; -: DMSO; +: nystatin; 1: extract of *M*. sp. SCSIO 40440. 2-4: the other extracts not mentioned in this study.

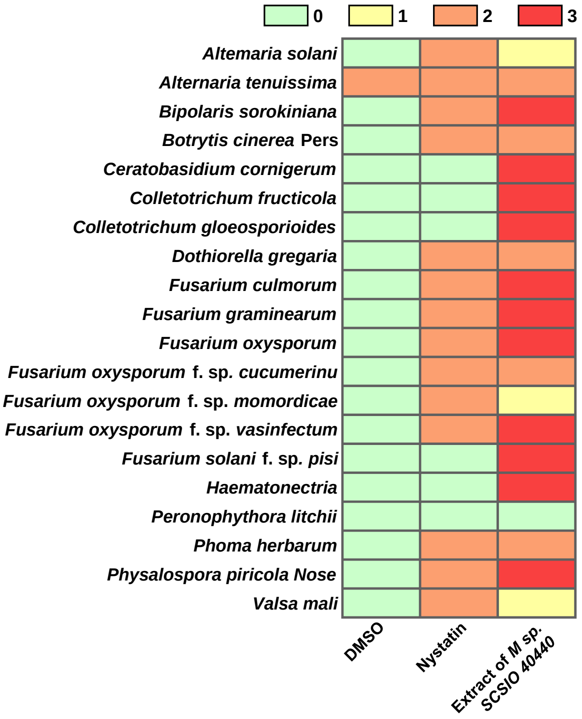

Antifungal activity of extracts from *M.* sp. SCSIO 40440 against twenty phytopathogenic fungi. The antifungal potential against various fungi was assessed using a modified Kirby-Bauer disc diffusion method and classified into four grades (0–3) based on the inhibition zone relative to DMSO and nystatin. Grade 0: no inhibition; Grade 1: inhibition zone smaller than nystatin; Grade 2: inhibition zone comparable to nystatin; Grade 3: inhibition zone larger than nystatin.

Table S2. ^{1}H and ^{13}C NMR data of MK8383 (**1**) in acetone-d_6.

No.	**1**	
	δ_C, type	δ_H, mult. (J in Hz)
1	167.3, C	
2	119.6, CH	5.82, d (15.7)
3	145.3, CH	7.27, dd (15.4, 11.3)
4	128.2, CH	6.27, dd (15.4, 11.3)
5	147.1, CH	6.41, br s
6	43.4, CH	2.85, br s
7	47.7, CH	1.32, br s
8	28.7, CH	1.51, overlapping
9	33.0, CH_2	1.02, m
		1.64, m
10	30.3, CH_2	1.64, m
		1.41, q (9.2)
11	71.2, CH	3.63, m
12	37.7, CH	2.71, br s
13	123.2, CH	5.80, br s
14	134.3, C	
15	41.8, CH	3.40, br s
16	135.6, C	
17	122.5, CH	5.37, m
18	12.4, CH_3	1.63, d (5.6)
19	18.7, CH_3	0.98, d (6.9)
20	21.3, CH_3	1.60, s
21	21.6, CH_3	1.56, br s

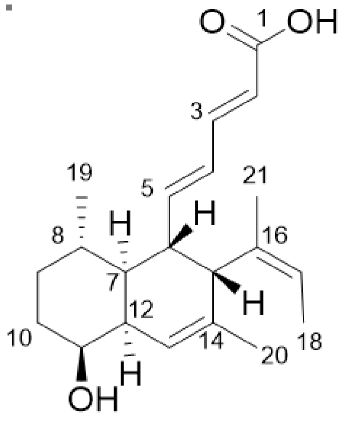

1

Table S3. H-9_{eq} and H-9_{ax} of MK8383B (**2**) and 10-epi-MK8383 B (**3**) in acetone-d_6.

No.	**2**	**3**
	$\delta_{H,}$ mult. (*J* in Hz)	$\delta_{H,}$ mult. (*J* in Hz)
9_{eq}	1.06, m	1.09 br s;
9_{ax}	1.82, dt (12.9, 3.9)	1.66 br s

Data were recorded on Bruker Avance 700 MHz for ^{1}H in acetone-d_6 with TMS as an internal standard.

Table S4. X-ray crystallographic data of MK8383 B (**2**) and I (**10**).

	**2**	**10**
Empirical formula	$C_{84}H_{118}O_{16}$	$C_{21}H_{30}O_4$
Formula weight	1383.78	346.45
Temperature/K	100.0(3)	99.98(12)
Crystal system	monoclinic	tetragonal
Space group	$P2_1$	$P4_12_12$
a/Å	9.1600(5)	9.1899(2)
b/Å	25.1362(14)	9.1899(2)
c/Å	10.6058(10)	53.8671(13)
α/°	90	90
β/°	106.507(9)	90
γ/°	90	90
Volume/$Å^3$	2341.3(3)	4549.3(2)
Z	4	1
ρ_{calc} g/cm^3	0.981	1.012
μ/mm^{-1}	0.534	0.549
F(000)	750.0	1504.0
Crystal size/mm^3	0.3 × 0.1 × 0.1	0.1 × 0.05 × 0.05
Radiation	Cu Kα (λ = 1.54184)	Cu Kα (λ = 1.54184)
2Θ range for data collection/ °	7.034 to 149.82	6.564 to 148.182
Index ranges	-11 ≤ h ≤ 11, -30 ≤ k ≤ 31, -13 ≤ l ≤ 12	-11 ≤ h ≤ 11, -10 ≤ k ≤ 9, -65 ≤ l ≤ 64
Reflections collected	31740	21859
Independent reflections	9035 [R_{int} = 0.0913, R_{sigma} = 0.0818]	4561 [R_{int} = 0.0410, R_{sigma} = 0.0329]
Data/restraints/parameters	9035/1/464	4561/0/233
Goodness-of-fit on F^2	1.075	1.058
Final R indexes [I>=2σ (I)]	R_1 = 0.0918, wR_2 = 0.2485	R_1 = 0.0708, wR_2 = 0.2091
Final R indexes [all data]	R_1 = 0.1275, wR_2 = 0.2769	R_1 = 0.0920, wR_2 = 0.2300
Largest diff. peak/hole / e $Å^{-3}$	0.40/-0.34	0.35/-0.20
Flack parameter	0.1(2)	0.06(11)

Table S5. H-10_{eq} and H-10_{ax} of MK8383 C (**4**) and 9-*epi*-MK8383 C (**5**) in acetone-d_6.

No.	**4**	**5**
	$\delta_{H,}$ mult. (J in Hz)	$\delta_{H,}$ mult. (J in Hz)
10_{eq}	1.95, dtd (10.6, 4.0, 1.0)	1.79, overlap
10_{ax}	1.46, q (10.5)	1.58, overlap

Data were recorded on Bruker Avance 700 MHz for ^{1}H in acetone-d_6 with TMS as an internal standard.

Table S6. Disease severity expressed by disease incidence and disease index.

Groups	Grade				Total	Disease Index
	**0**	**1**	**2**	**3**		
**CK**	25	0	0	0	25	0
**A**	26	0	0	0	26	0
**B**	25	0	0	0	25	0
**C**	1	11	5	6	28	0.64
**D**	19	5	3	0	27	0.14
**E**	14	3	3	5	29	0.34

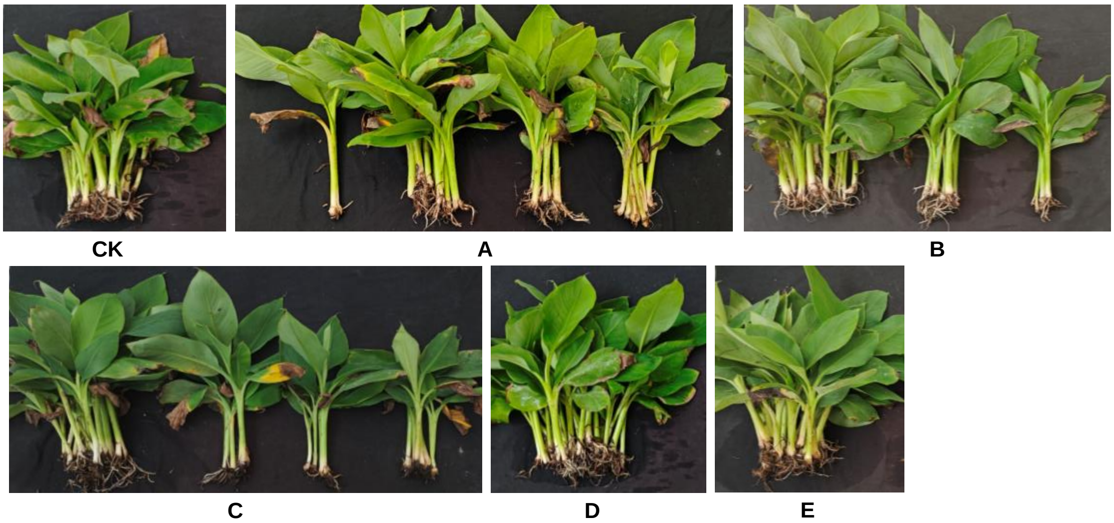

Disease incidence was calculated as the percentage of symptomatic plantlets over total number of inoculated plantlets. Disease index was calculated using the following formula: Disease index = Σ(number of plantlets × grade value) × 100/(total number of plantlets × maximum grade value). Statistical analyses were performed using t test with $p \leqslant 0.01$ indicating a significant difference. CK: water; A: treated with compound **1**; B: treated with spores of SCSIO 40440; C: treated with spores of race 4; D: treated with spores of race 4 and compound **1**; E: treated with spores of race 4 and SCSIO 40440. Disease symptom on corms of banana tissue plantlets which were recorded at 30 days after inoculation with compound **1**, spores of SCSIO 40440 and negative control of water. CK: water; A: treated with spores of race 4; B: treated with spores of race 4 and compound **1**; C: treated with spores of race 4 and SCSIO 40440; D: treated with compound **1**; E: treated with spores of SCSIO 40440;.

Figure S1. Spectral data for MK8383 (**1**).

(A) HRESIMS

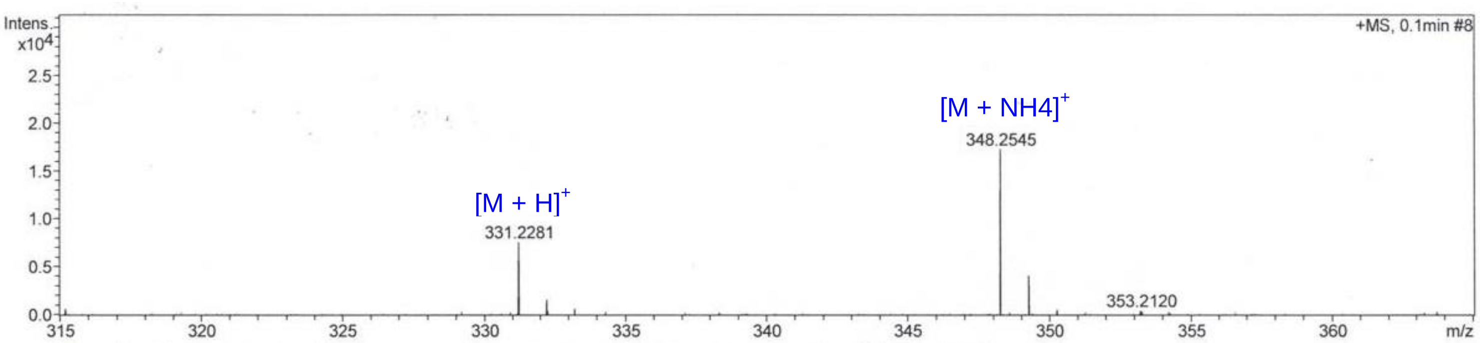

Meas. m/z	#	Ion Formula	Score	m/z	err [ppm]	err [mDa]	mSigma	rdb	e⁻ Conf	N-Rule
331.2281	1	C21H31O3	100.00	331.2268	3.9	1.3	36.8	6.5	even	ok
348.2545	1	C21H34NO3	100.00	348.2533	3.5	1.2	0.9	5.5	even	ok

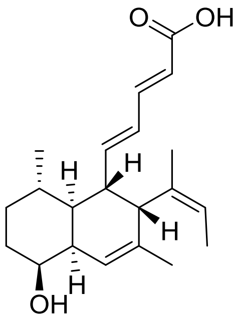

1

Chemical Formula: $C_{21}H_{30}O_3$

Exact Mass: 330.2

Figure S1. Spectral data for MK8383 (**1**) (continued).

(B) The ^{1}H NMR spectrum of MK8383 (**1**) in four kind of NMR solvents (DMSO-d_6, Pyrdine-d_5, Actone-d_6, Chloroform-d_1).

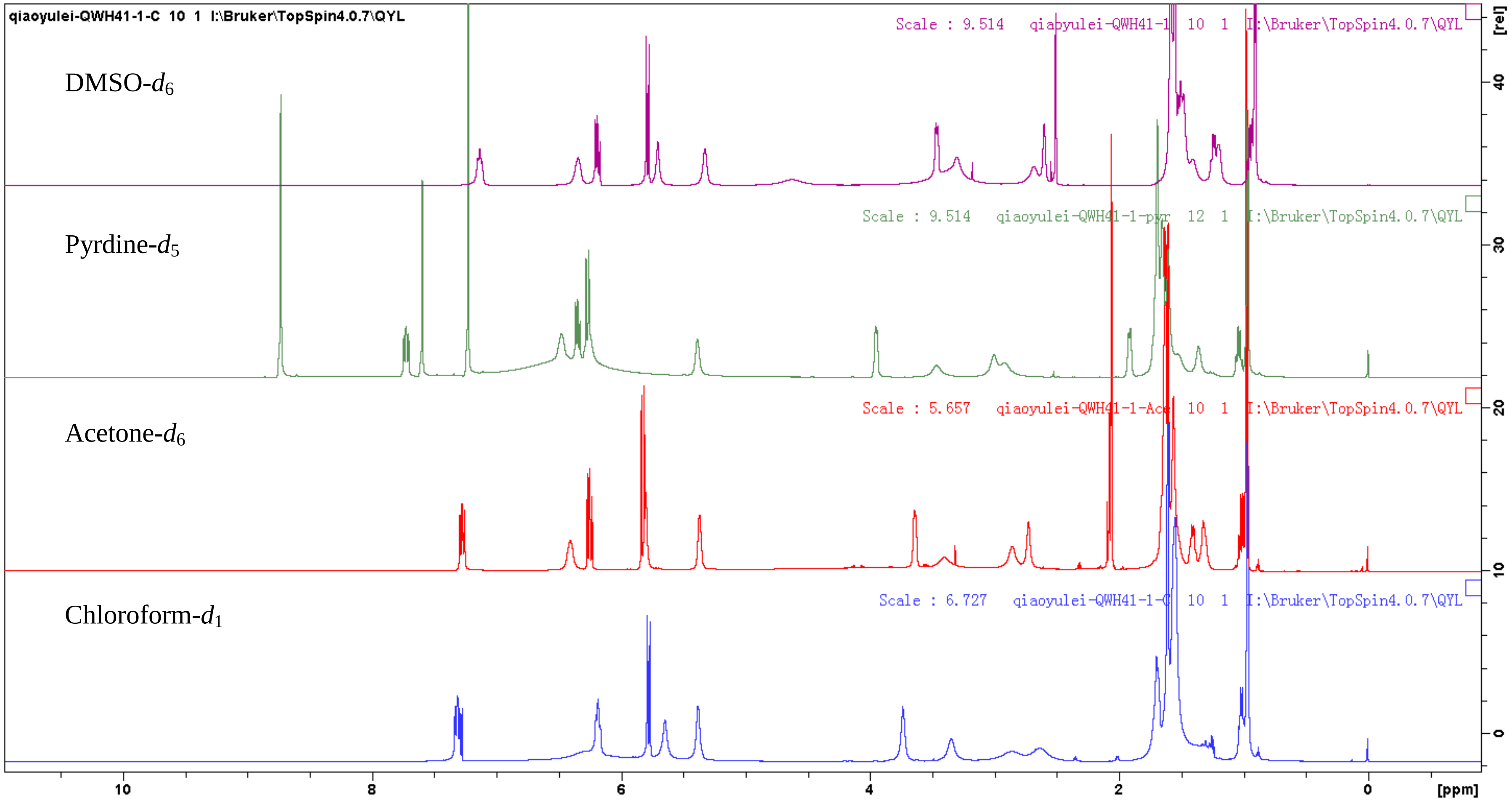

Figure S1. Spectral data for MK8383 (**1**) (continued).

(C) The ^{1}H NMR spectrum of MK8383 (**1**) in Actone-d_6.

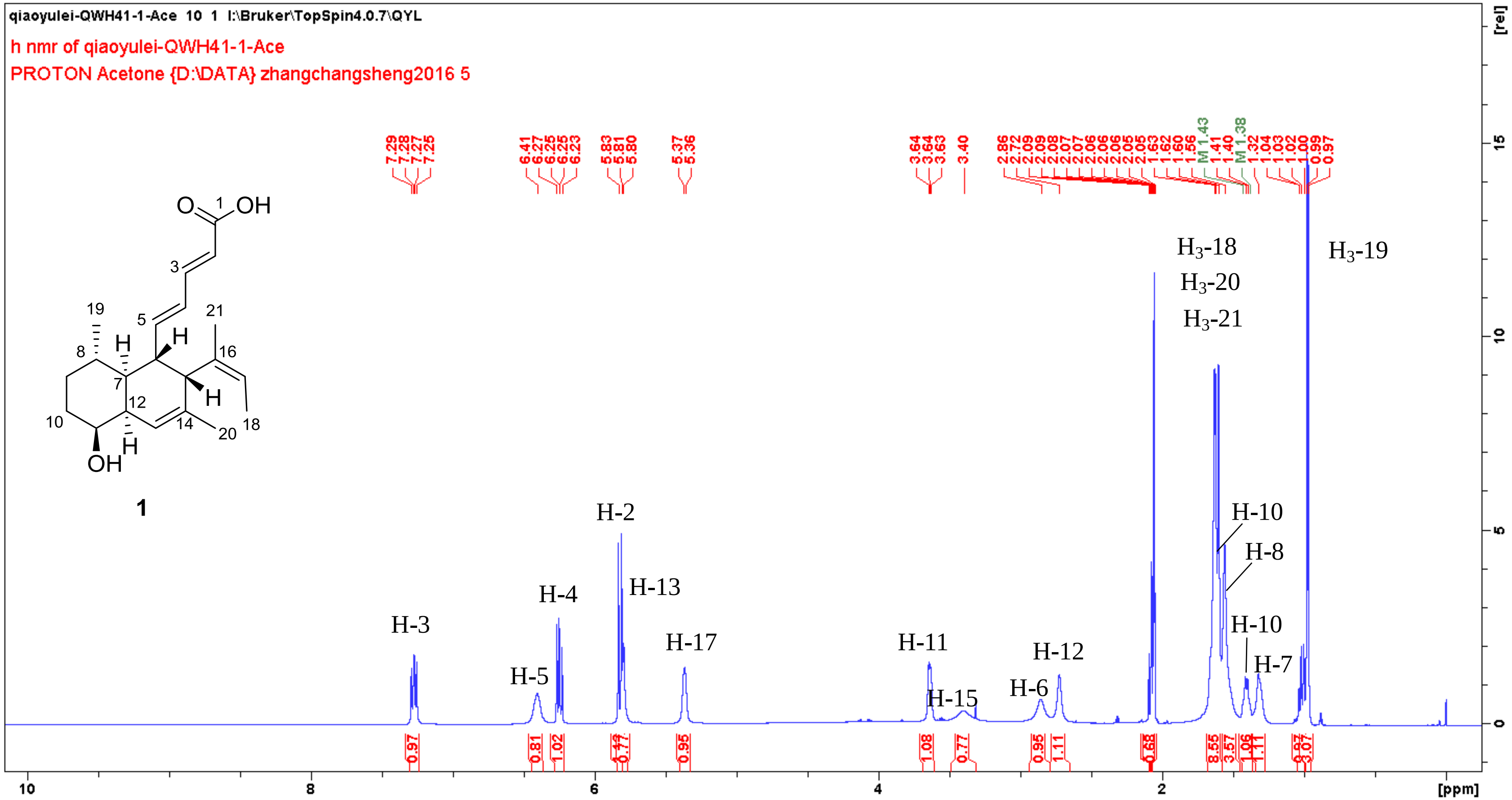

Figure S1. Spectral data for MK8383 (**1**) (continued).
(D) The ^{13}C NMR spectra of MK8383 (**1**) in Actone-d_6.

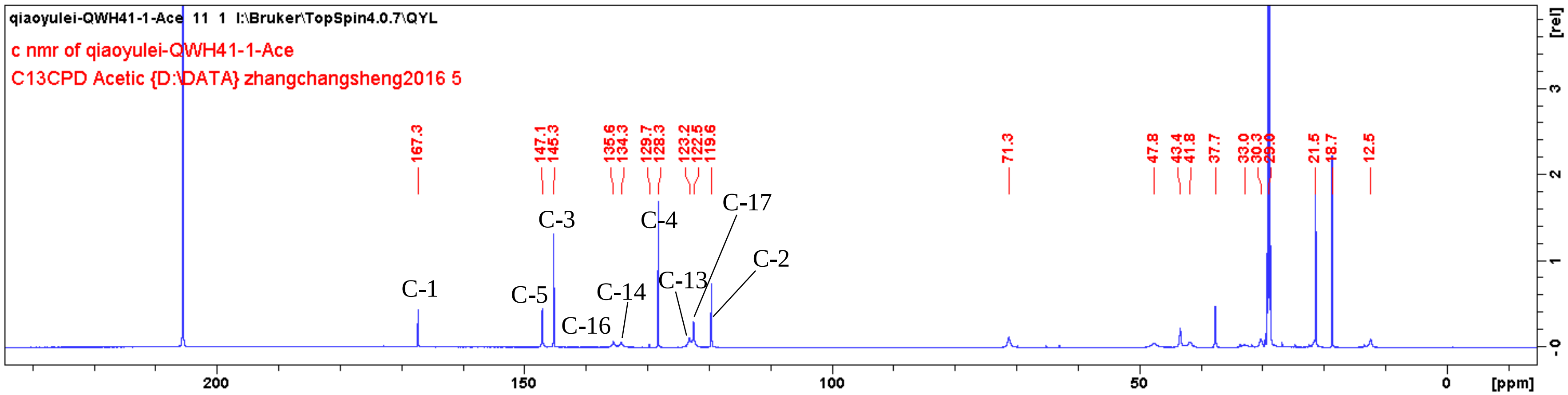

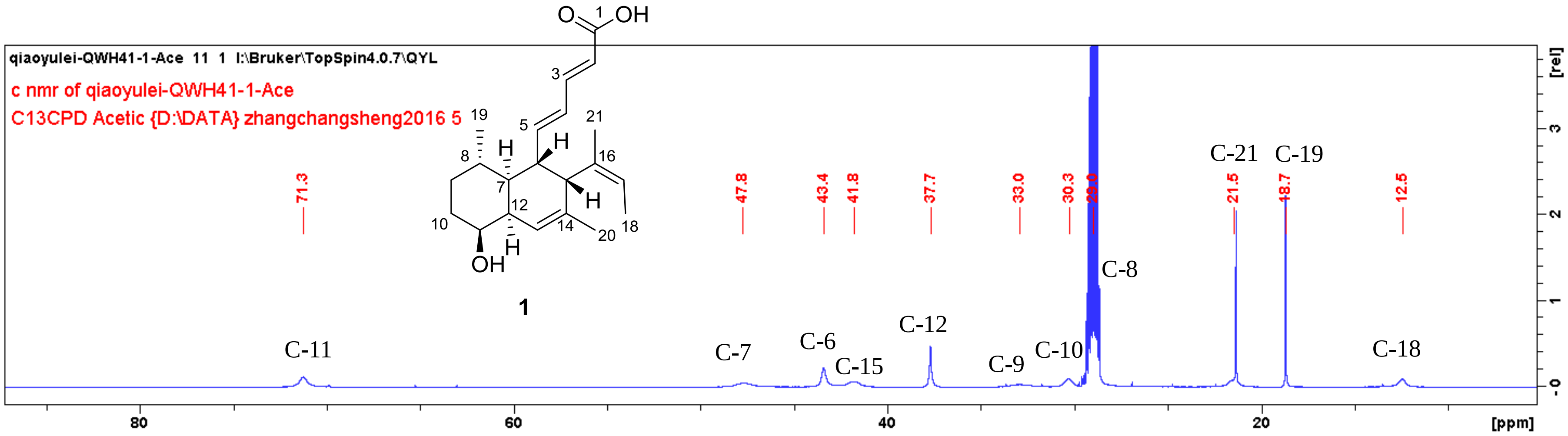

Figure S2. Spectral data for MK8383 B (**2**).

(A) HR-ESI-MS

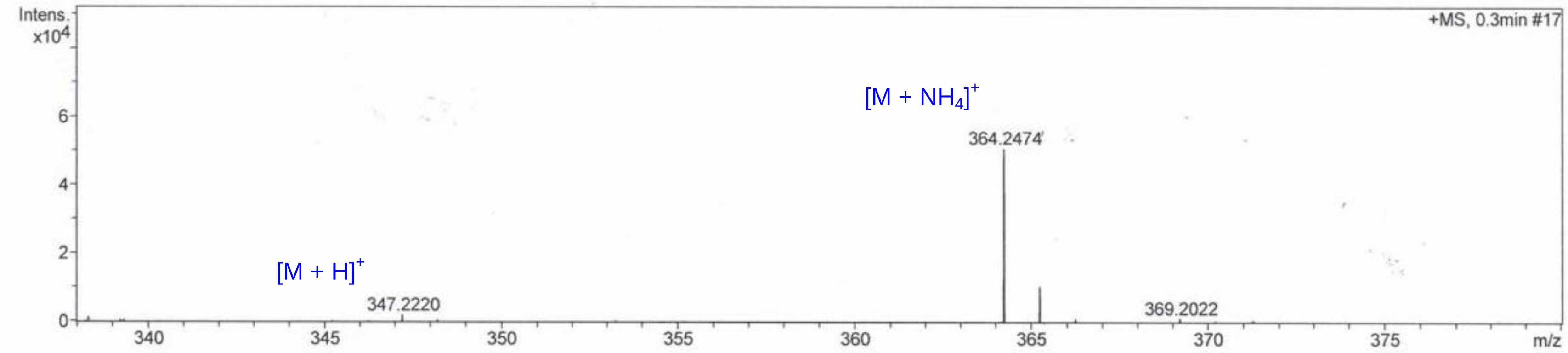

Meas. m/z	#	Ion Formula	Score	m/z	err [ppm]	err [mDa]	mSigma	rdb	e⁻ Conf	N-Rule
347.2220	1	C21H31O4	100.00	347.2217	0.8	0.3	23.3	6.5	even	ok
364.2474	1	C21H34NO4	100.00	364.2482	-2.2	-0.8	18.0	5.5	even	ok

(**B**) UV

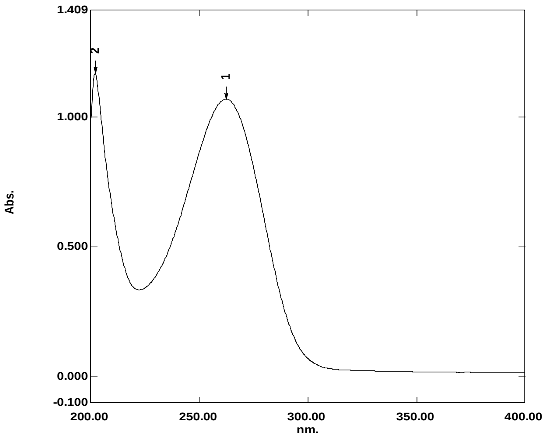

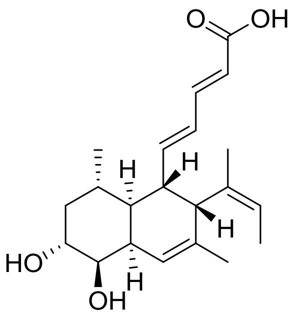

2

Chemical Formula: $C_{21}H_{30}O_4$

Exact Mass: 346.2

(**C**) IR

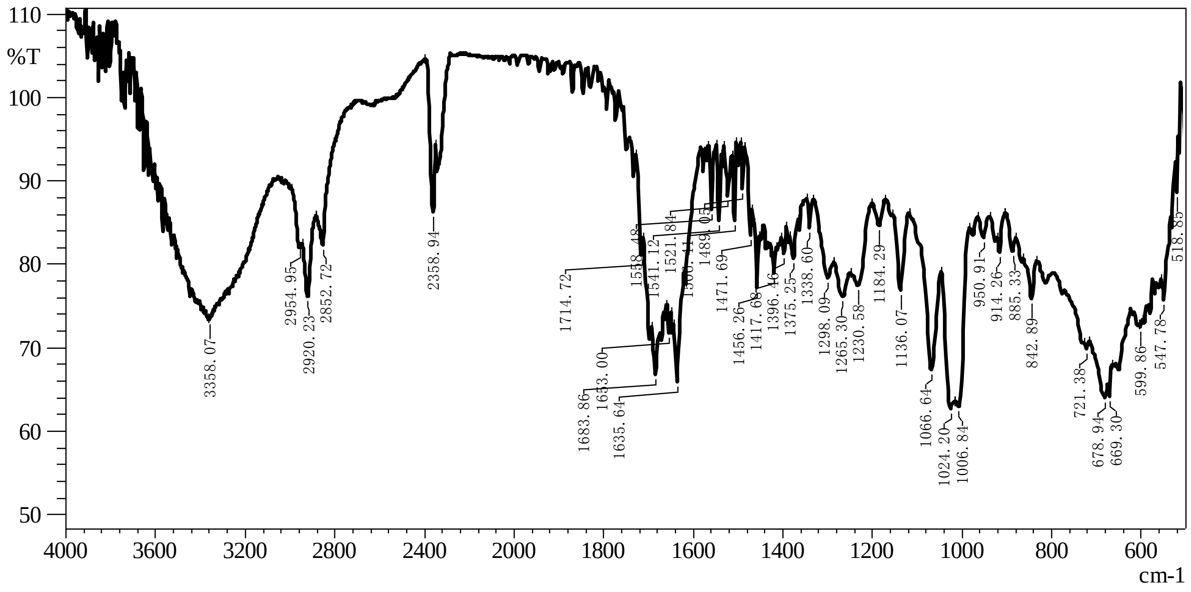

Figure S2. Spectral data for MK8383 B (**2**) (continued).

(D) The ^{1}H and enlarged ^{1}H NMR spectrum of MK8383B (**2**) in Acetone-d_6.

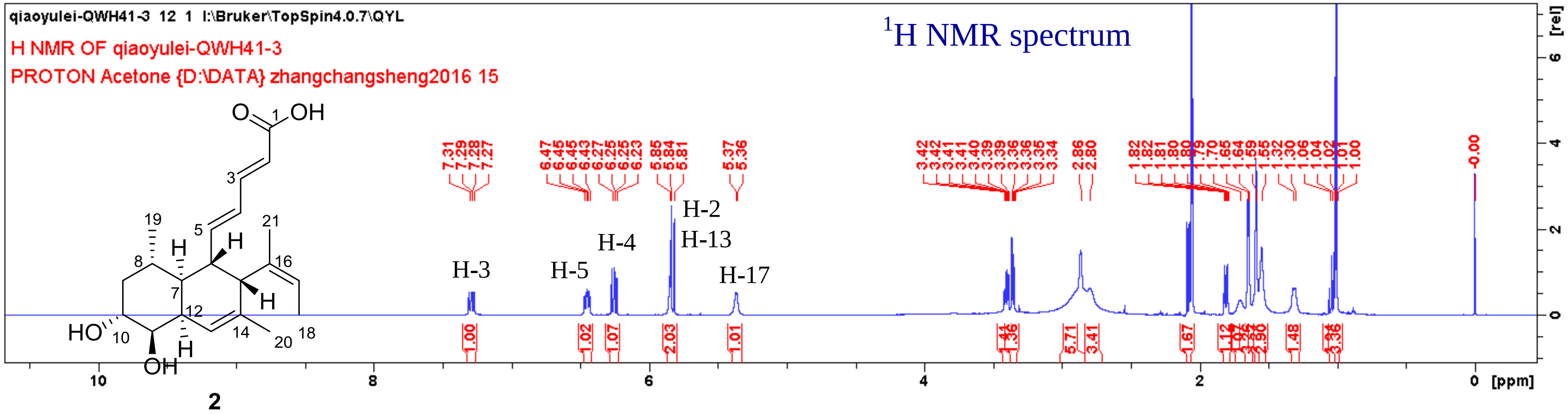

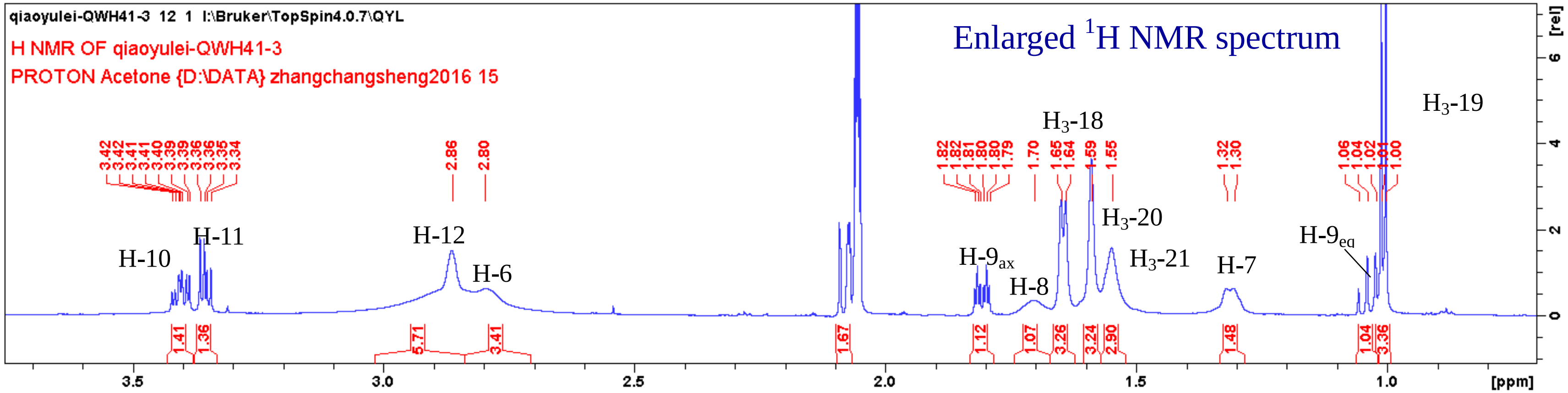

Figure S2. Spectral data for MK8383 B (**2**) (continued).

(E) The ^{13}C and enlarged ^{13}C NMR spectrum of MK8383B (**2**) in Acetone-d_6.

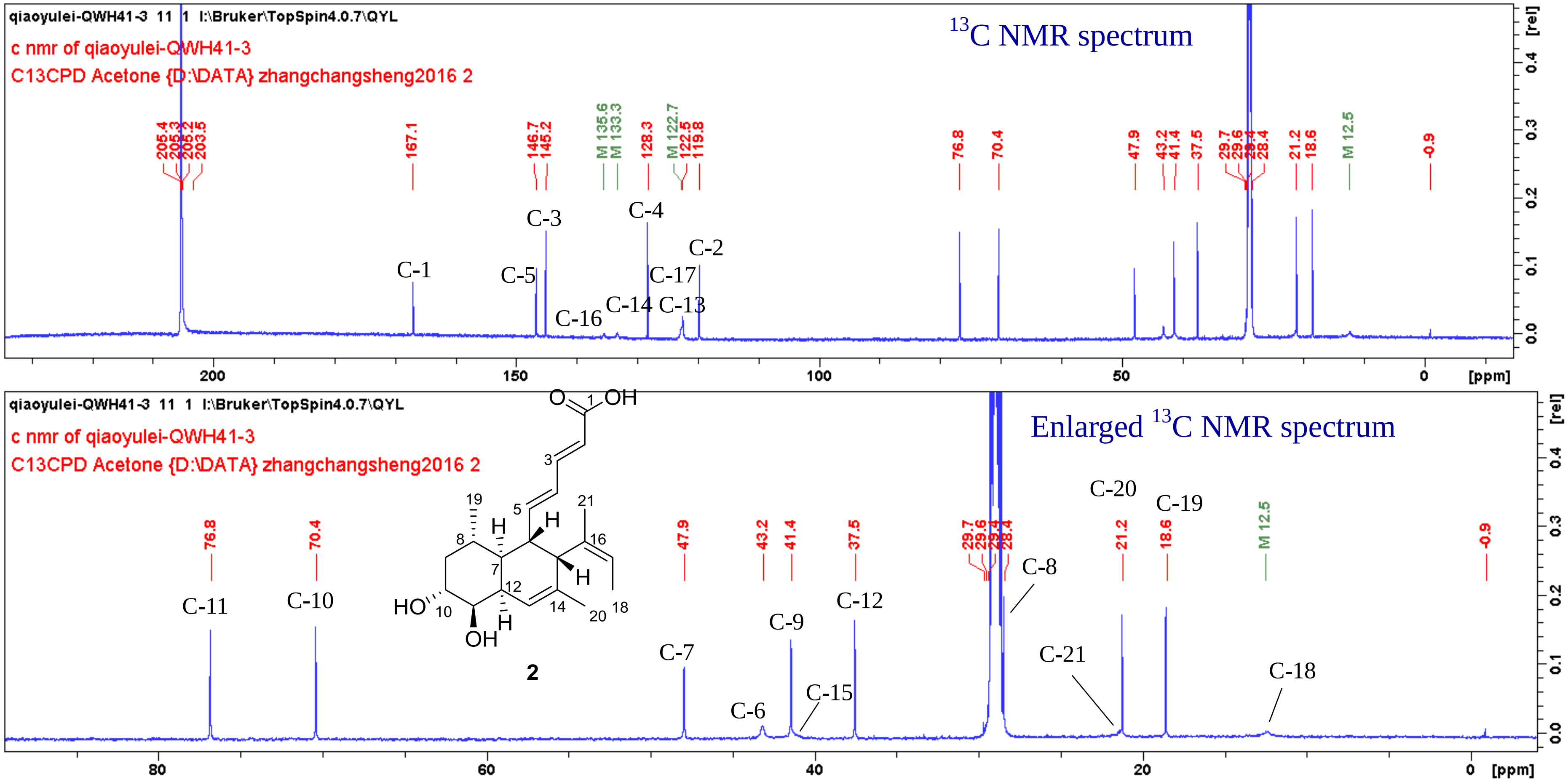

Figure S2. Spectral data for MK8383 B (**2**) (continued).
(F) The HSQC spectrum of MK8383 B (**2**) in Acetone-d_6.

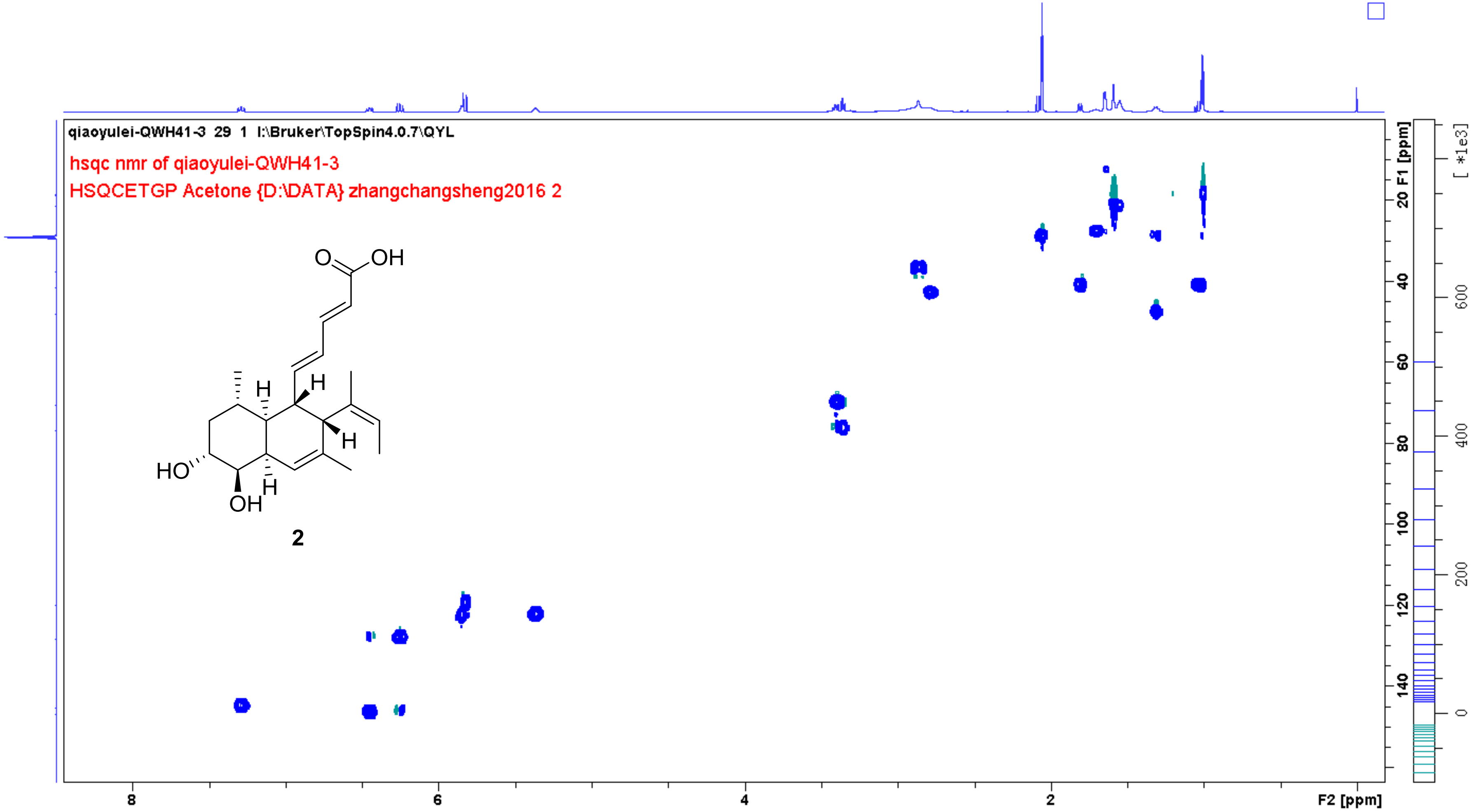

Figure S2. Spectral data for MK8383 B (**2**) (continued).

(G) The ^{1}H-^{1}H COSY spectrum of MK8383 B (**2**) in Acetone-d_6.

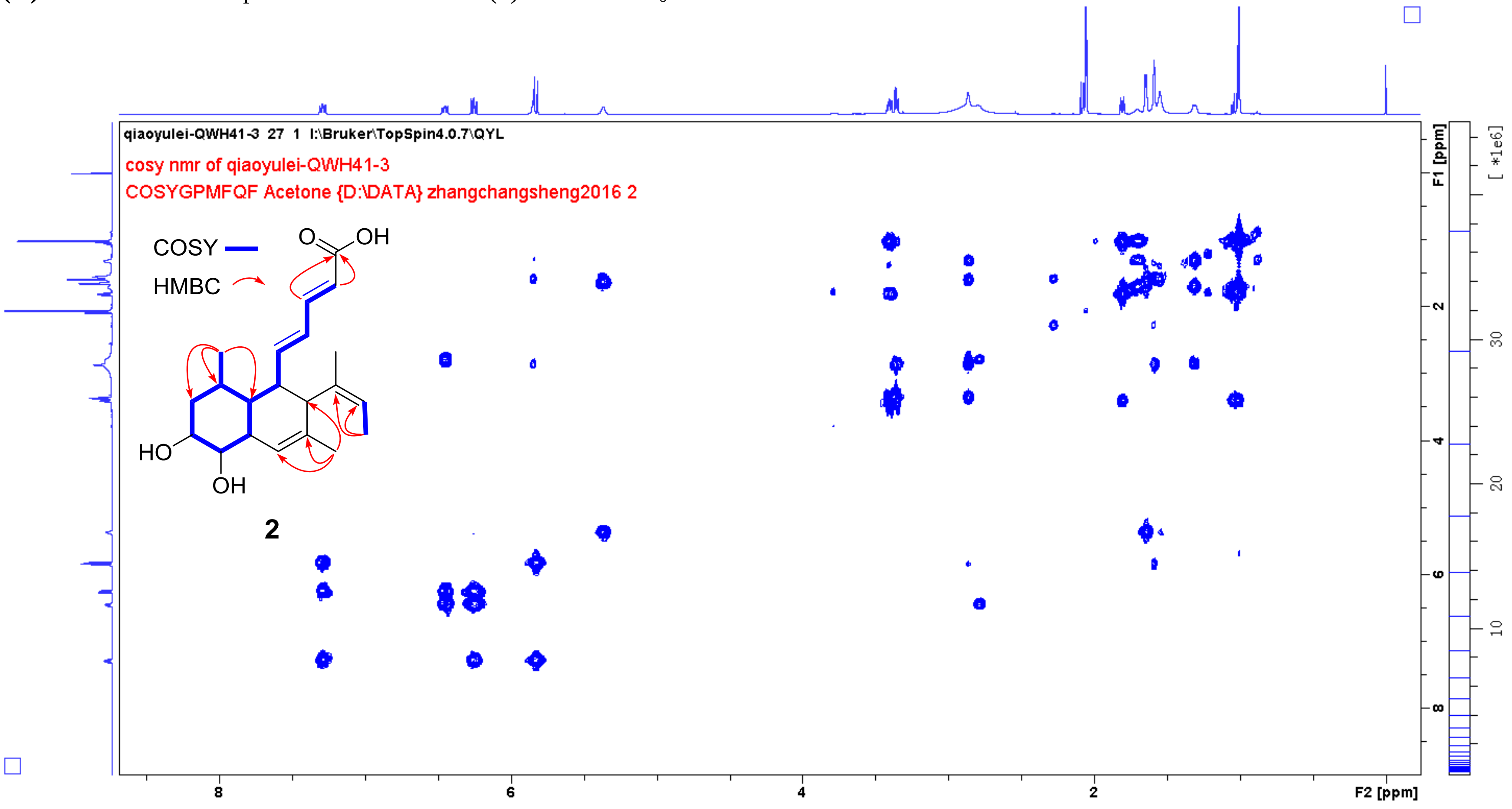

Figure S2. Spectral data for MK8383 B (**2**) (continued).
(H) The HMBC spectrum of MK8383 B (**2**) in Acetone-d_6.

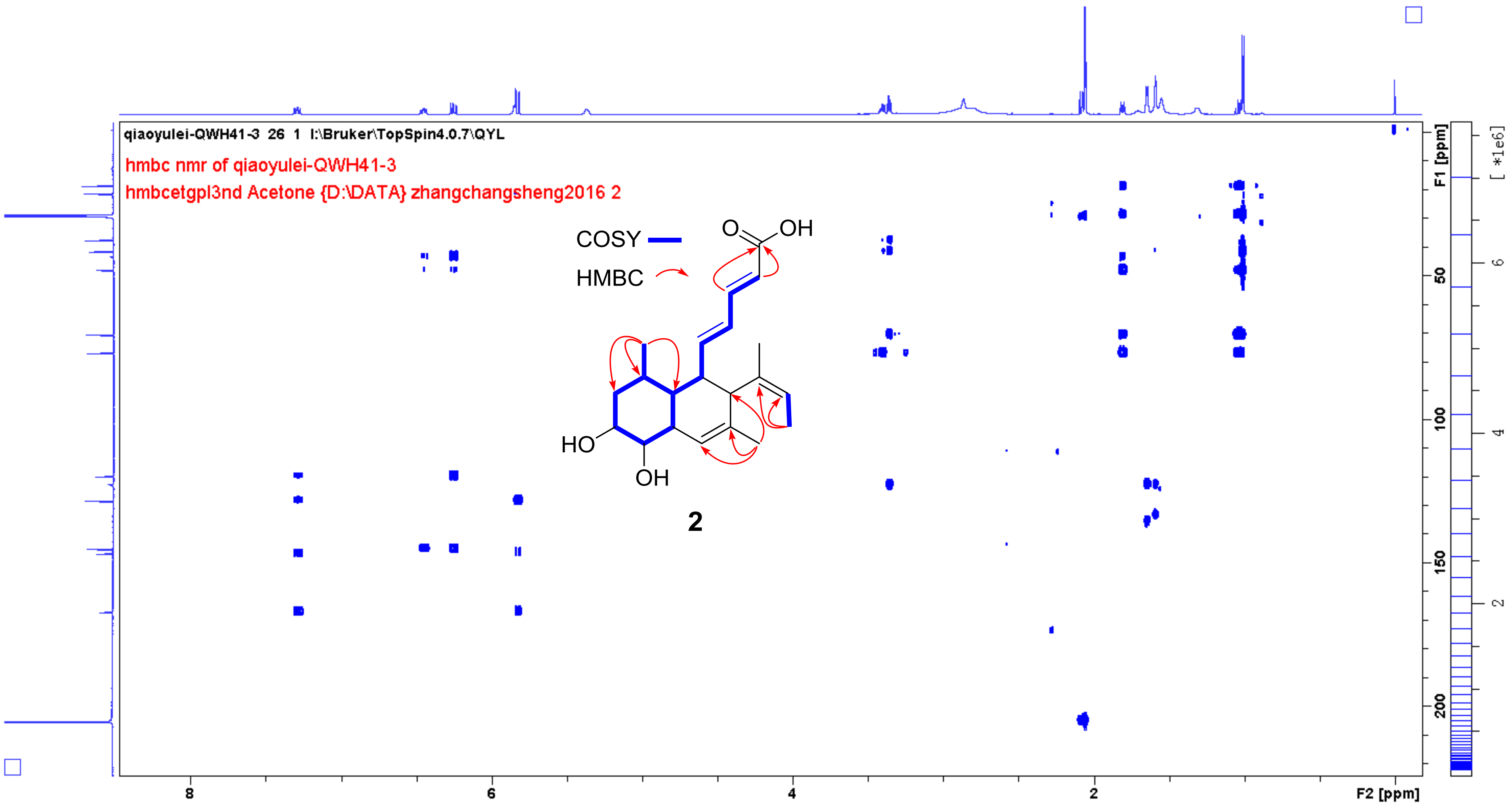

Figure S2. Spectral data for MK8383 B (**2**) (continued).
(I) The HMBC spectrum of MK8383 B (**2**) in Acetone-d_6.

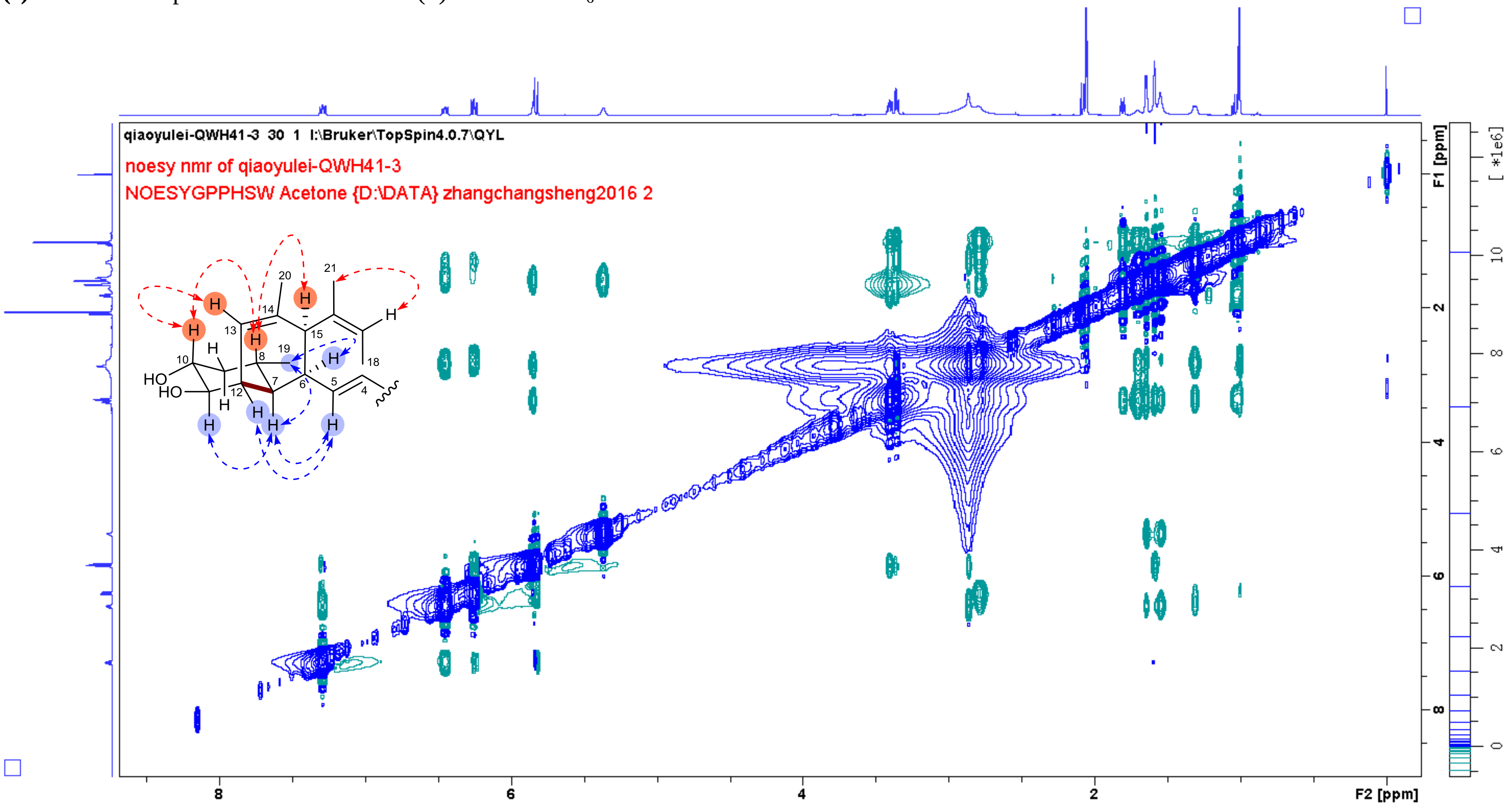

Figure S3. Spectral data for 10-*epi*-MK8383 B (**3**).

(A) HRESIMS

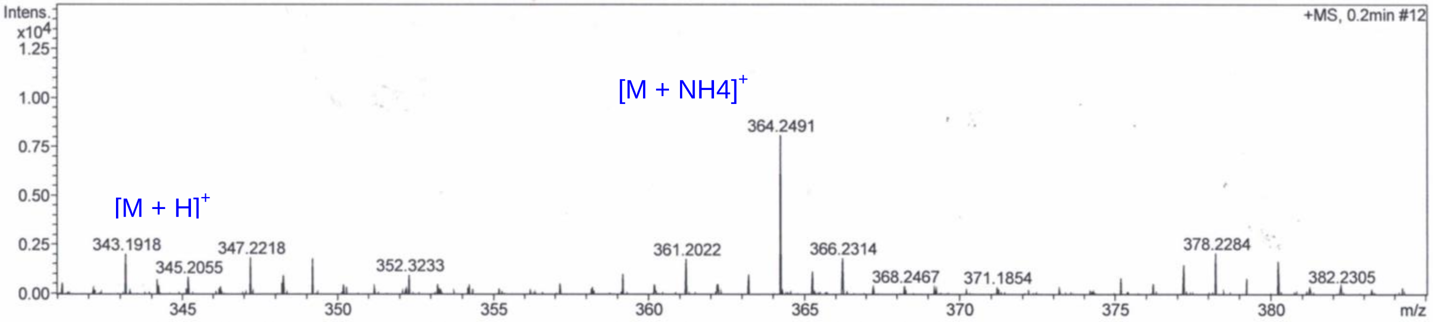

Meas. m/z	#	Ion Formula	Score	m/z	err [ppm]	err [mDa]	mSigma	rdb	e⁻ Conf	N-Rule
347.2218	1	C21H31O4	100.00	347.2217	0.3	0.1	37.2	6.5	even	ok
364.2491	1	C21H34NO4	100.00	364.2482	2.3	0.8	50.5	5.5	even	ok

(**B**) UV

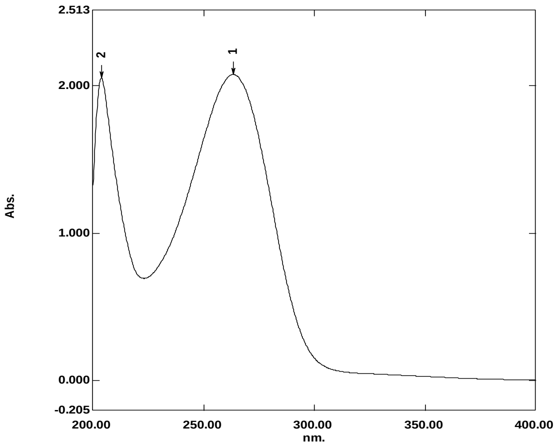

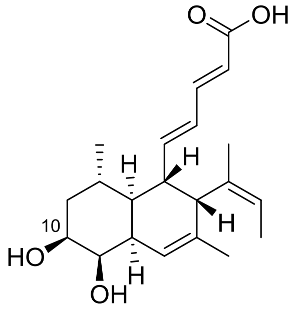

3

Chemical Formula: $C_{21}H_{30}O_4$

Exact Mass: 346.2

(**C**) IR

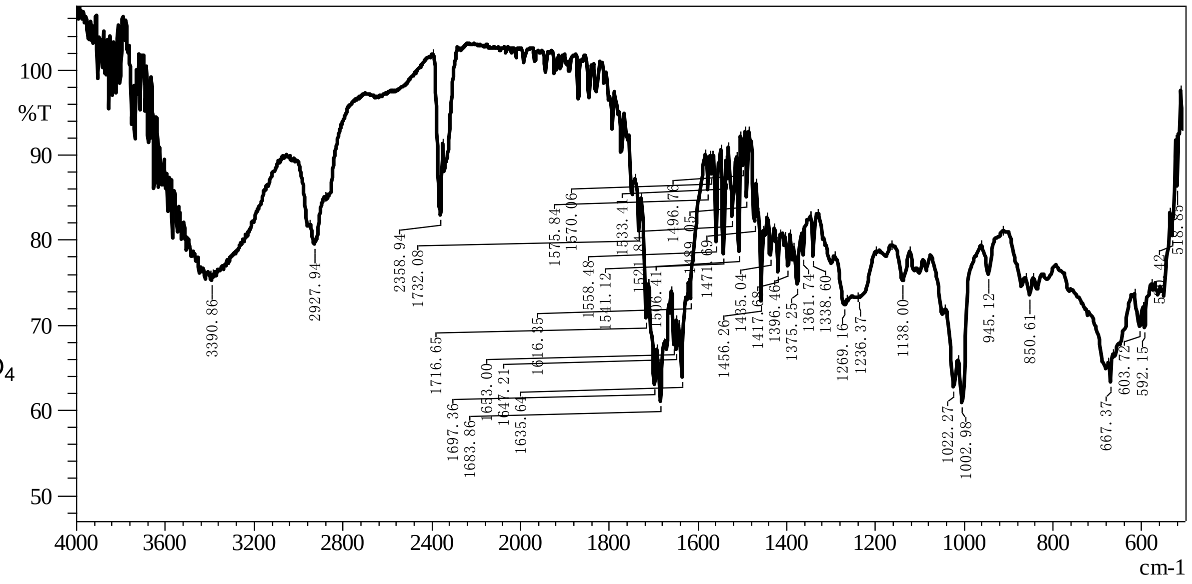

Figure S3. Spectral data for 10-*epi*-MK8383 B (**3**) (continued).

(D) The ^{1}H and enlarged ^{1}H NMR spectrum of 10-*epi*-MK8383 B (**3**) in DMSO-d_6.

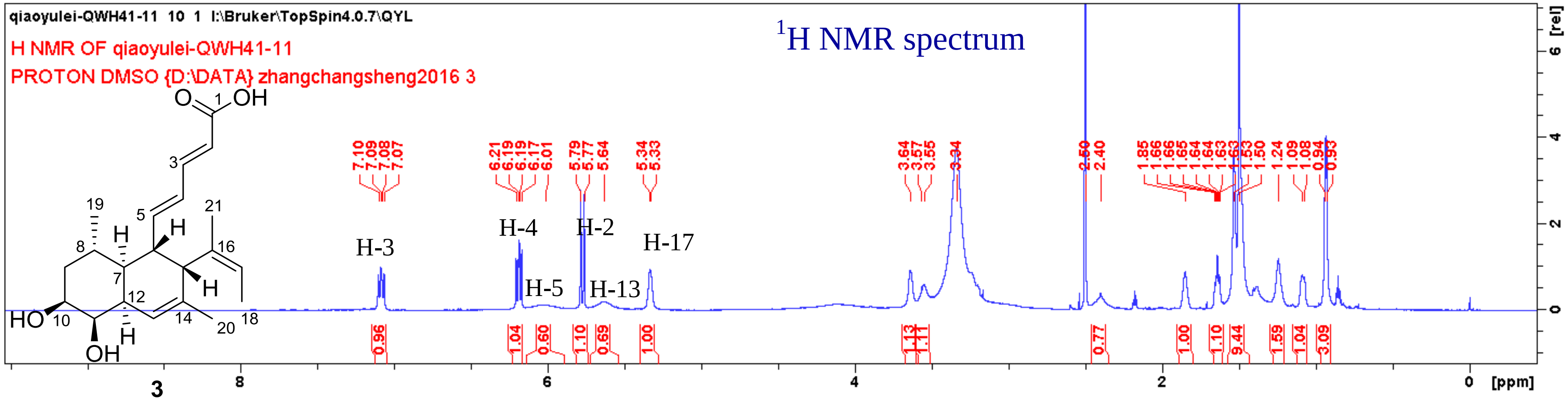

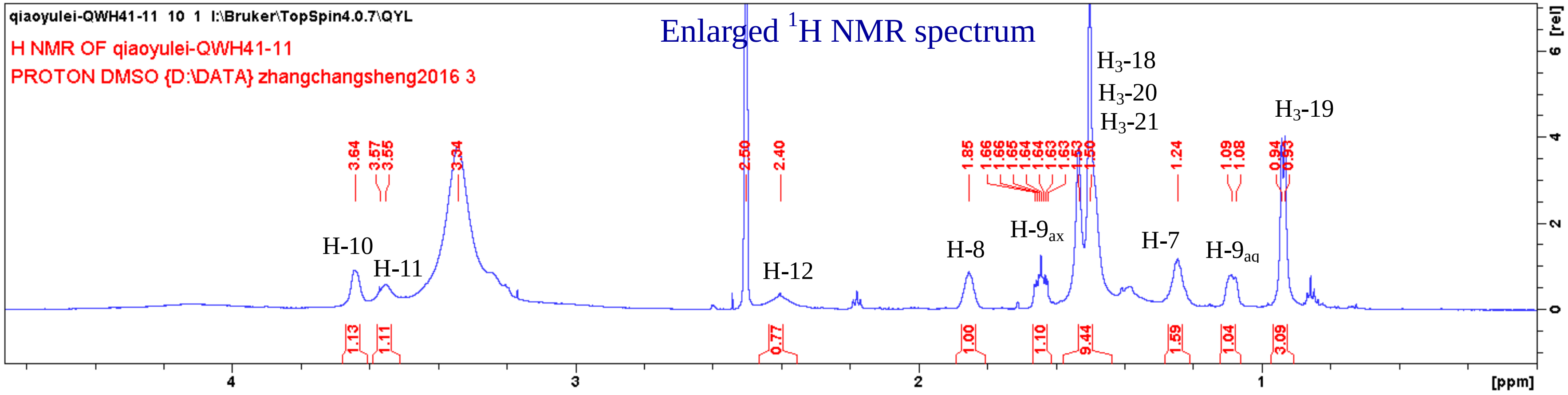

Figure S3. Spectral data for 10-*epi*-MK8383 B (**3**) (continued).

(E) The ^{13}C and enlarged ^{13}C NMR spectrum of 10-*epi*-MK8383 B (**3**) in DMSO-d_6.

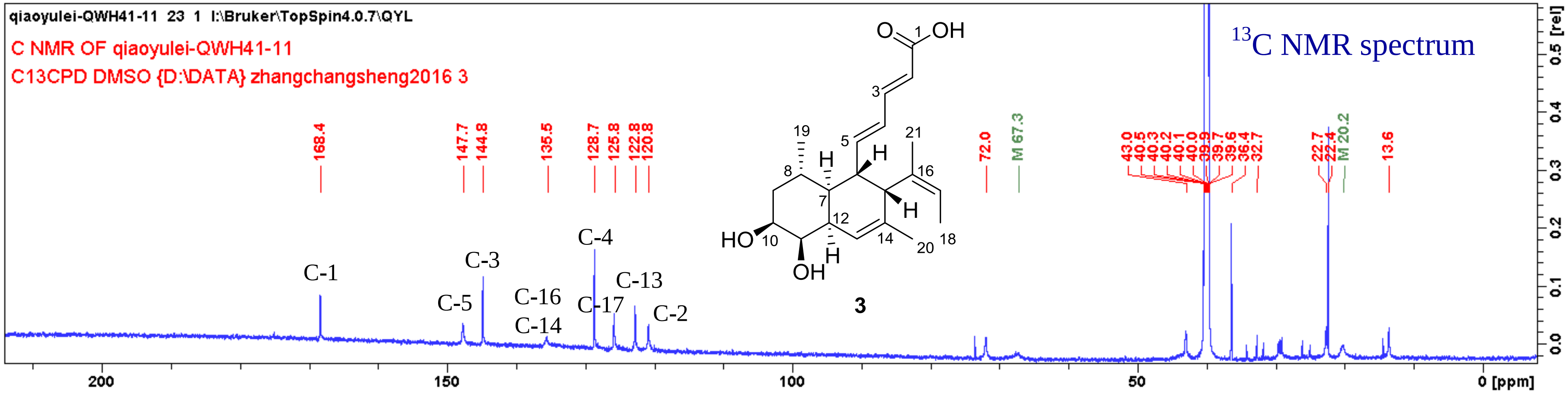

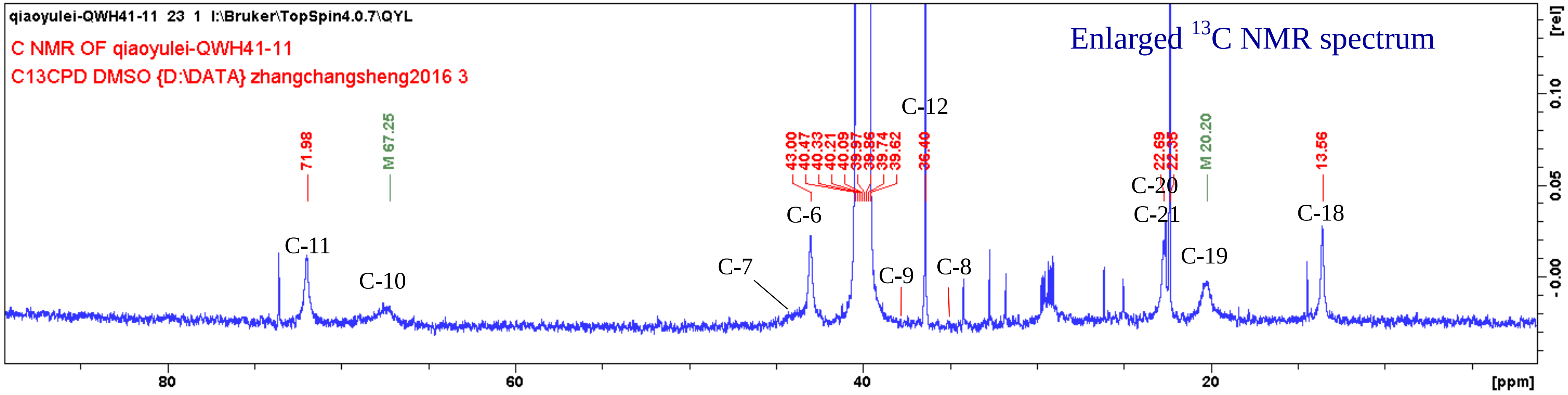

Figure S3. Spectral data for 10-*epi*-MK8383 B (**3**) (continued).
(F) The HSQC spectrum of 10-*epi*-MK8383 B (**3**) in DMSO-d_6.

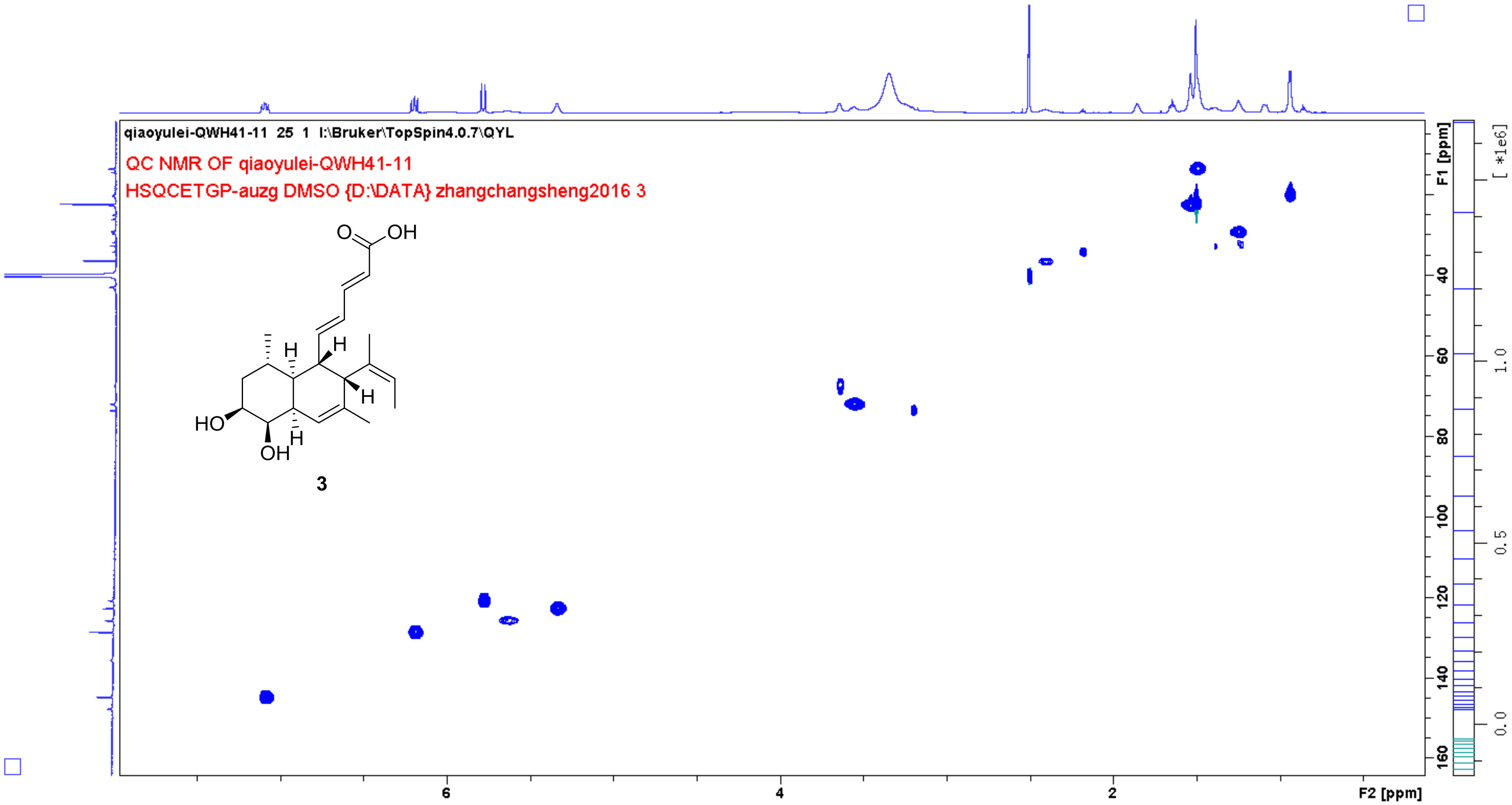

Figure S3. Spectral data for 10-*epi*-MK8383 B (**3**) (continued).

(G) The ^{1}H-^{1}H COSY spectrum of 10-*epi*-MK8383 B (**3**) in DMSO-d_6.

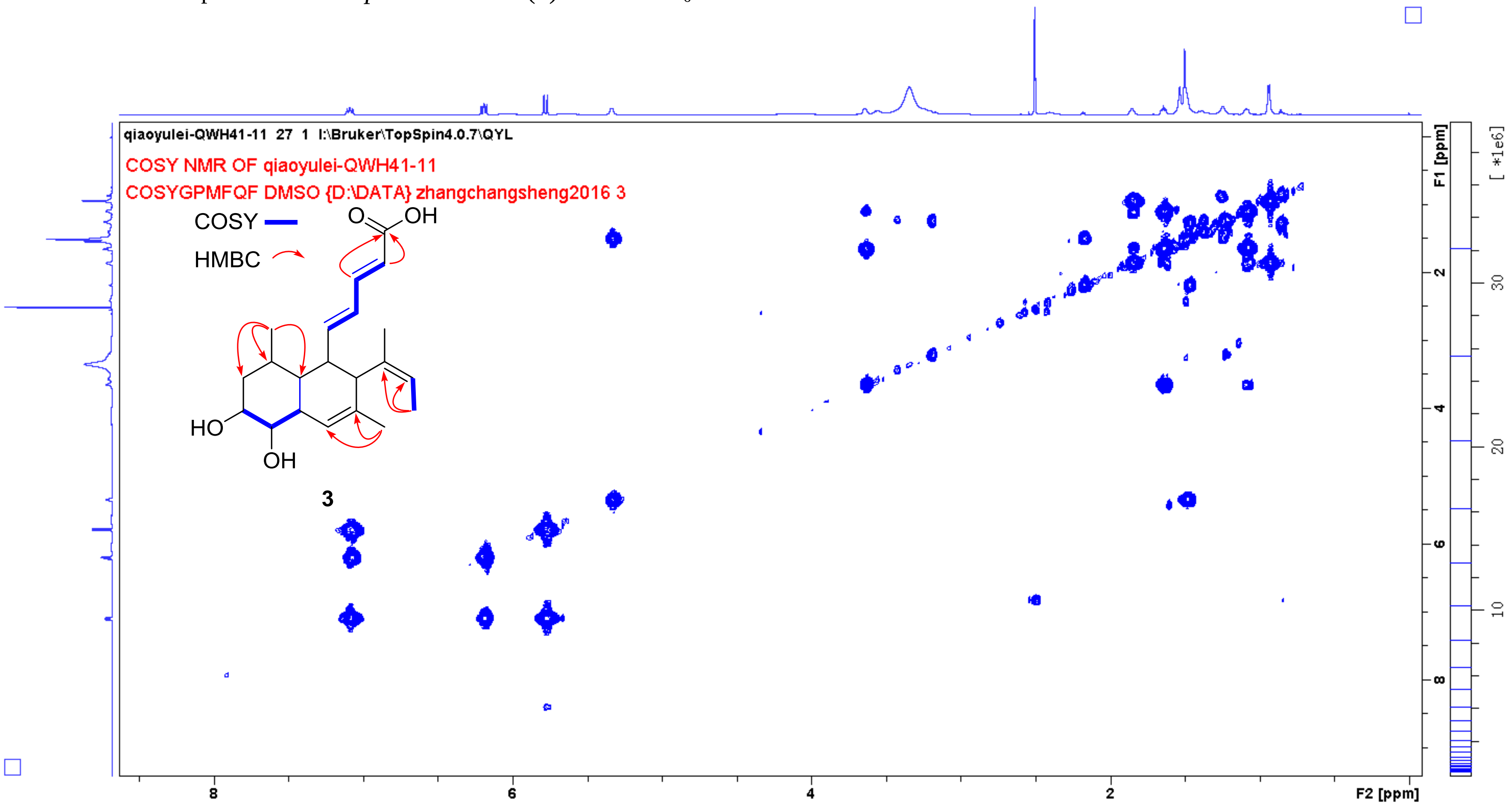

Figure S3. Spectral data for 10-*epi*-MK8383 B (**3**) (continued).

(H) The HMBC spectrum of 10-*epi*-MK8383 B (**3**) in DMSO-d_6..

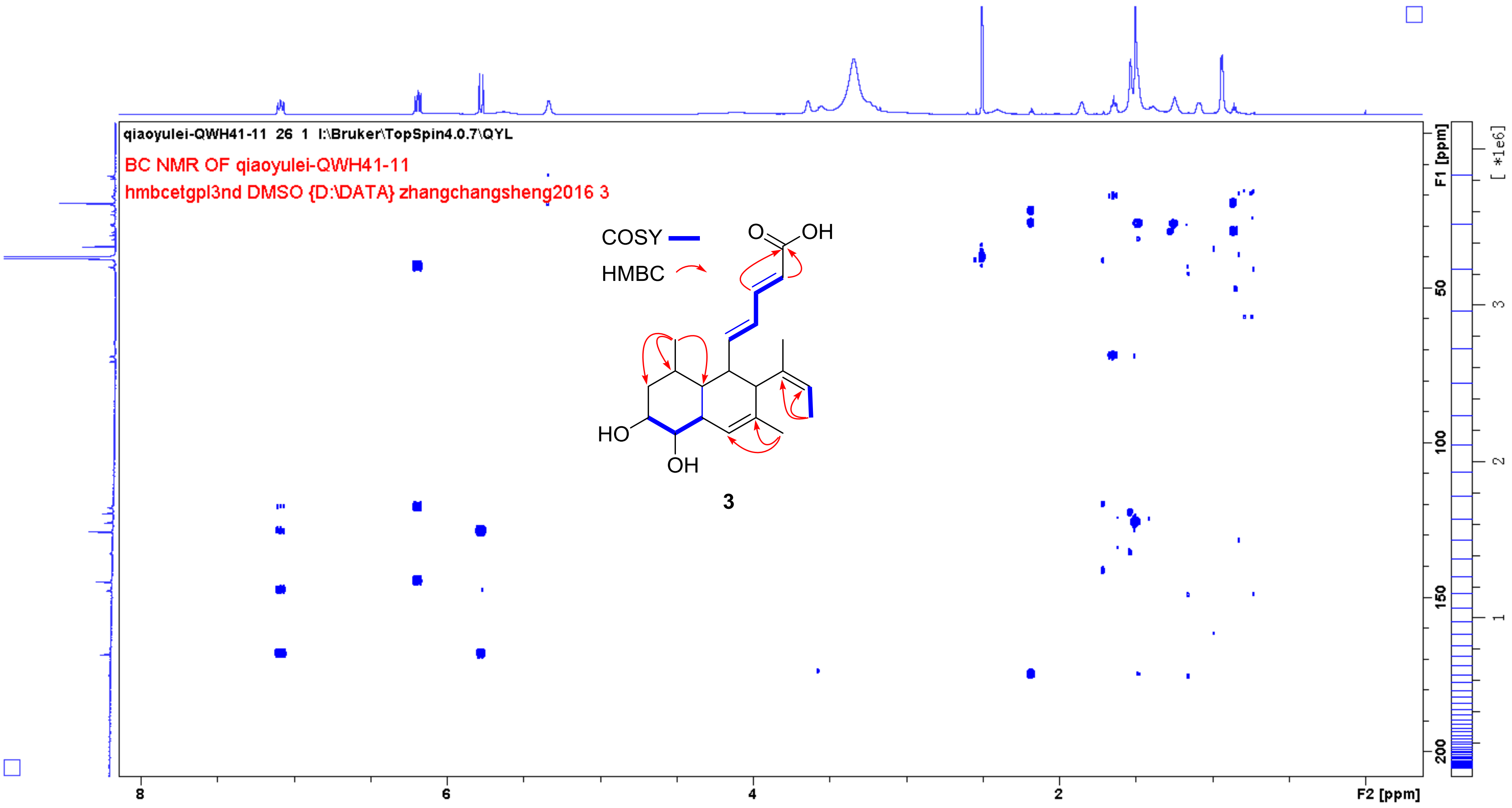

Figure S3. Spectral data for 10-*epi*-MK8383 B (**3**) (continued).

(I) The NOESY spectrum of 10-*epi*-MK8383 B (**3**) in DMSO-d_6.

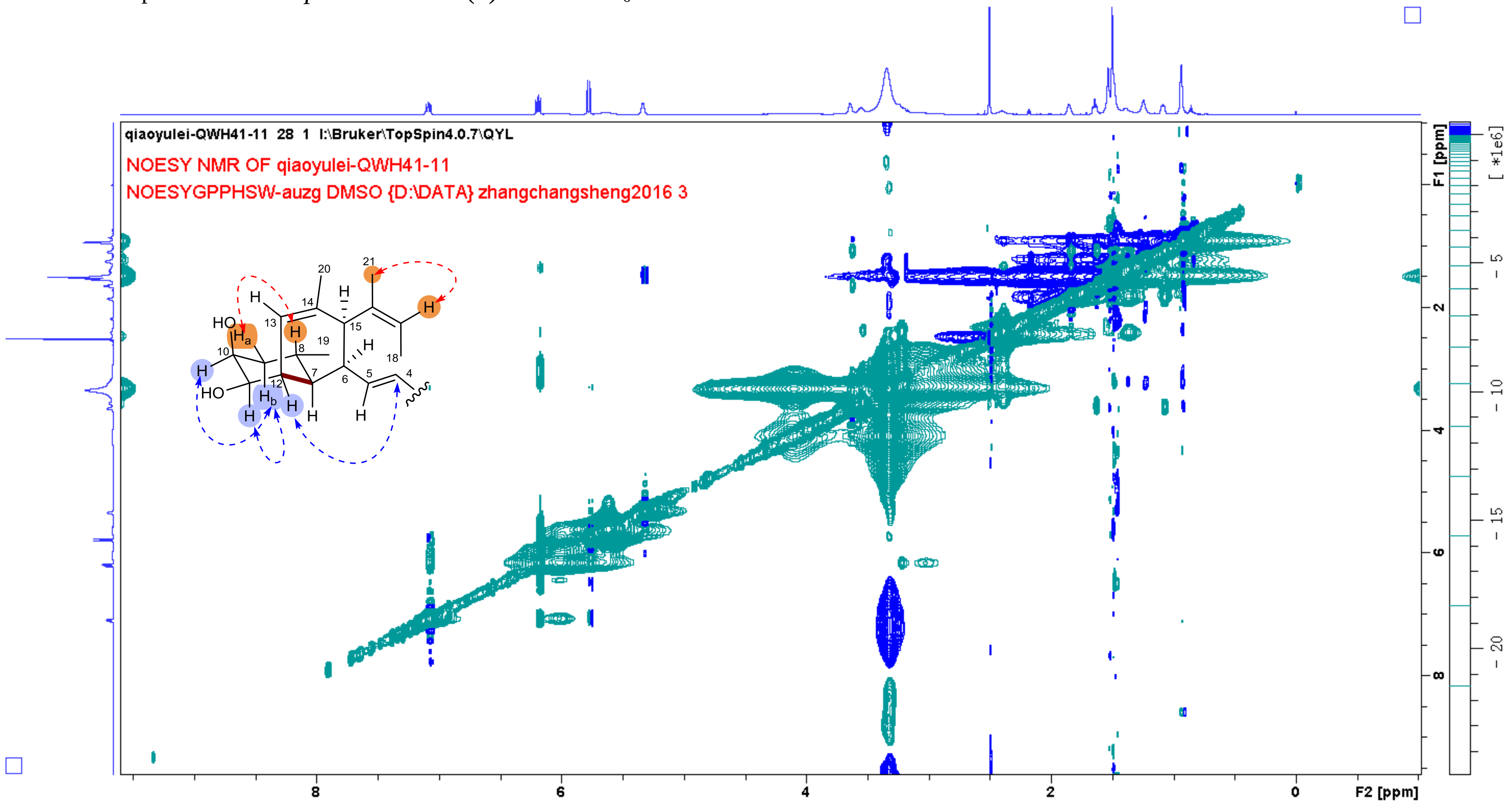

Figure S4. Spectral data for MK8383 C (**4**).

(A) HRESIMS

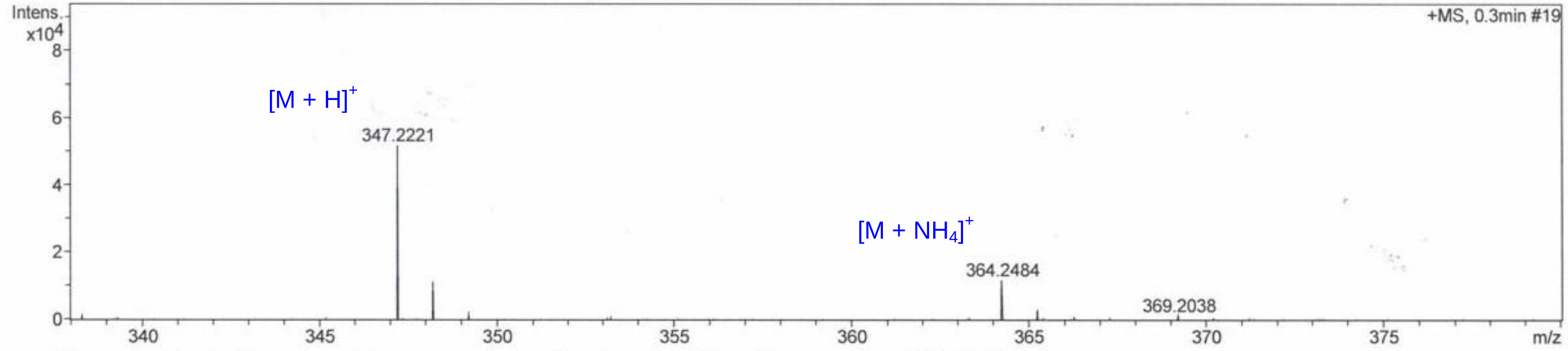

Meas. m/z	#	Ion Formula	Score	m/z	err [ppm]	err [mDa]	mSigma	rdb	e⁻ Conf	N-Rule
347.2221	1	C21H31O4	100.00	347.2217	-1.3	-0.5	8.8	6.5	even	ok
364.2484	1	C21H34NO4	100.00	364.2482	0.5	0.2	17.9	5.5	even	ok

(**B**) UV

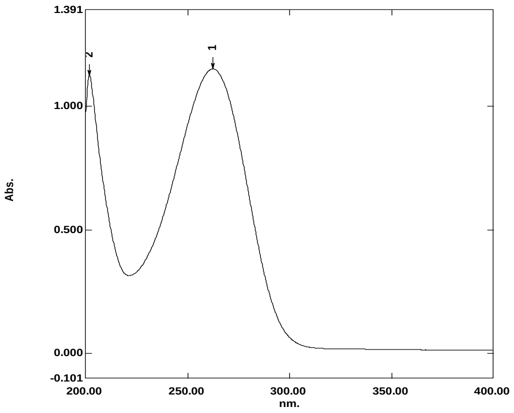

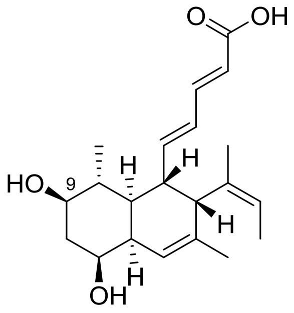

4

Chemical Formula: $C_{21}H_{30}O_4$

Exact Mass: 346.2

(**C**) IR

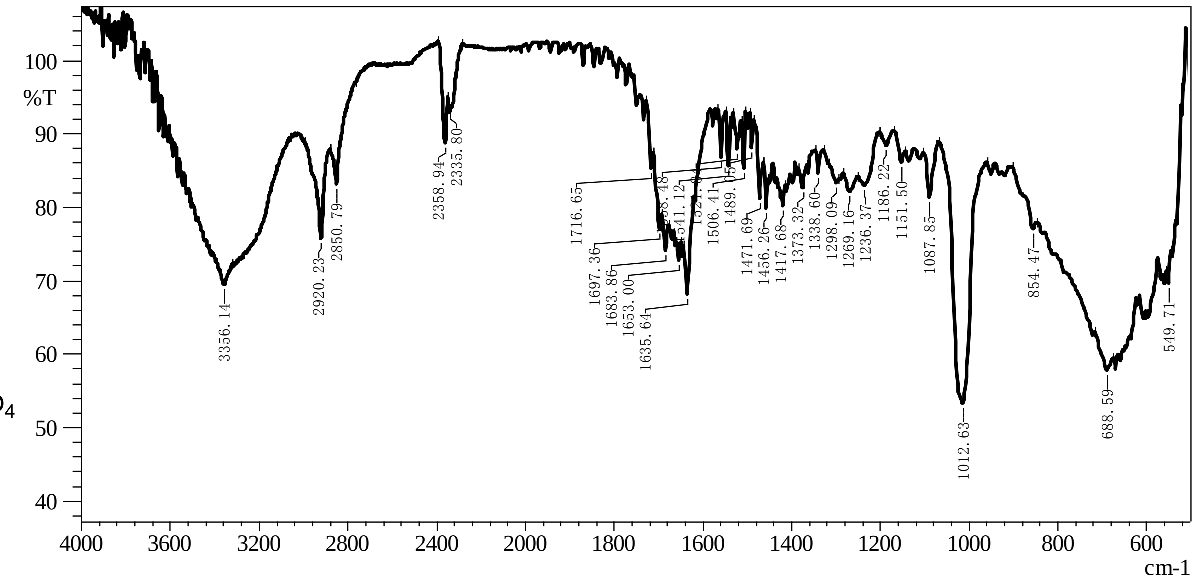

Figure S4. Spectral data for MK8383 **C** (**4**) (continued).
(D) The ^{1}H and enlarged ^{1}H NMR spectrum of MK8383 **C** (**4**) in Acetone-d_6.

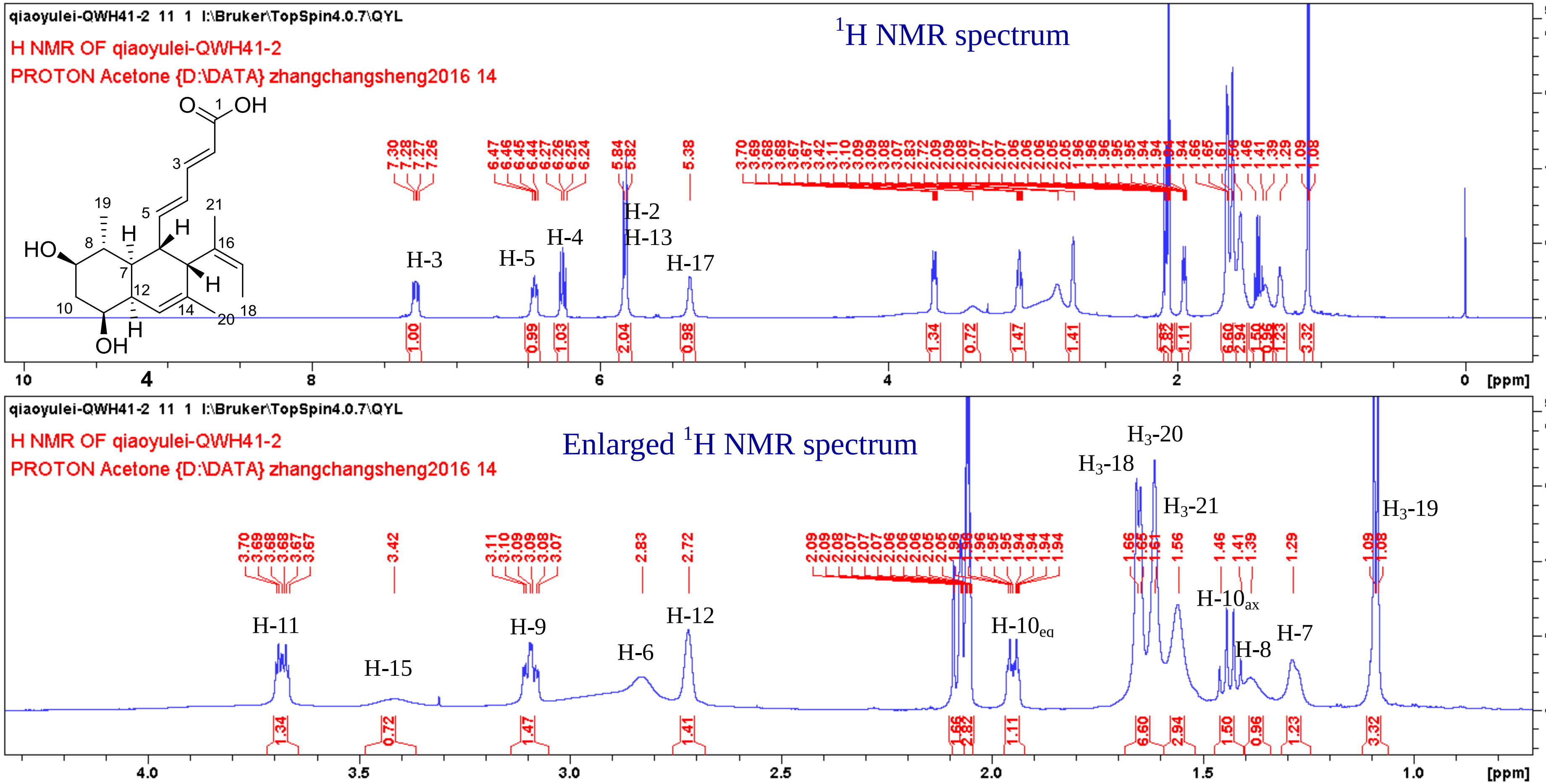

Figure S4. Spectral data for MK8383 **C** (**4**) (continued).

(E) The ^{13}C and enlarged ^{13}C NMR spectrum of MK8383 **C** (**4**) in Acetone-d_6

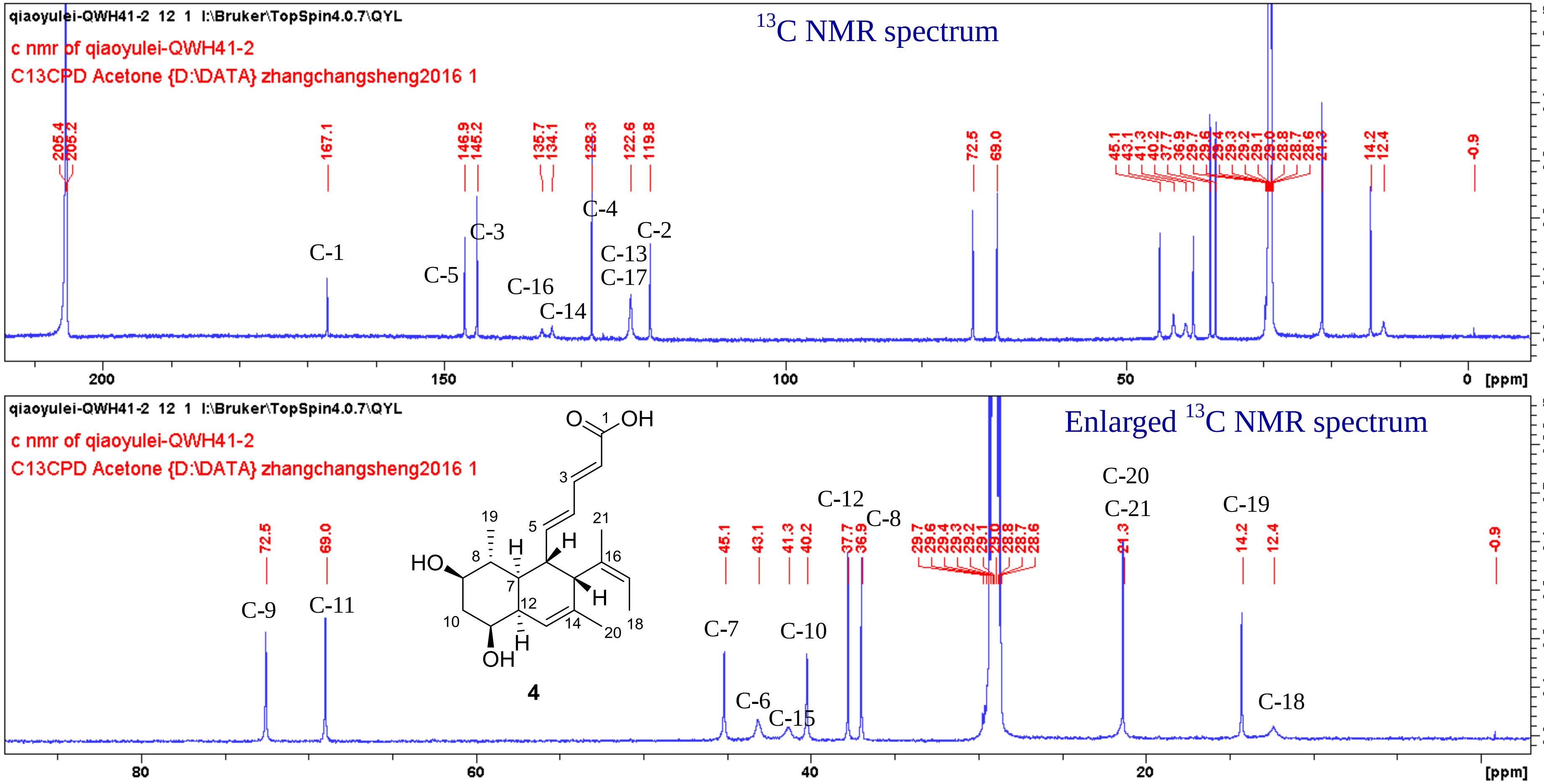

Figure S4. Spectral data for MK8383 **C** (**4**).
(F) The HSQC spectrum of MK8383 **C** (**4**) in Acetone-d_6.

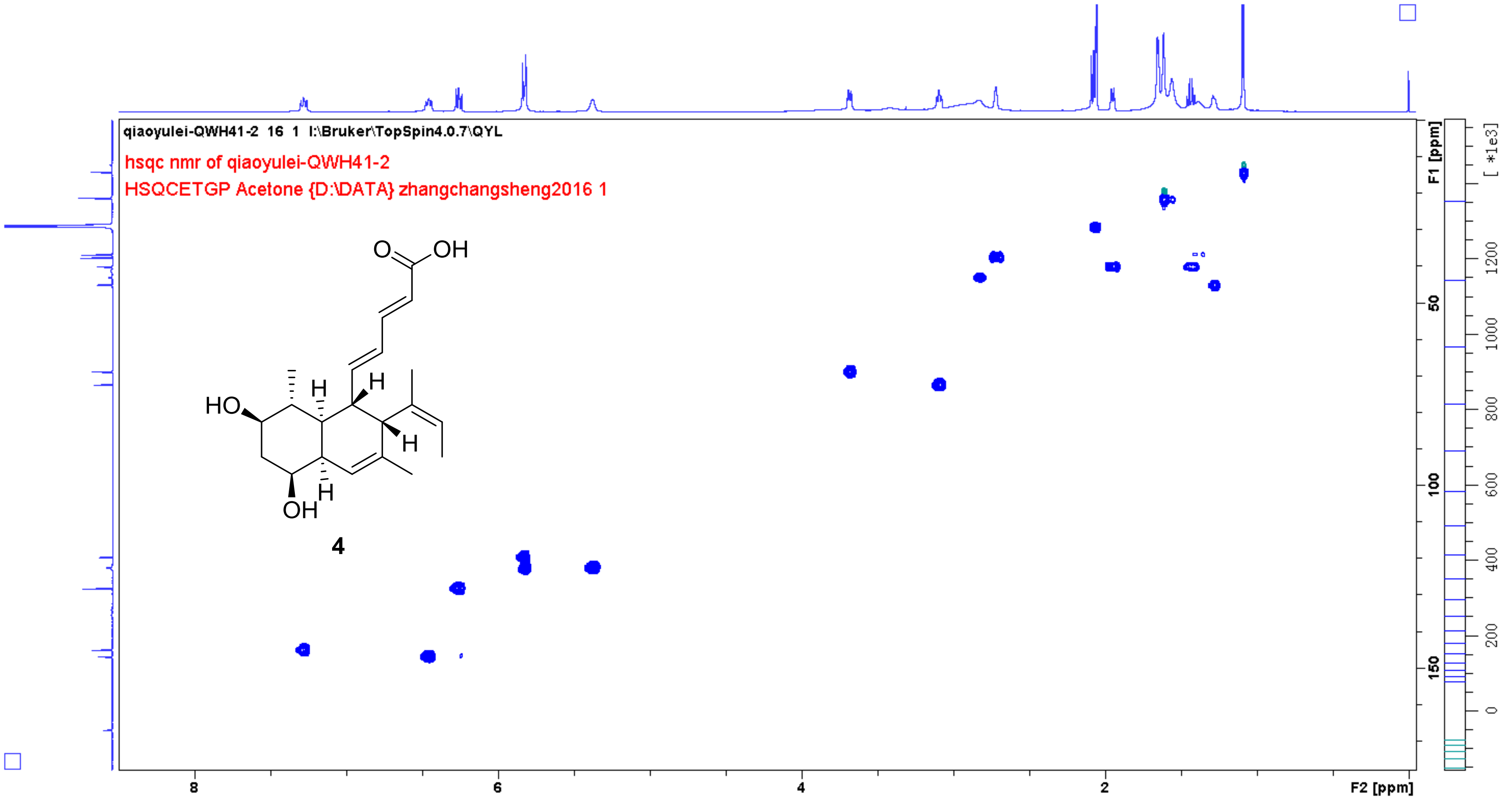

Figure S4. Spectral data for MK8383 **C** (**4**) (continued).
(G) The ^{1}H-^{1}H COSY spectrum of MK8383 **C** (**4**) in Acetone-d_6.

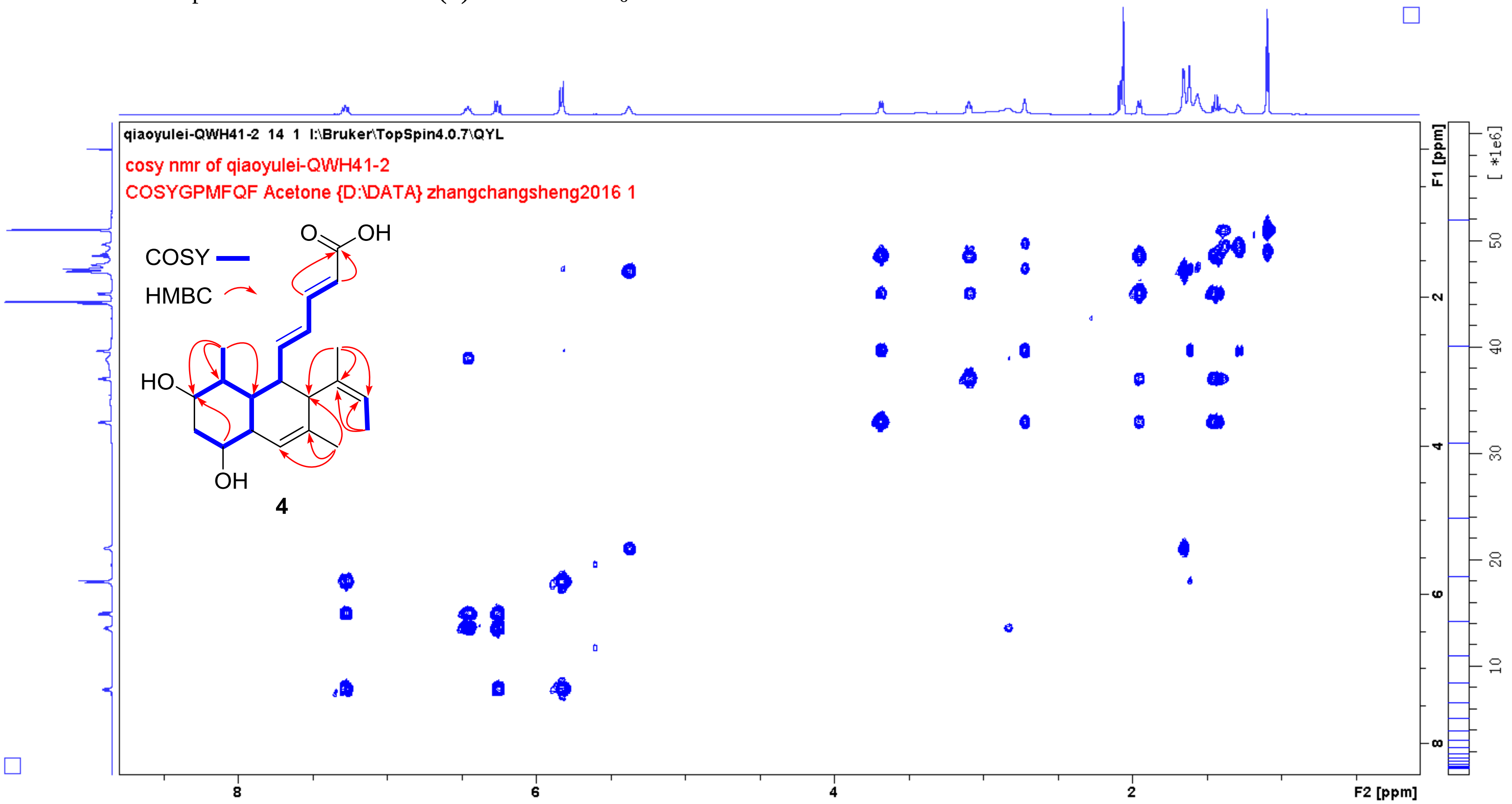

Figure S4. Spectral data for MK8383 **C** (**4**).

(H) The HMBC spectrum of MK8383 **C** (**4**) in Acetone-d_6.

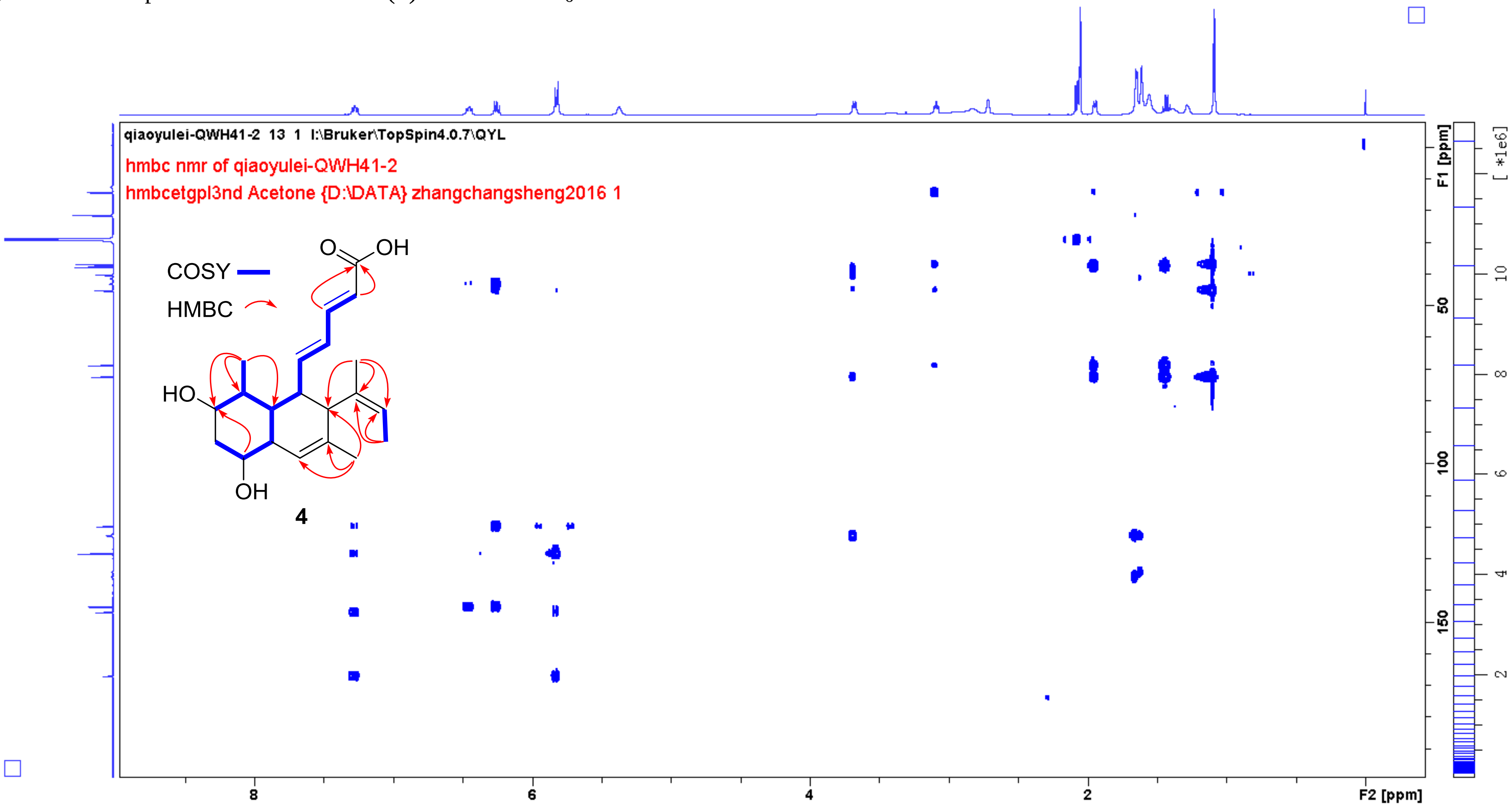

Figure S4. Spectral data for MK8383 **C** (**4**).

(I) The NOESY spectrum of MK8383 **C** (**4**) in Acetone-d_6.

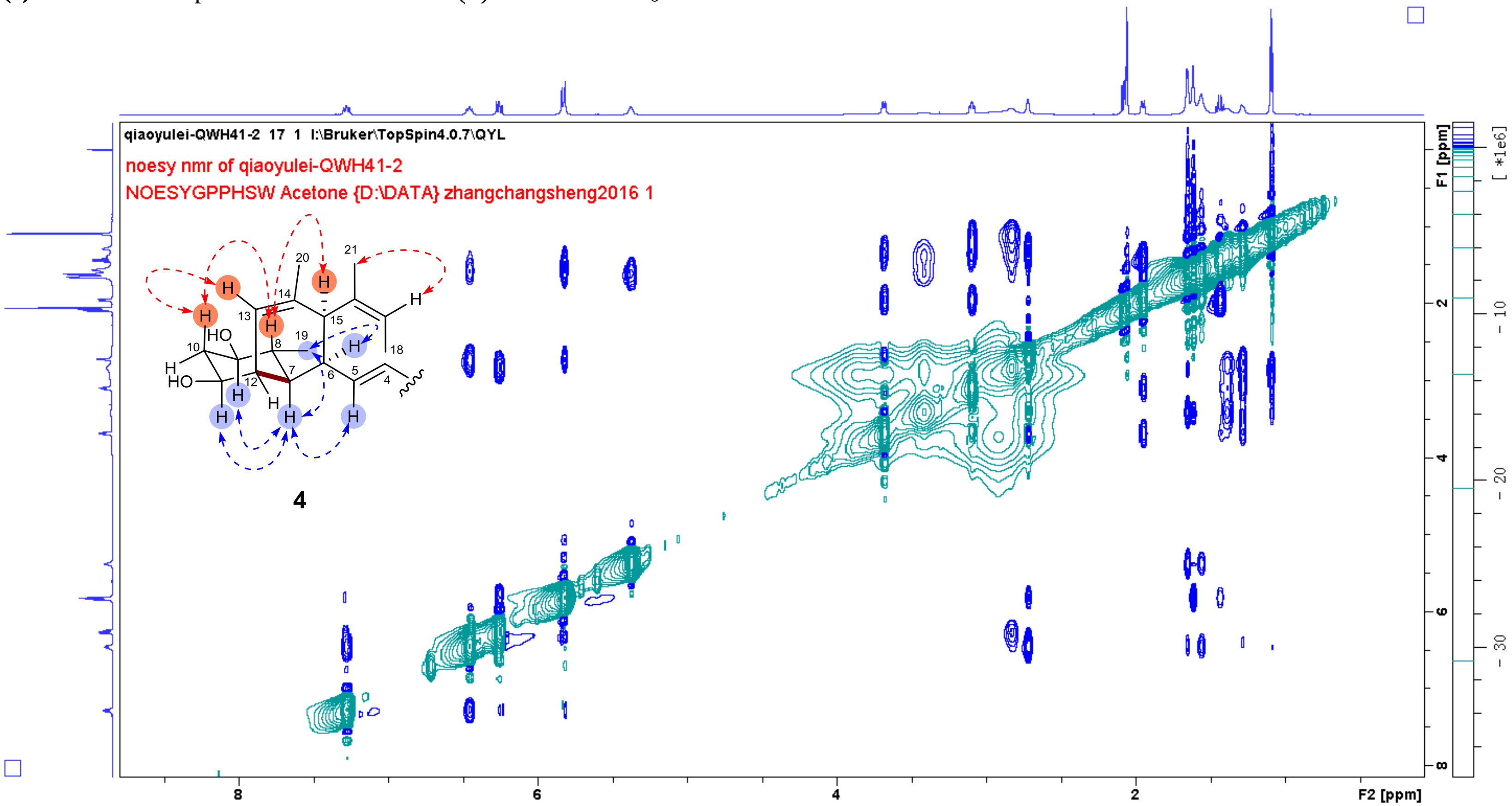

Figure S5. Spectral data for 9-*epi*-MK8383 C (**5**).

(**A**) HRESIMS

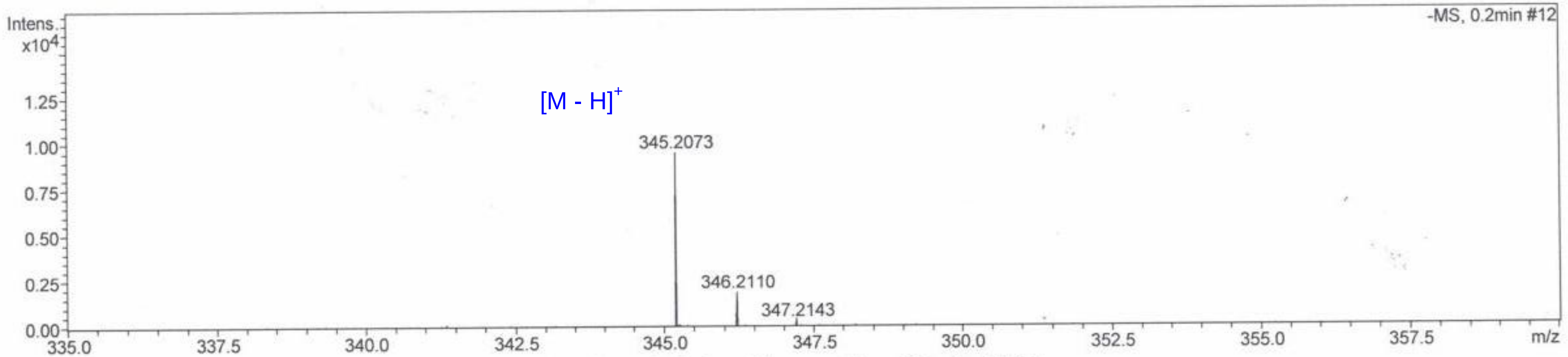

Meas. m/z	#	Ion Formula	Score	m/z	err [ppm]	err [mDa]	mSigma	rdb	e⁻ Conf	N-Rule
345.2073	1	C21H29O4	100.00	345.2071	0.6	0.2	18.4	7.5	even	ok

(**B**) UV

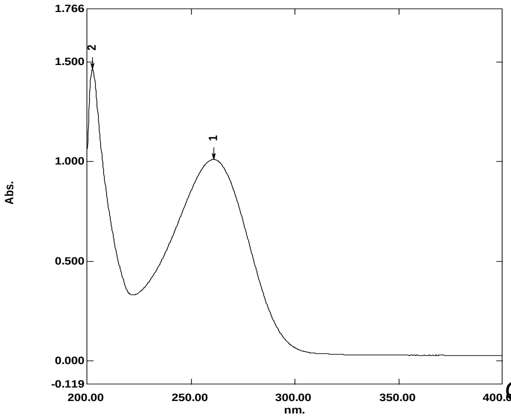

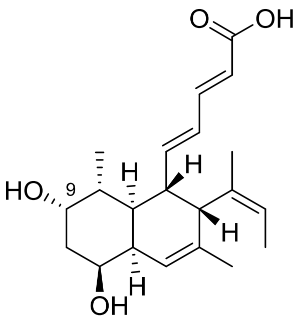

5

Chemical Formula: $C_{21}H_{30}O_4$

Exact Mass: 346.2

(**C**) IR

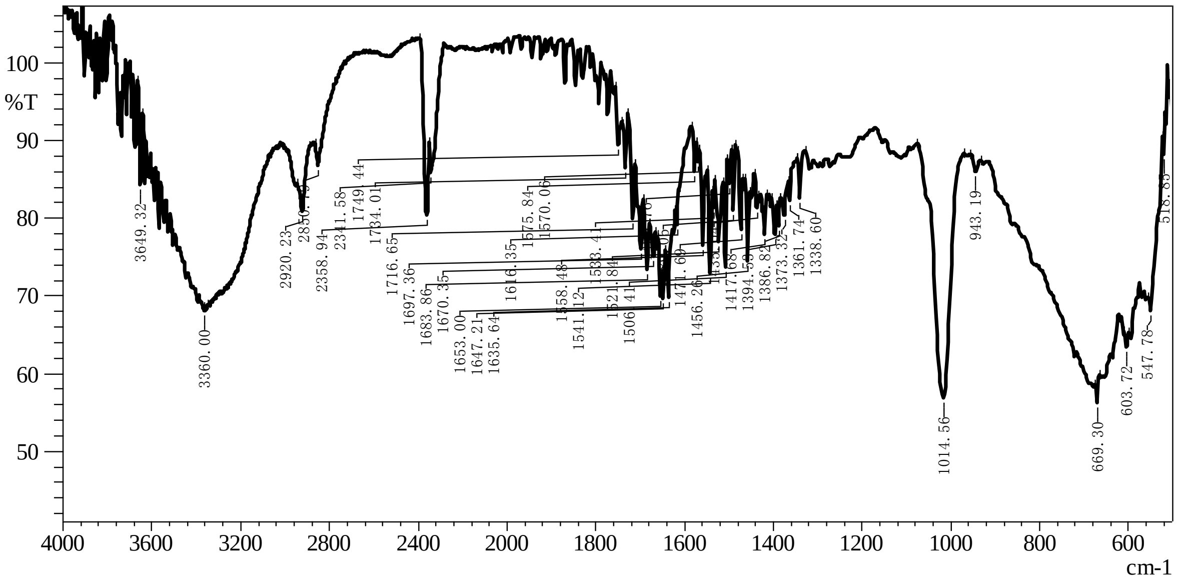

Figure S5. Spectral data for 9-*epi*-MK8383 C (**5**) (continued).
(D) The ^{1}H and enlarged ^{1}H NMR spectrum of 9-*epi*-MK8383 C (**5**) in Acetone-d_6.

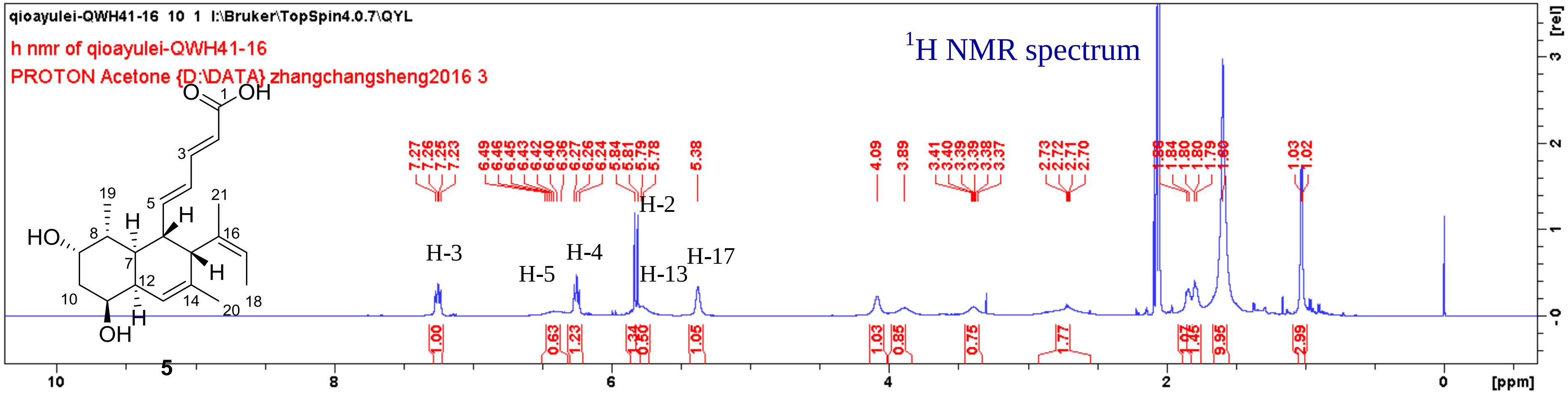

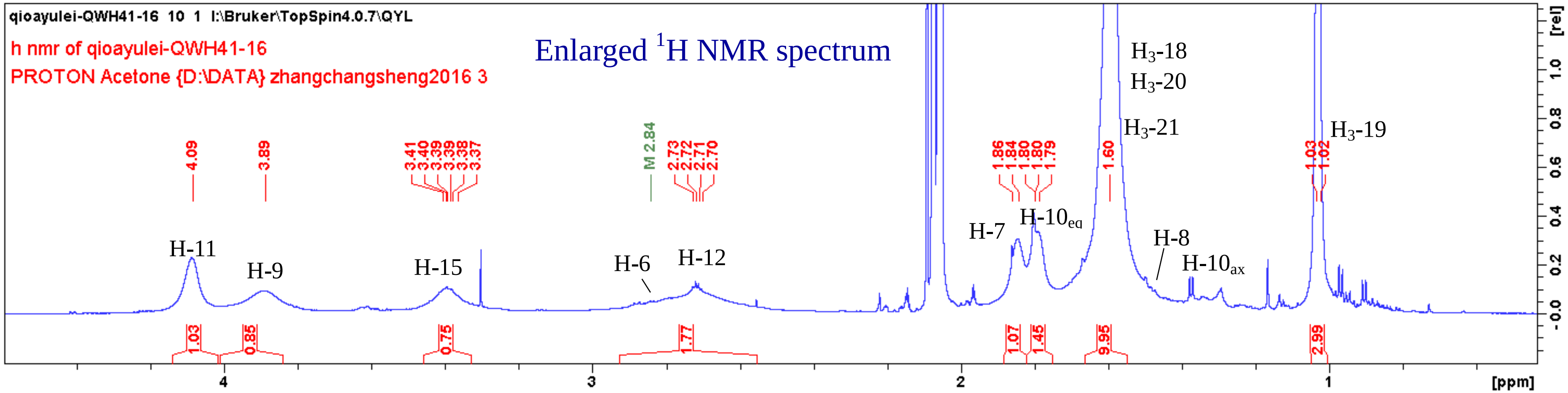

Figure S5. Spectral data for 9-*epi*-MK8383 C (continued).

(E) The ^{13}C and enlarged ^{13}C NMR spectra of 9-*epi*-MK8383 C (**5**) in Acetone-d_6.

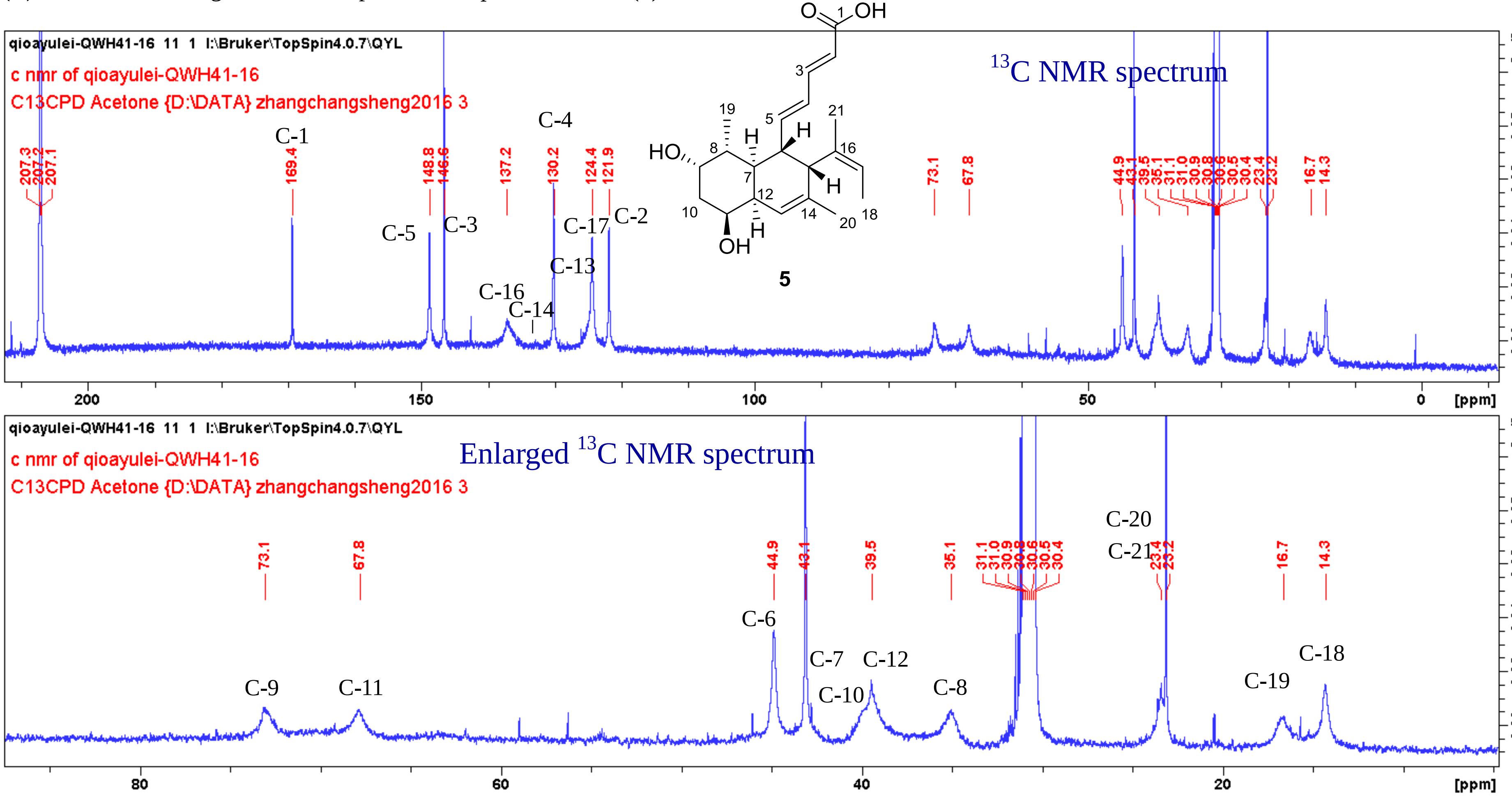

Figure S5. Spectral data for 9-*epi*-MK8383 C (**5**) (continued).
(**F**) The HSQC spectrum of 9-*epi*-MK8383 C (**5**) in Acetone-d_6.

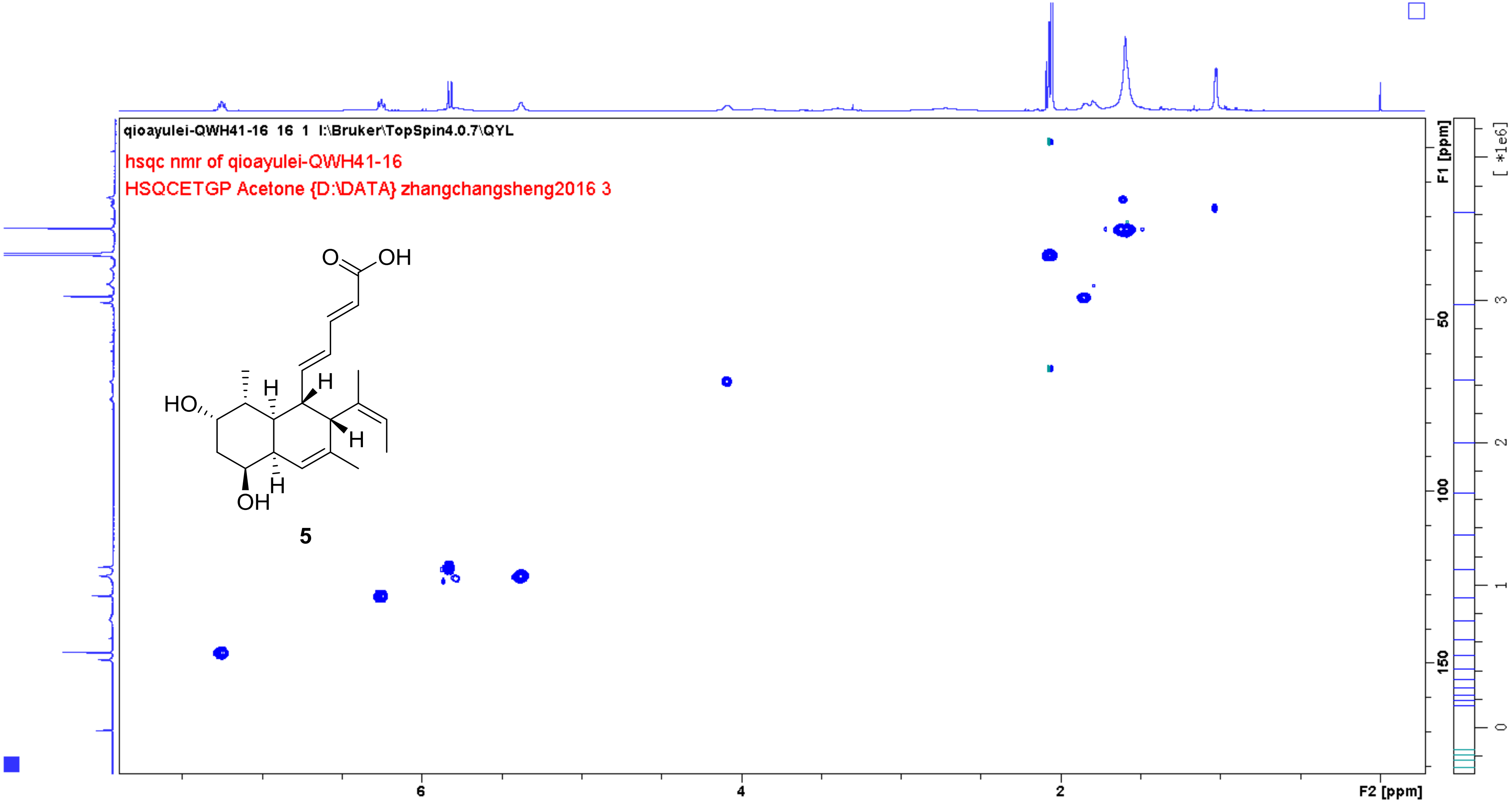

Figure S5. Spectral data for 9-*epi*-MK8383 C (**5**) (continued).

(G) The ^{1}H-^{1}H COSY spectrum of 9-*epi*-MK8383 C (**5**) in Acetone-d_6.

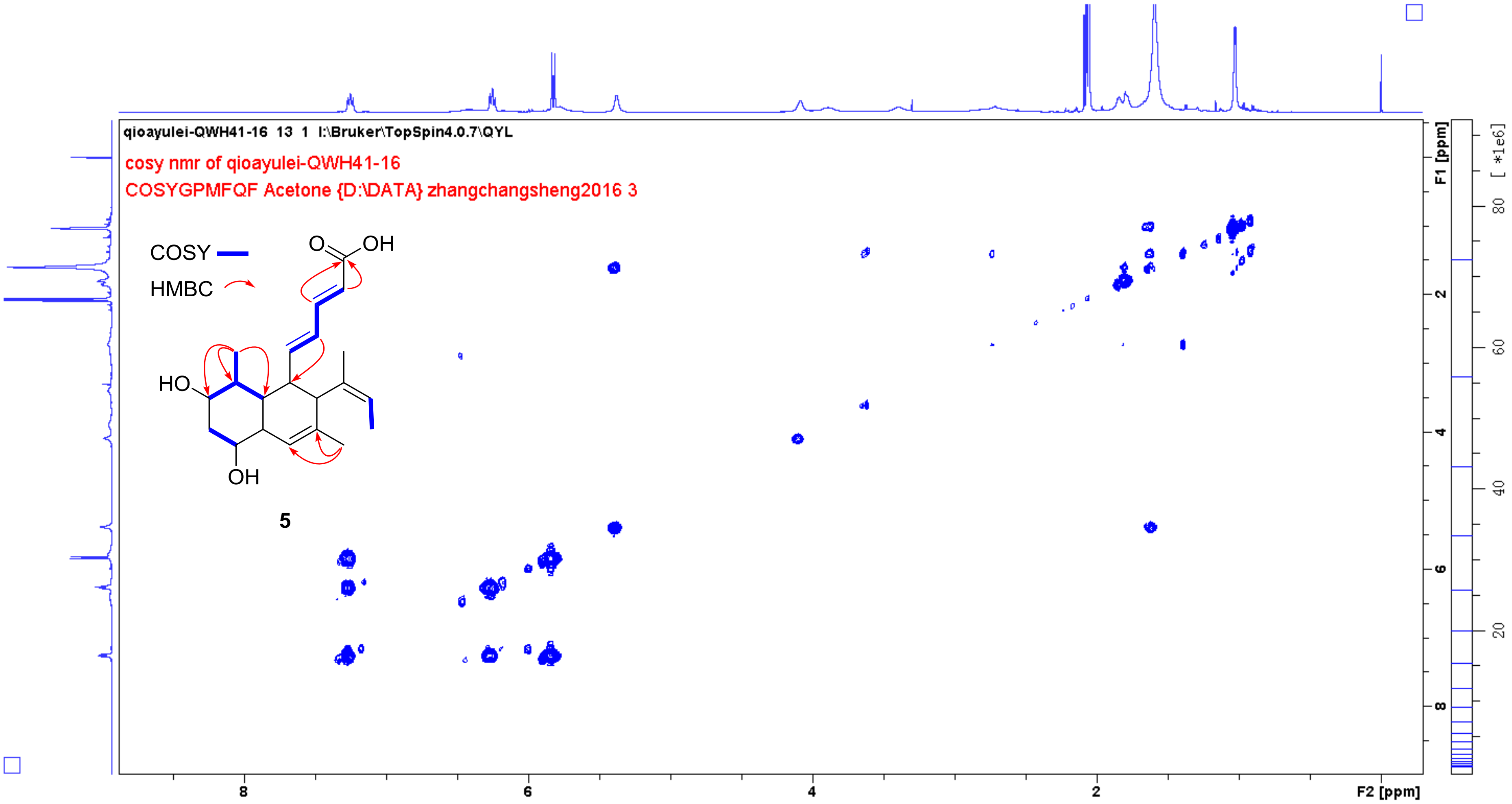

Figure S5. Spectral data for 9-*epi*-MK8383 C (**5**) (continued).

(H) The HMBC spectrum of 9-*epi*-MK8383 C (**5**) in Acetone-d_6.

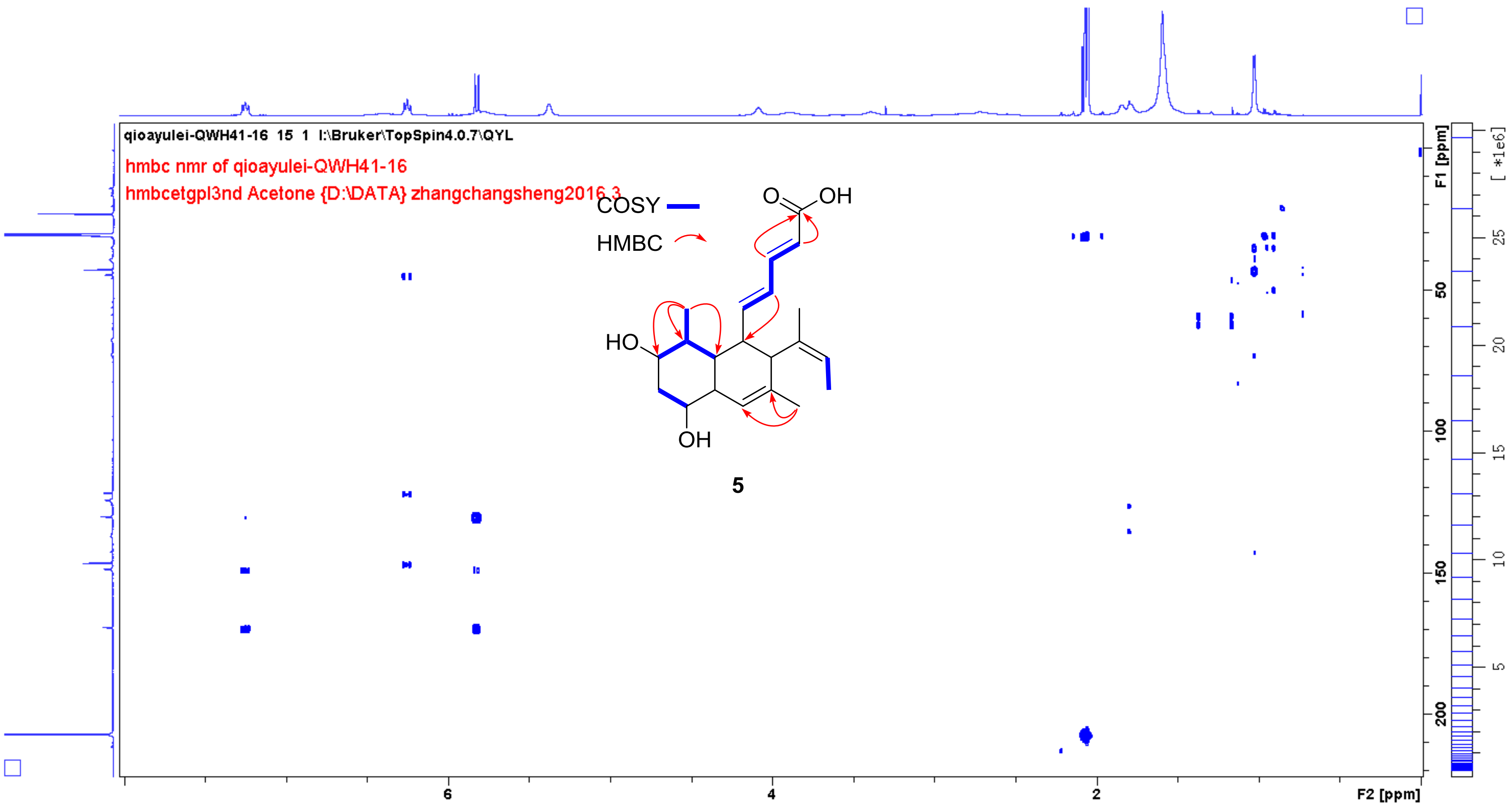

Figure S5. Spectral data for 9-*epi*-MK8383 C (**5**) (continued).
(I) The NOESY spectrum of 9-*epi*-MK8383 C (**5**) in Acetone-d_6.

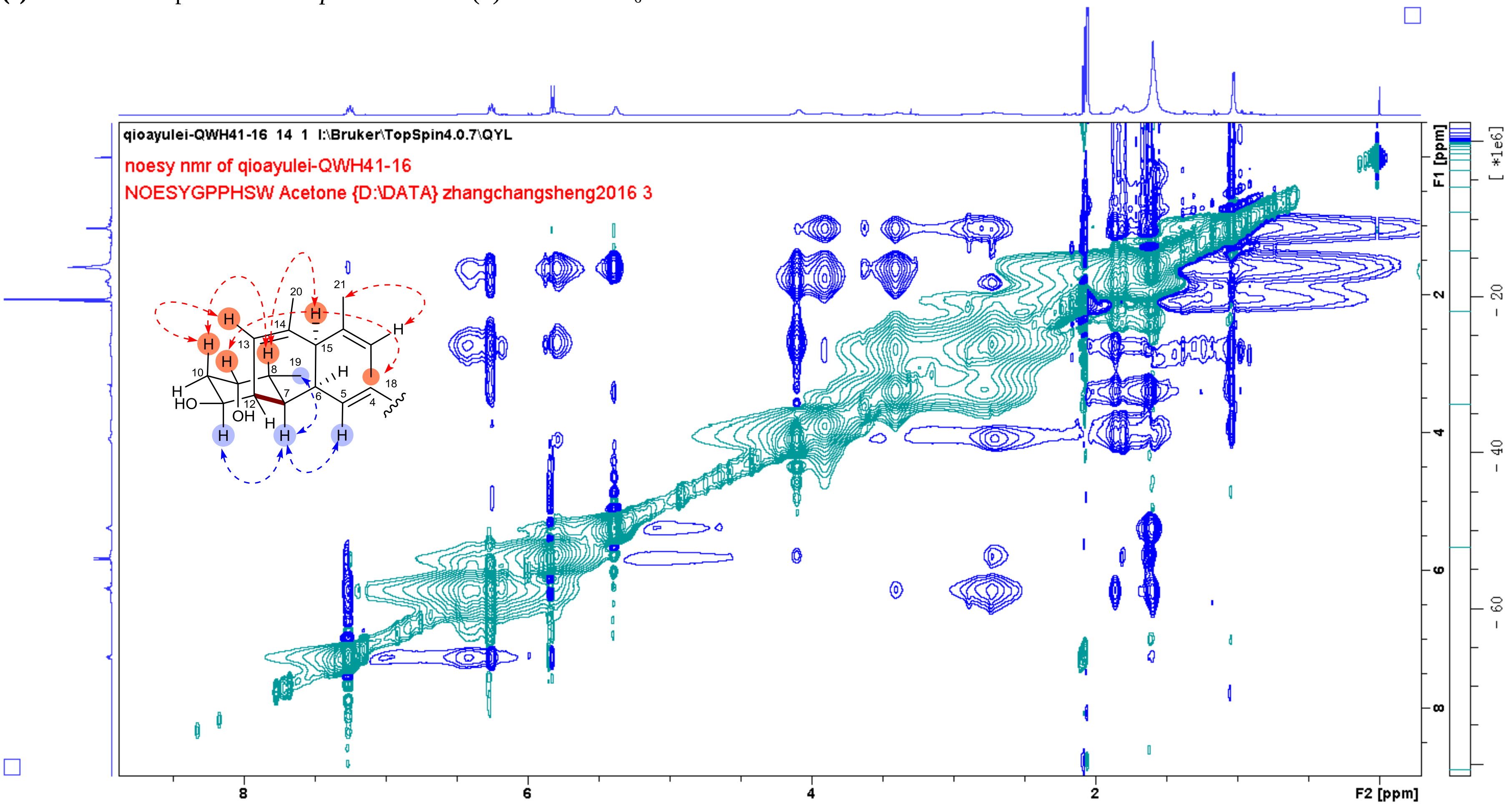

Figure S6. Spectral data for MK8383 D (**6**).

(**A**) HRESIMS

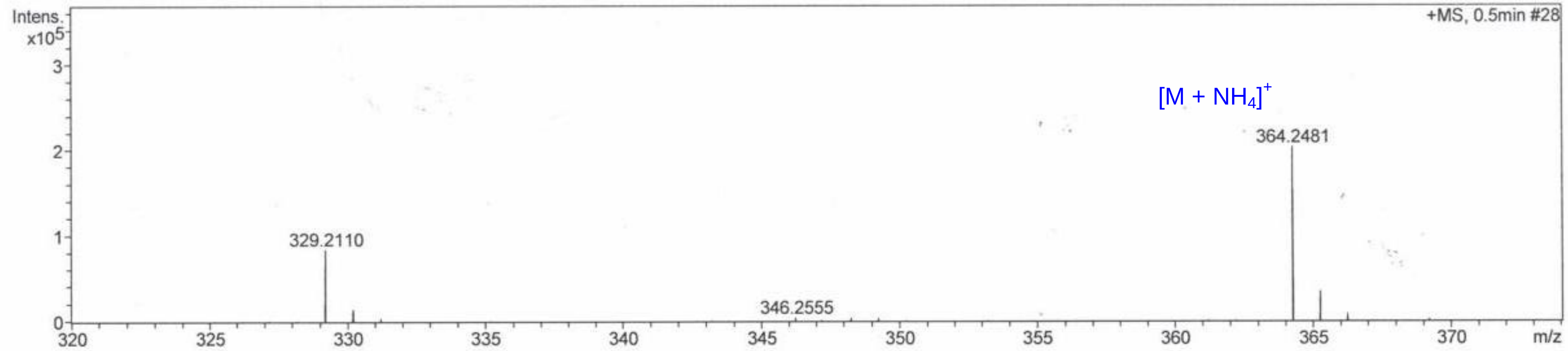

Meas. m/z	#	Ion Formula	Score	m/z	err [ppm]	err [mDa]	mSigma	rdb	e⁻ Conf	N-Rule
329.2110	1	C21H29O3	100.00	329.2111	0.3	0.1	33.0	7.5	even	ok
347.2249	1	C21H31O4	100.00	347.2217	9.2	3.2	151.3	6.5	even	ok
364.2481	1	C21H34NO4	100.00	364.2482	0.3	0.1	32.0	5.5	even	ok

(**B**) UV

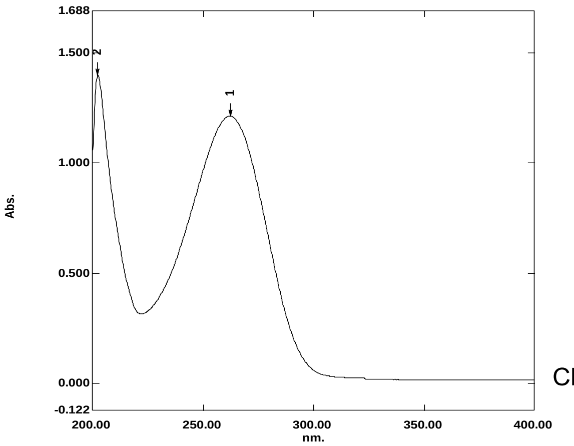

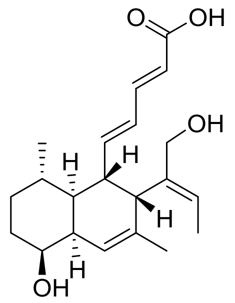

6

Chemical Formula: $C_{21}H_{30}O_4$

Exact Mass: 346.2

(**C**) IR

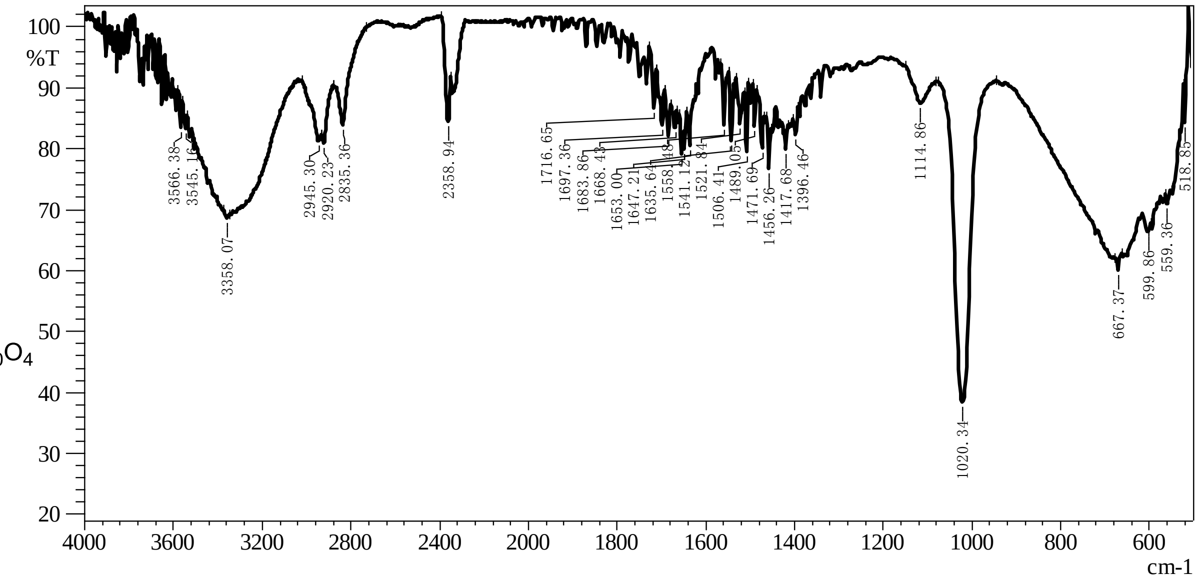

Figure S6. Spectral data for MK8383 D (**6**) (continued).

(D) The ^{1}H and enlarged ^{1}H NMR spectrum of MK8383 D (**6**) in Acetone-d_6.

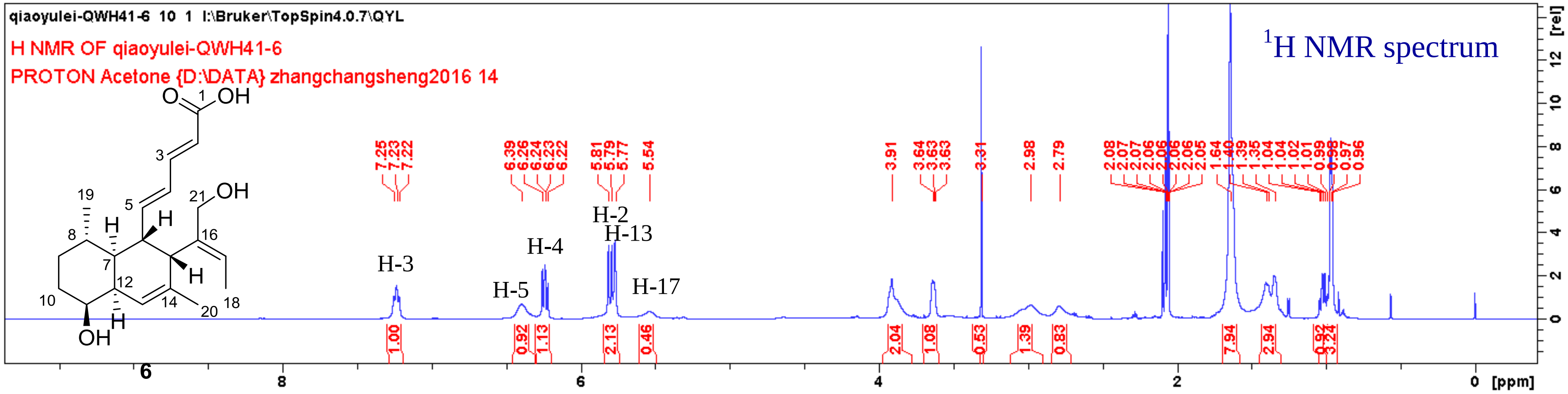

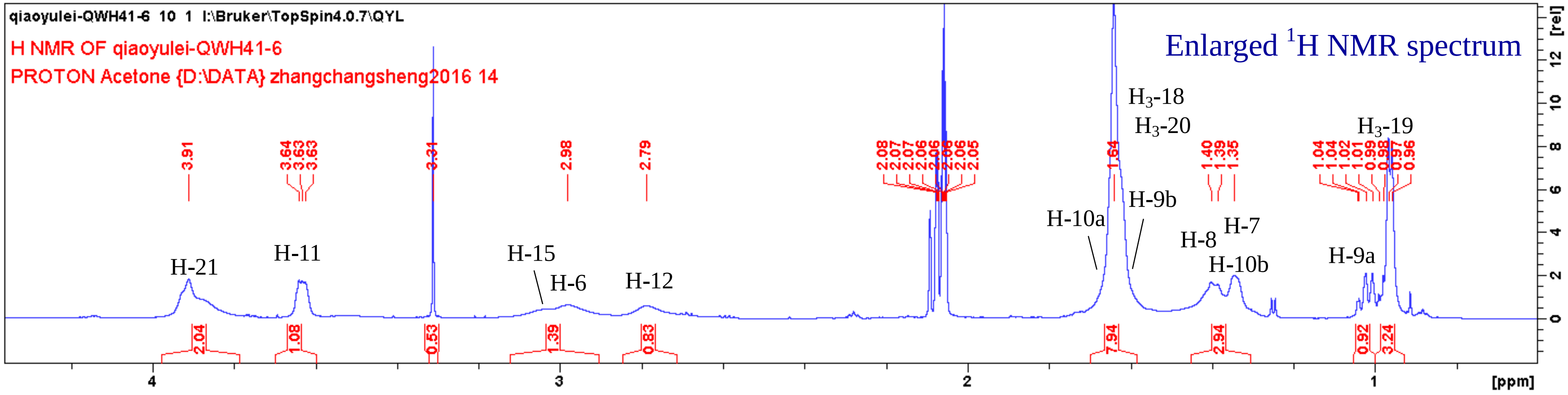

Figure S6. Spectral data for MK8383 D (**6**) (continued).

(E) The ^{13}C NMR and DEPT135 spectra of MK8383 D (**6**) in Acetone-d_6.

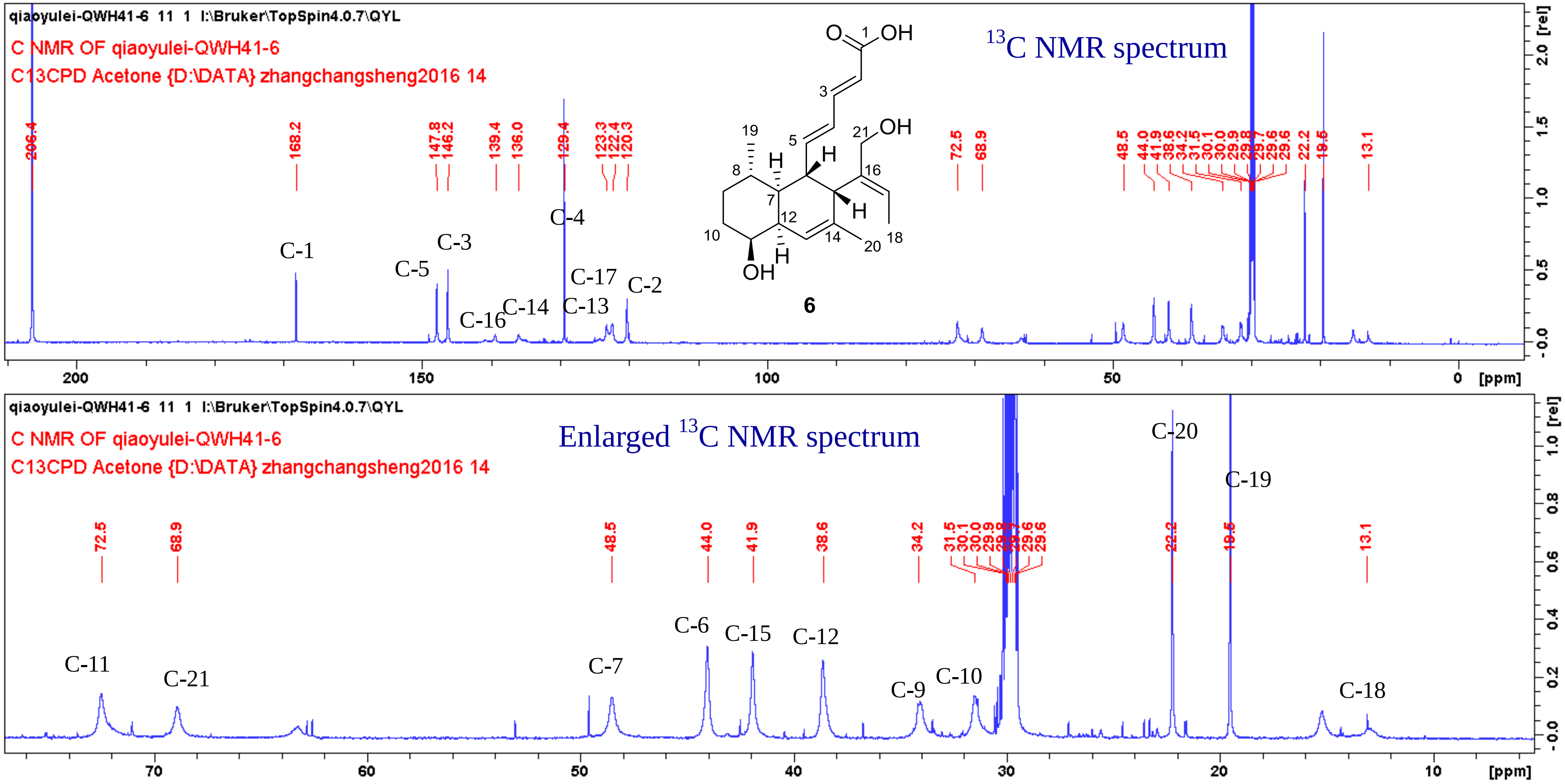

Figure S6. Spectral data for MK8383 D (**6**) (continued).
(F) The HSQC spectrum of MK8383 D (**6**) in Acetone-d_6.

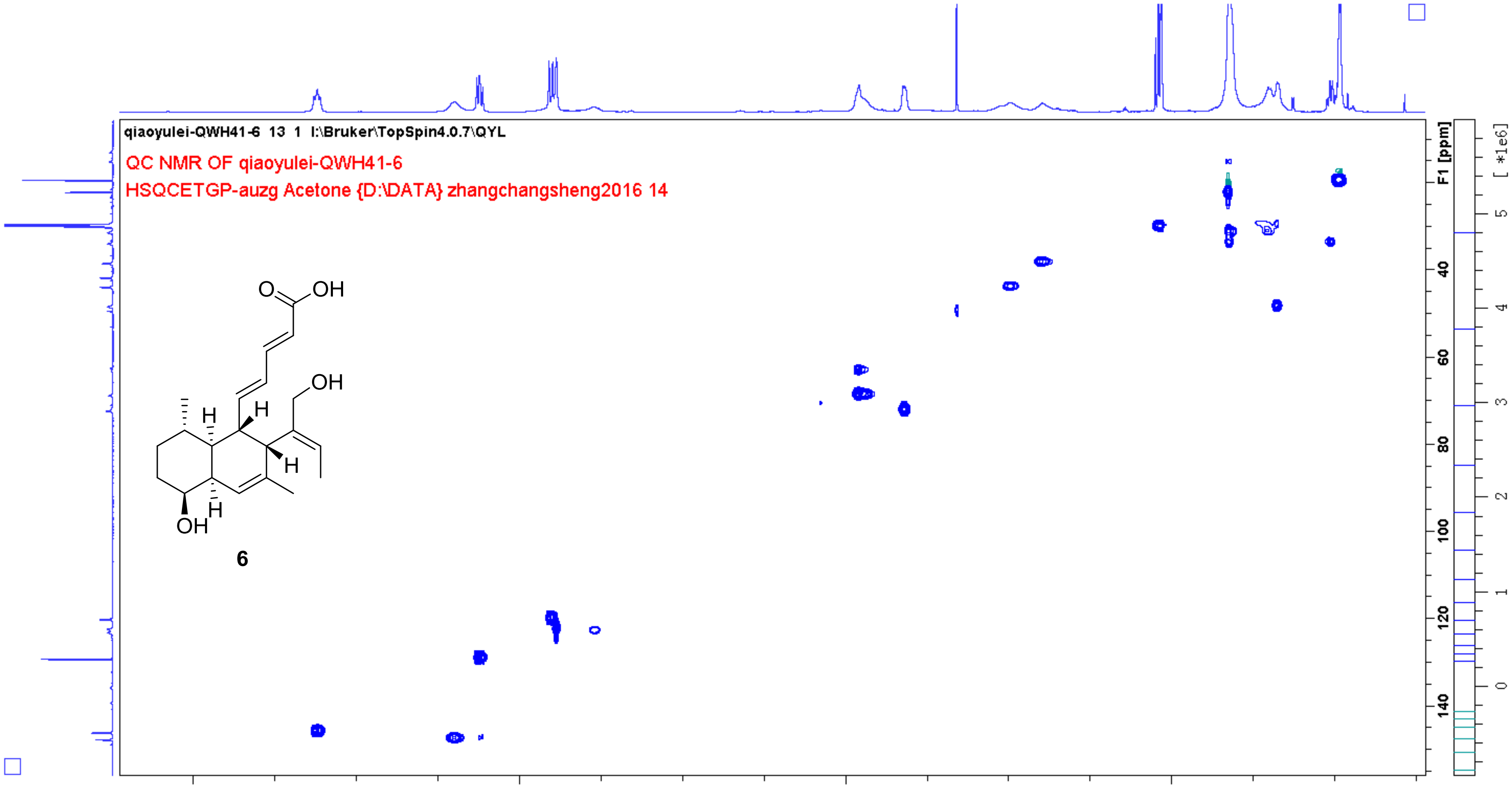

Figure S6. Spectral data for MK8383 D (**6**) (continued).
(G) The ^{1}H-^{1}H COSY spectrum of MK8383 D (**6**) in Acetone-d_6.

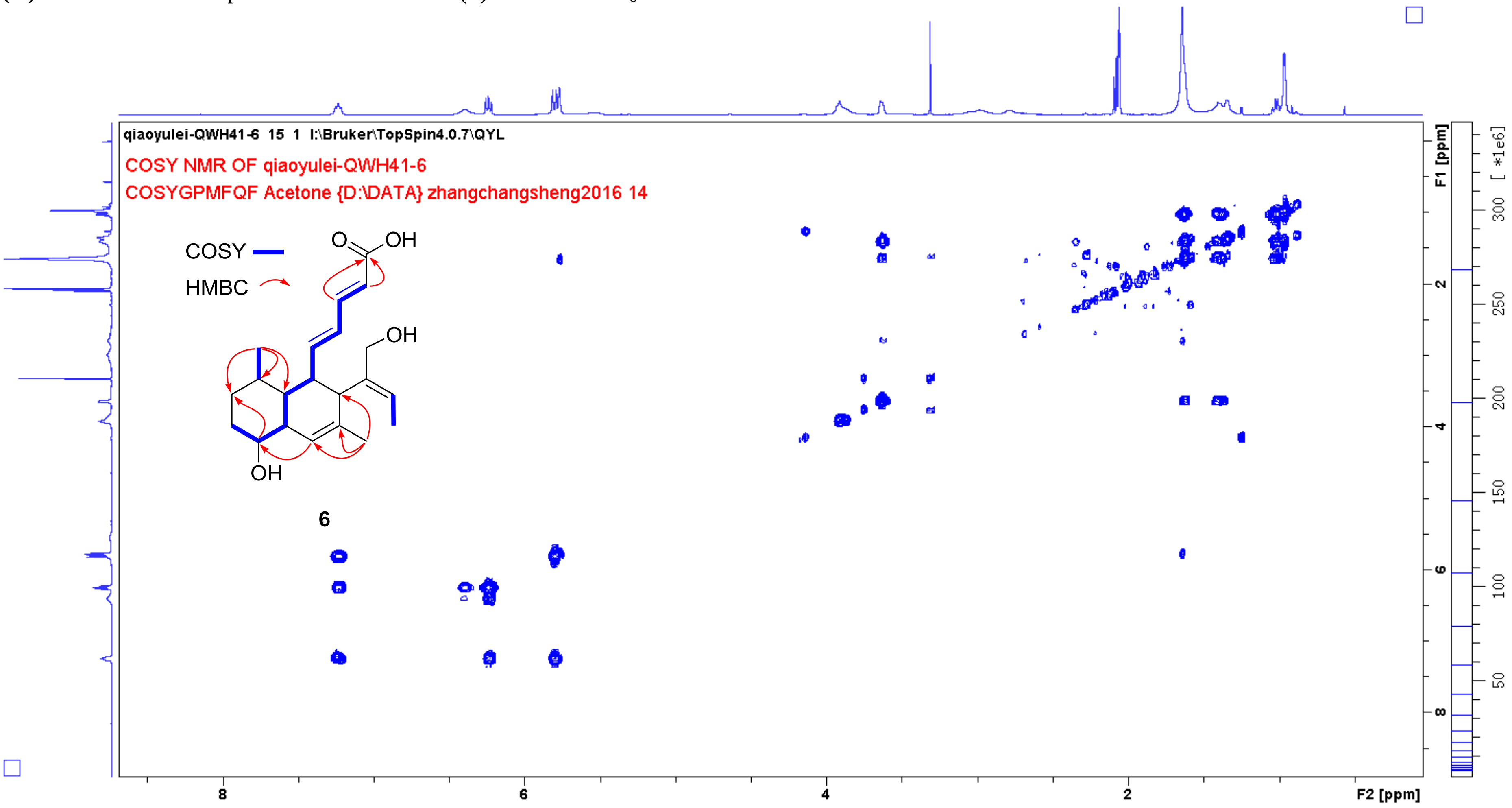

Figure S6. Spectral data for MK8383 D (**6**) (continued).
(H) The HMBC spectrum of MK8383 D (**6**) in Acetone-d_6.

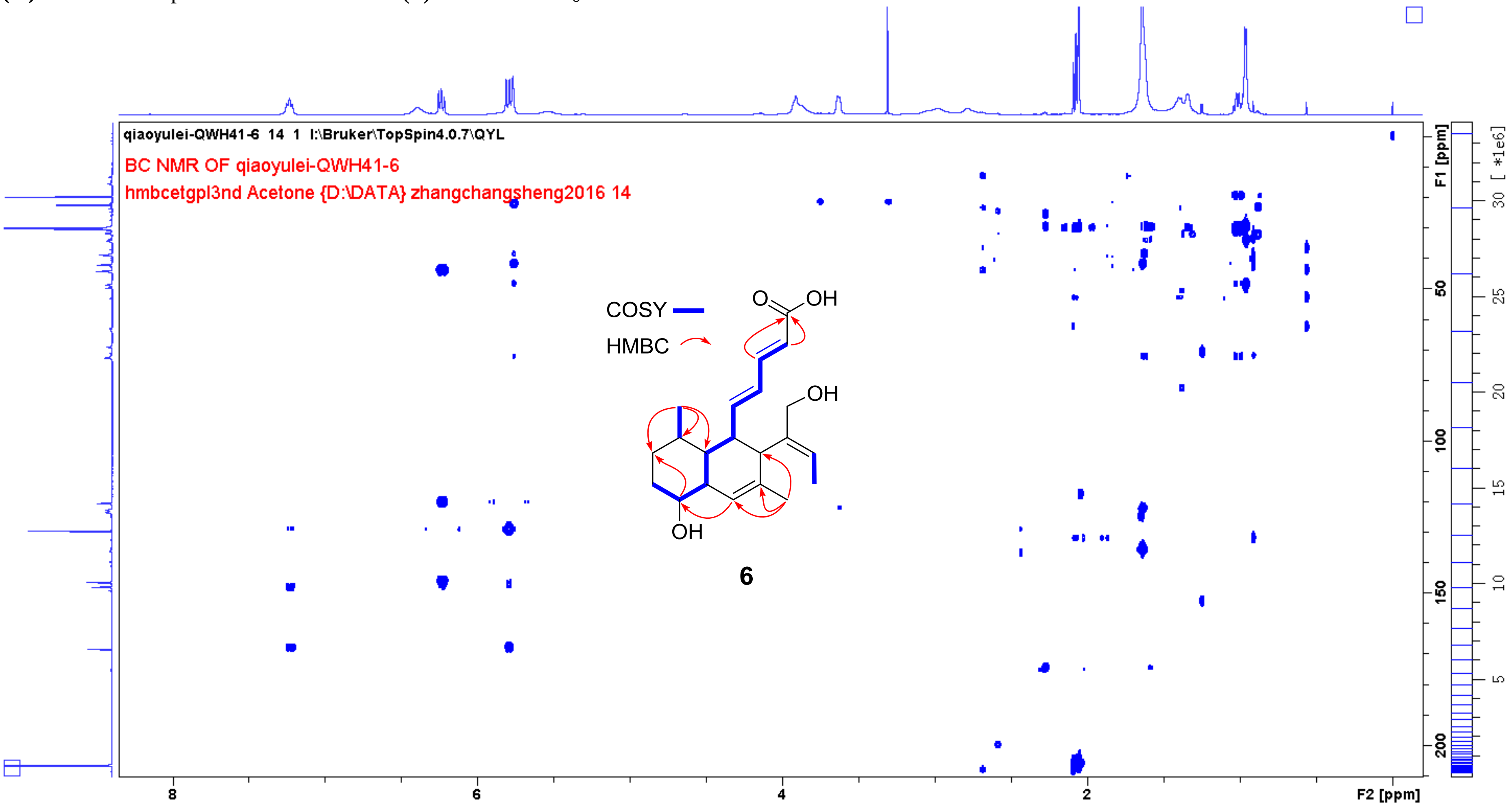

Figure S6. Spectral data for MK8383 D (**6**) (continued).

(I) The NOESY spectrum of MK8383 D (**6**) in Acetone-d_6.

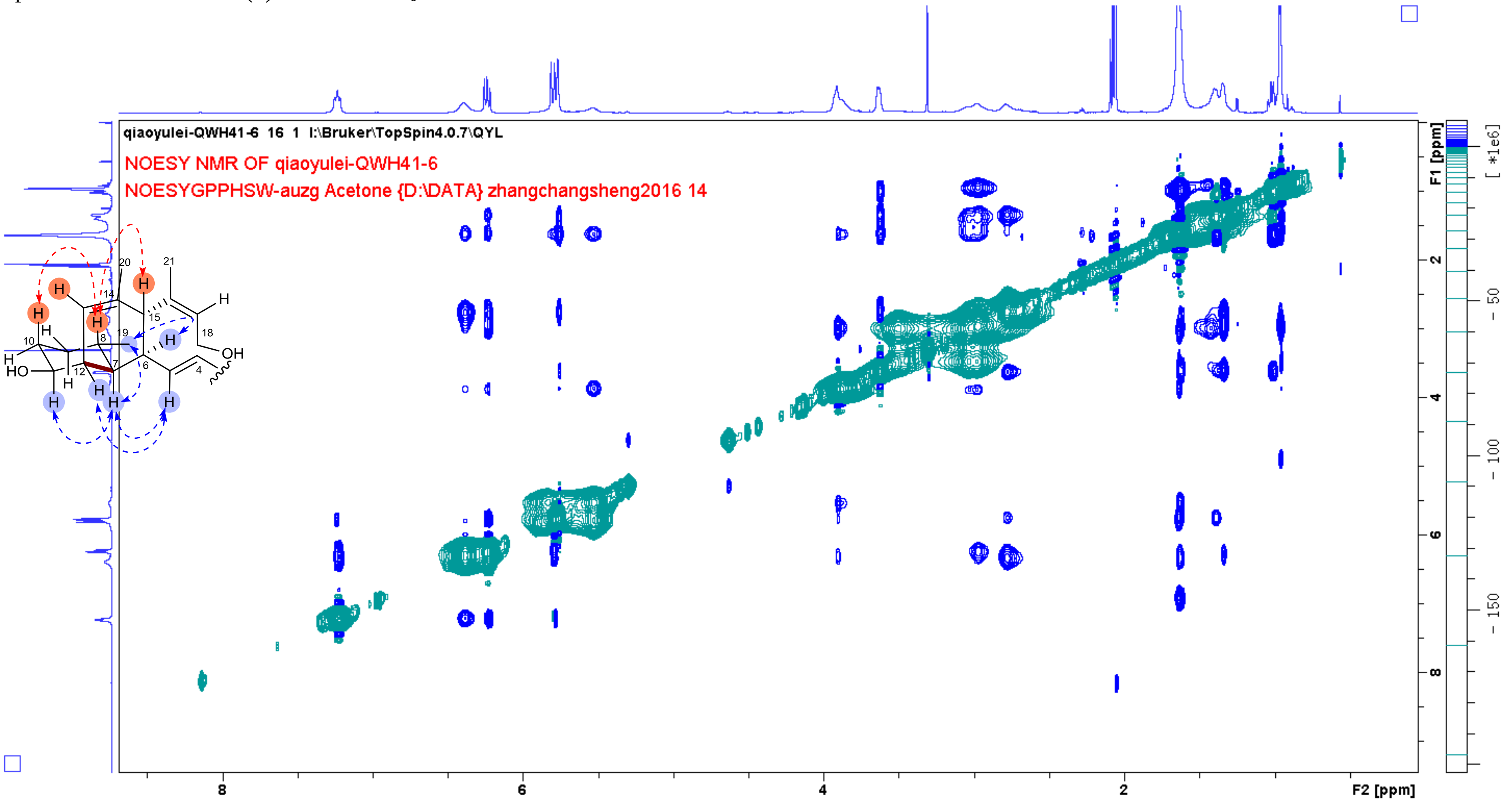

Figure S7. Spectral data for MK8383 E (**7**).

(**A**) HRESIMS

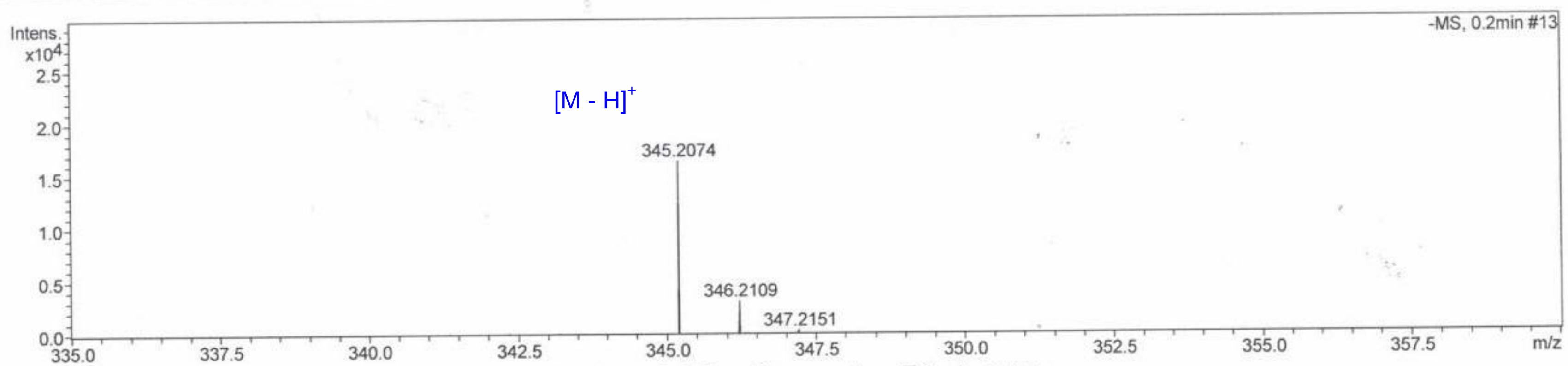

Meas. m/z	#	Ion Formula	Score	m/z	err [ppm]	err [mDa]	mSigma	rdb	e⁻ Conf	N-Rule
345.2074	1	C21H29O4	100.00	345.2071	-0.9	-0.3	23.2	7.5	even	ok
691.4222	1	C42H59O8	100.00	691.4215	-1.0	-0.7	15.9	13.5	even	ok

(**B**) UV

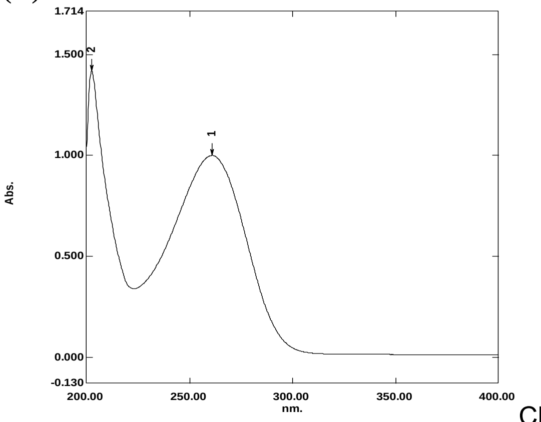

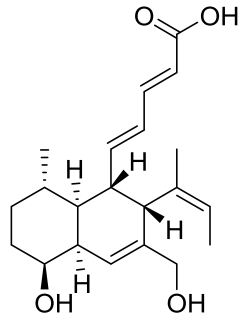

7

Chemical Formula: $C_{21}H_{30}O_4$

Exact Mass: 346.2

(**C**) IR

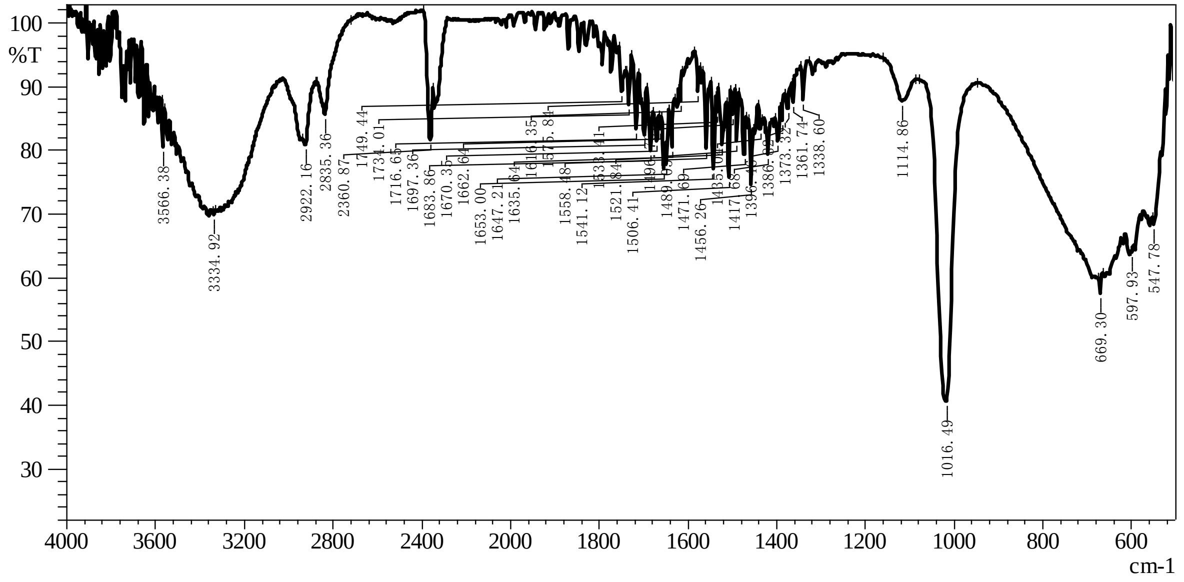

Figure S7. Spectral data for MK8383 E (**7**) (continued).

(D) The ^{1}H and enlarged ^{1}H NMR spectrum of MK8383 E (**7**) in Acetone-d_6.

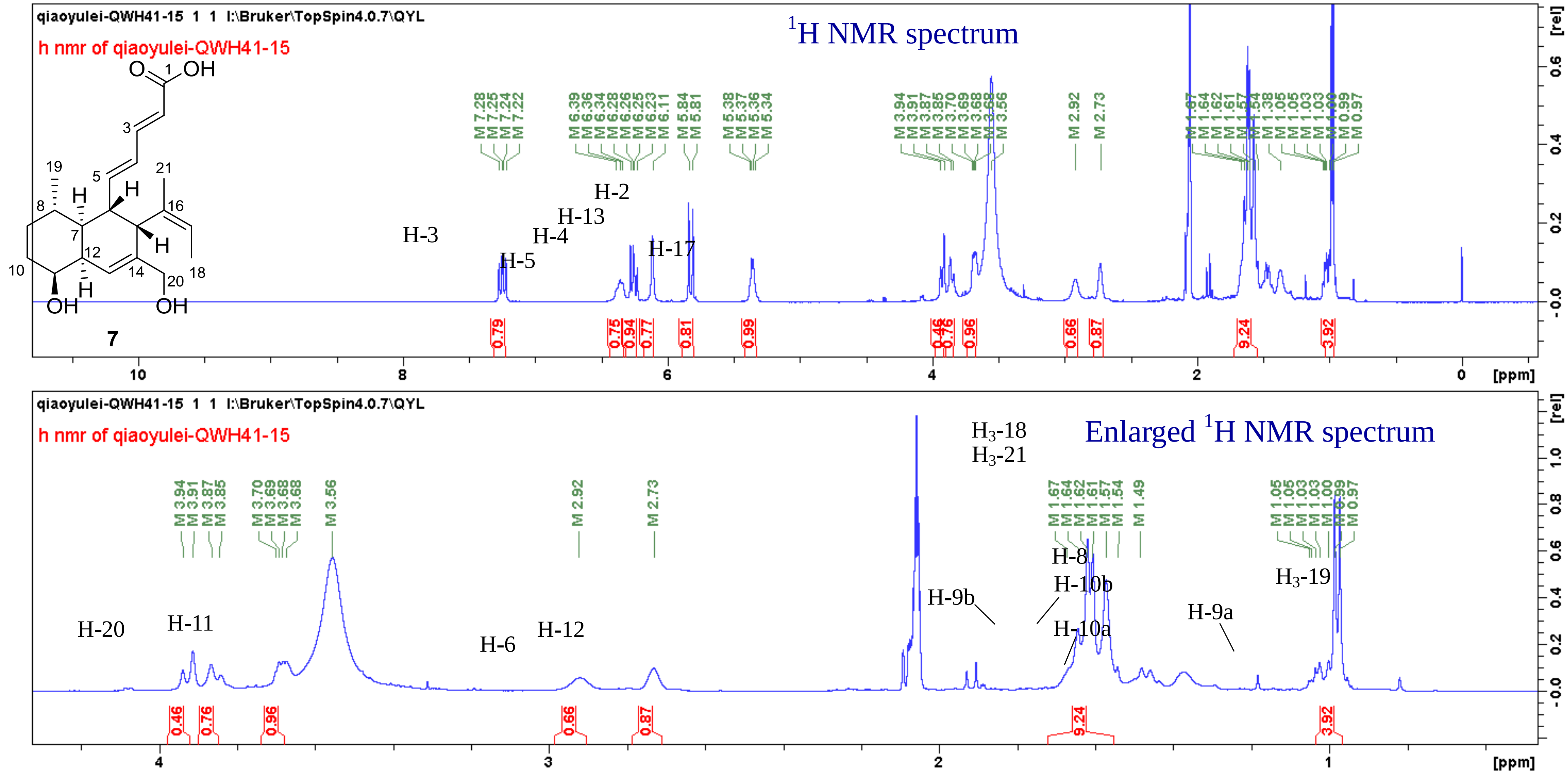

Figure S7. Spectral data for MK8383 E (**7**) (continued).

(E) The ^{13}C and enlarged ^{13}C NMR spectrum of MK8383 E (**7**) in Acetone-d_6.

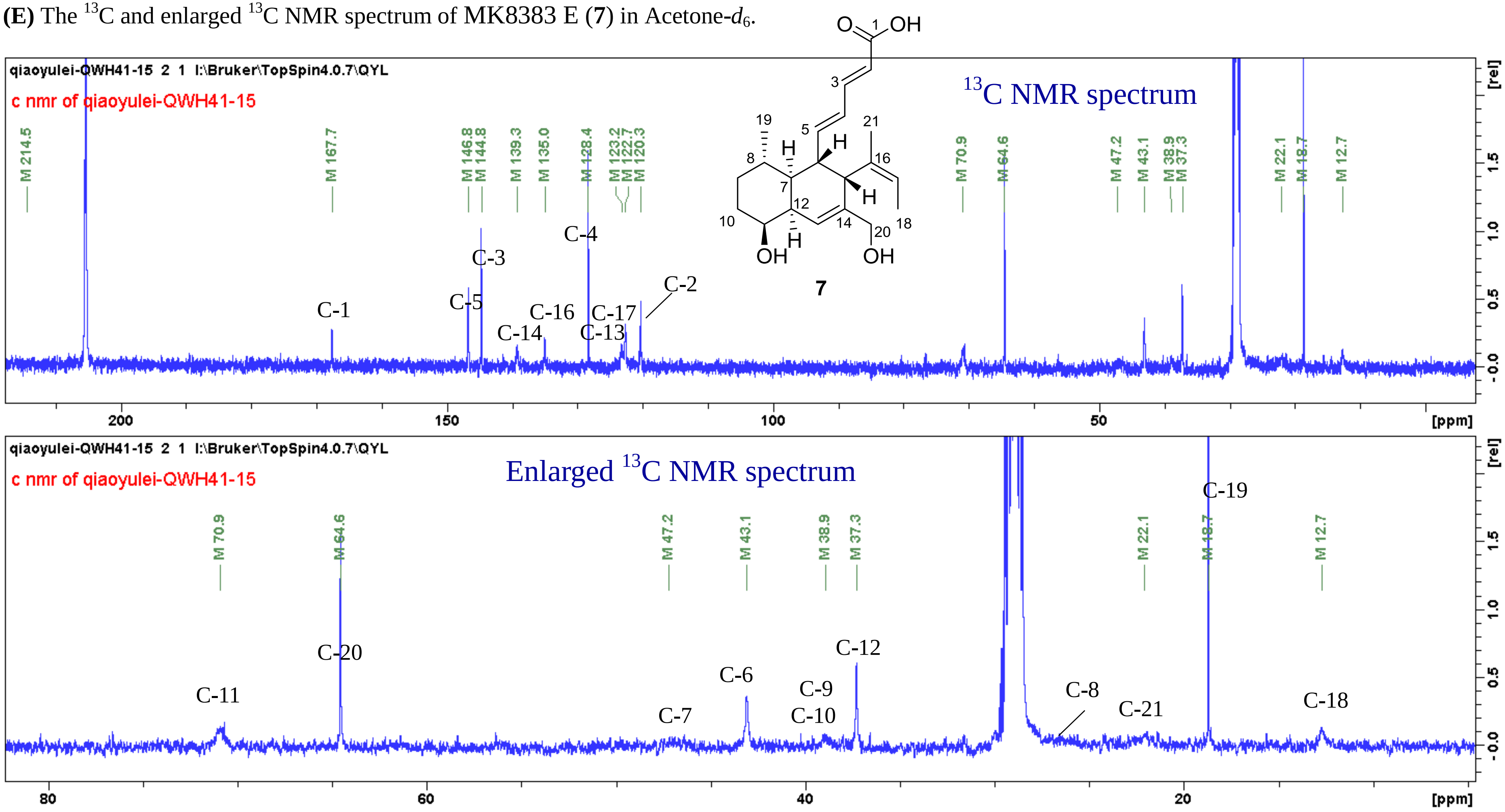

Figure S7. Spectral data for MK8383 E (**7**) (continued).
(F) The HSQC spectrum of MK8383 E (**7**) in Acetone-d_6

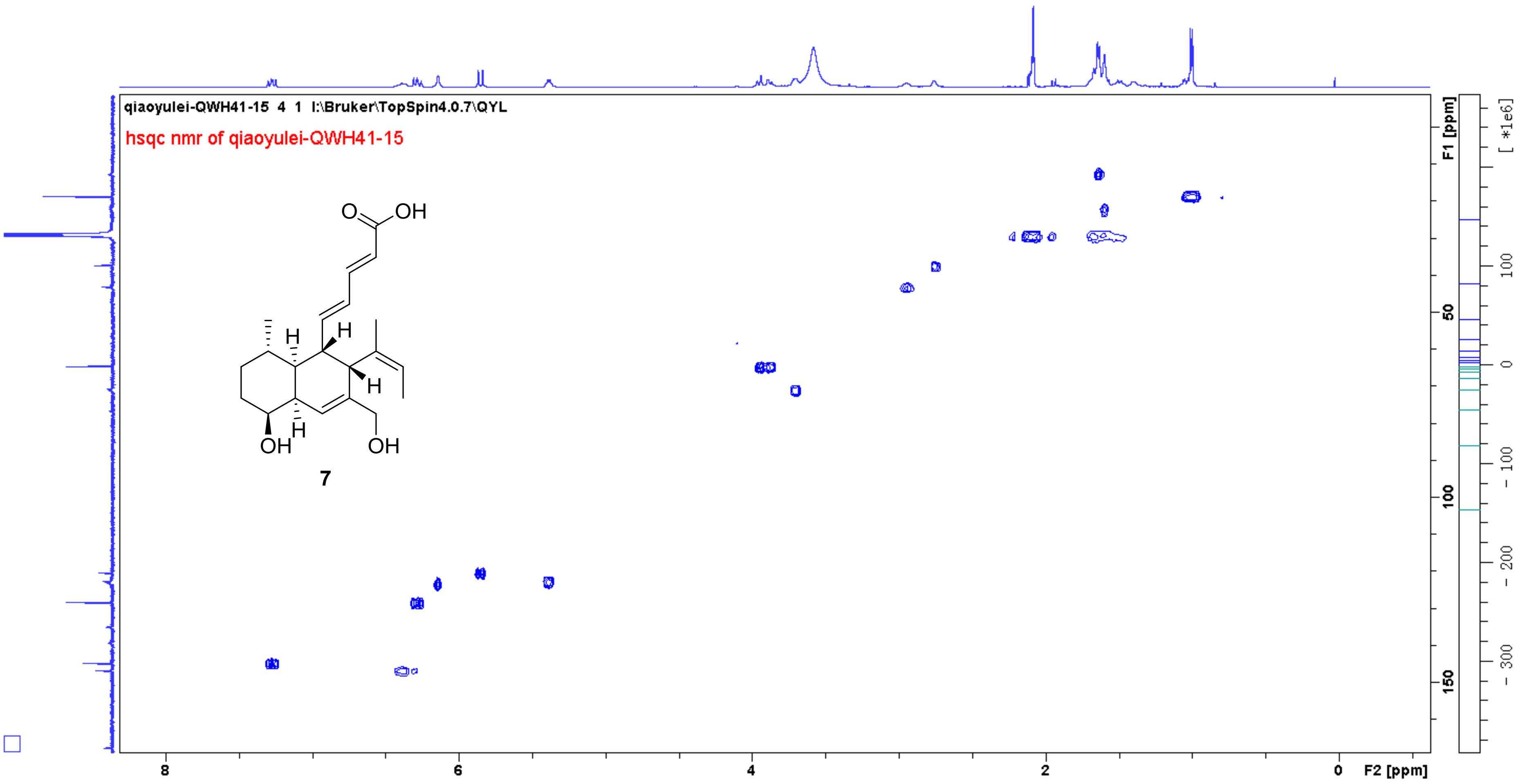

Figure S7. Spectral data for MK8383 E (**7**) (continued).
(G) The ^{1}H-^{1}H COSY spectrum of MK8383 E (**7**) in Acetone-d_6.

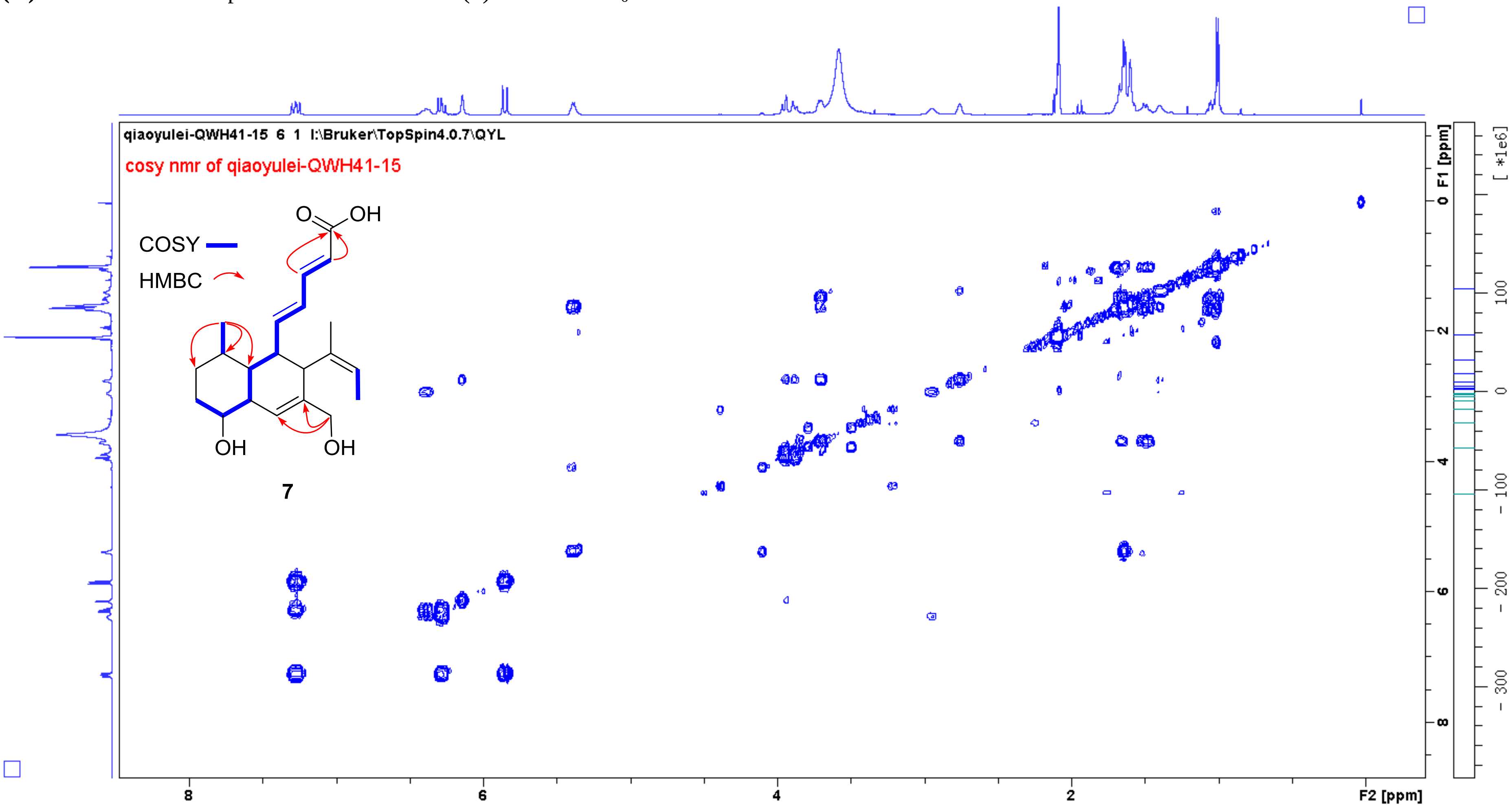

Figure S7. Spectral data for MK8383 E (**7**) (continued).
(H) The HMBC spectrum of MK8383 E (**7**) in Acetone-d_6.

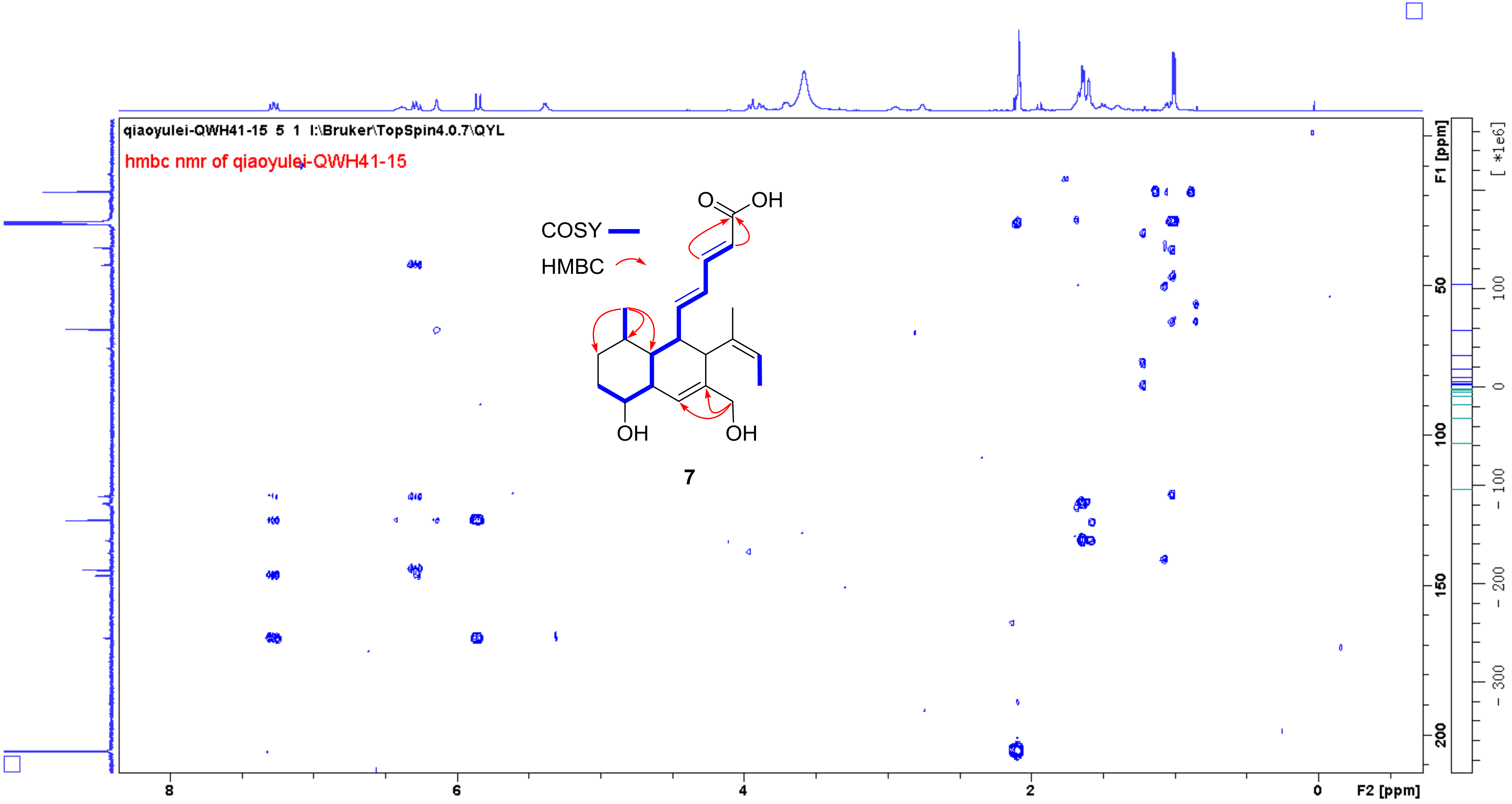

Figure S7. Spectral data for MK8383 E (**7**) (continued).
(I) The NOESY spectrum of MK8383 E (**7**) in Acetone-d_6.

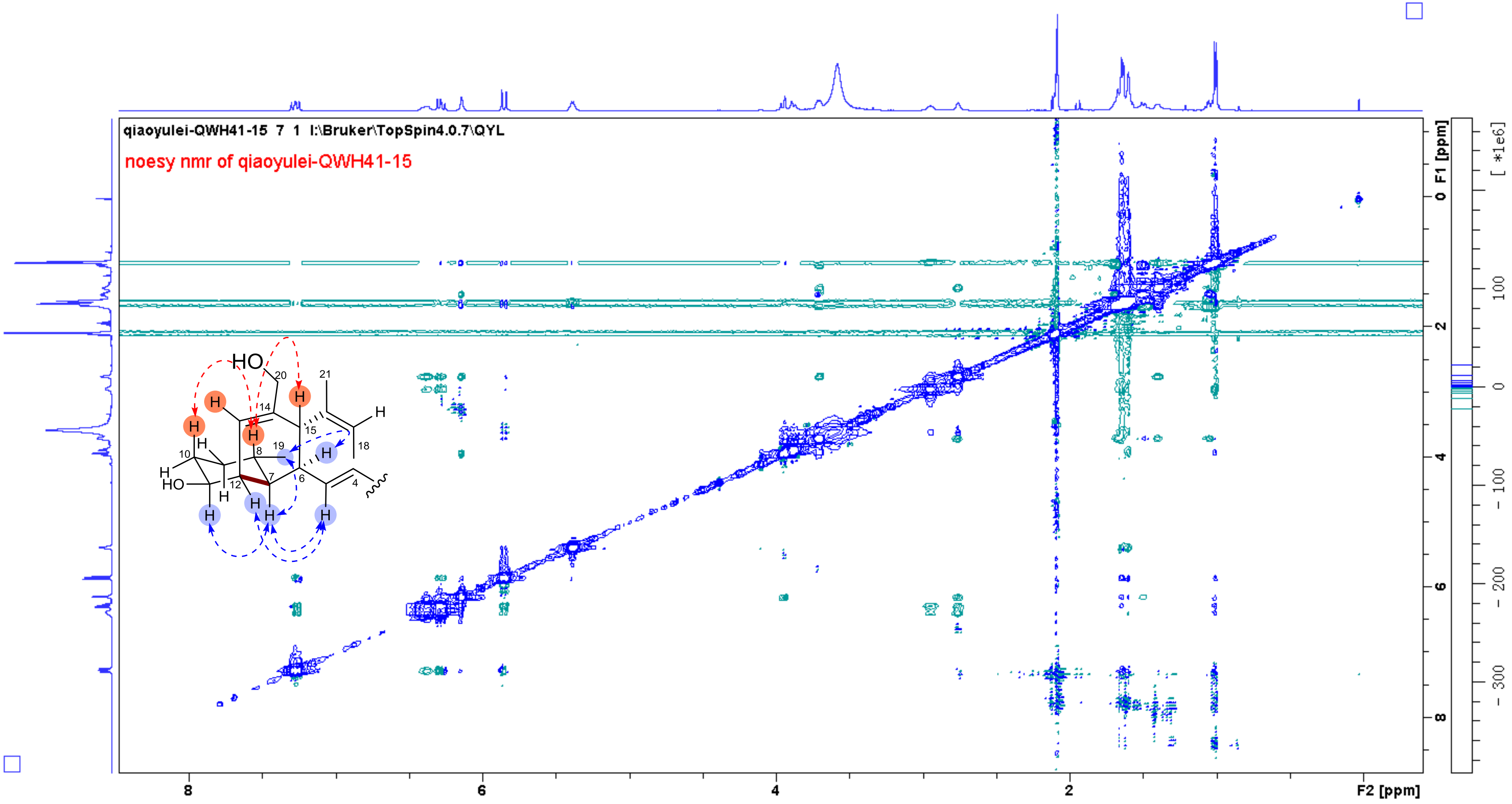

Figure S8. Spectral data for MK8383 F (**8**).

(**A**) HR-ESI-MS

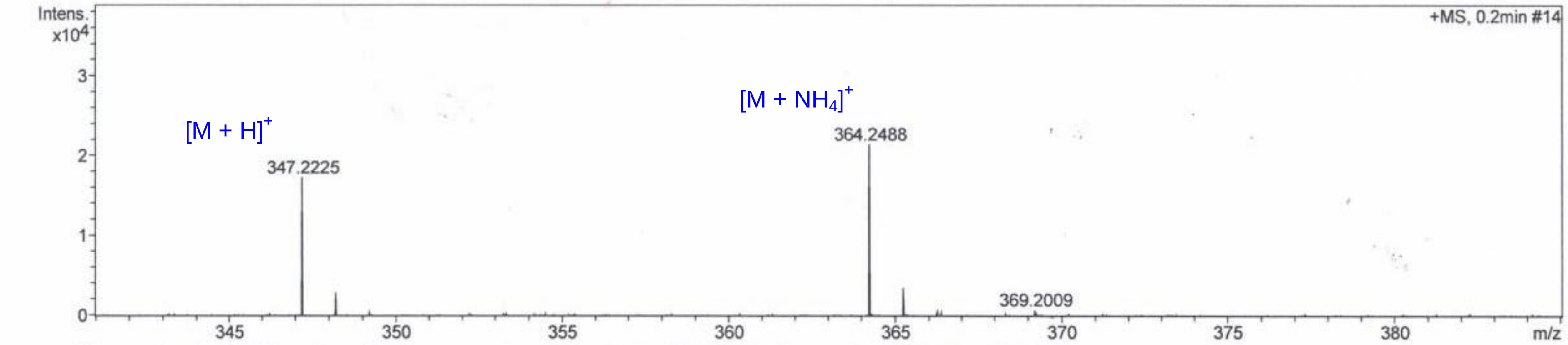

Meas. m/z	#	Ion Formula	Score	m/z	err [ppm]	err [mDa]	mSigma	rdb	e¯ Conf	N-Rule
347.2225	1	C21H31O4	100.00	347.2217	-2.3	-0.8	32.7	6.5	even	ok
364.2488	1	C21H34NO4	100.00	364.2482	-1.6	-0.6	37.4	5.5	even	ok

(**B**) UV

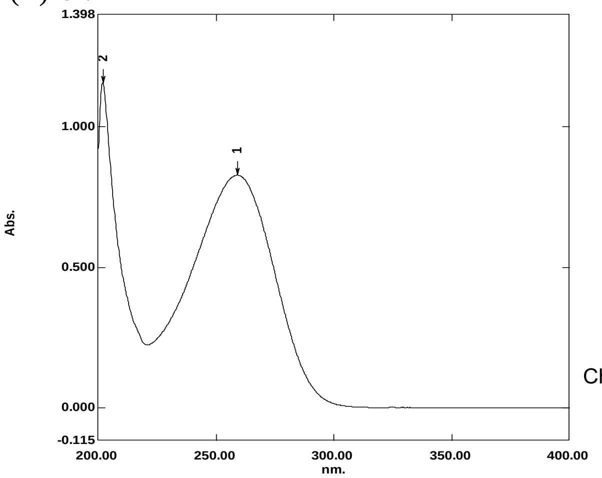

(

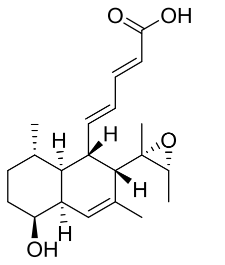

8

Chemical Formula: $C_{21}H_{30}O_4$

Exact Mass: 346.2

C) IR

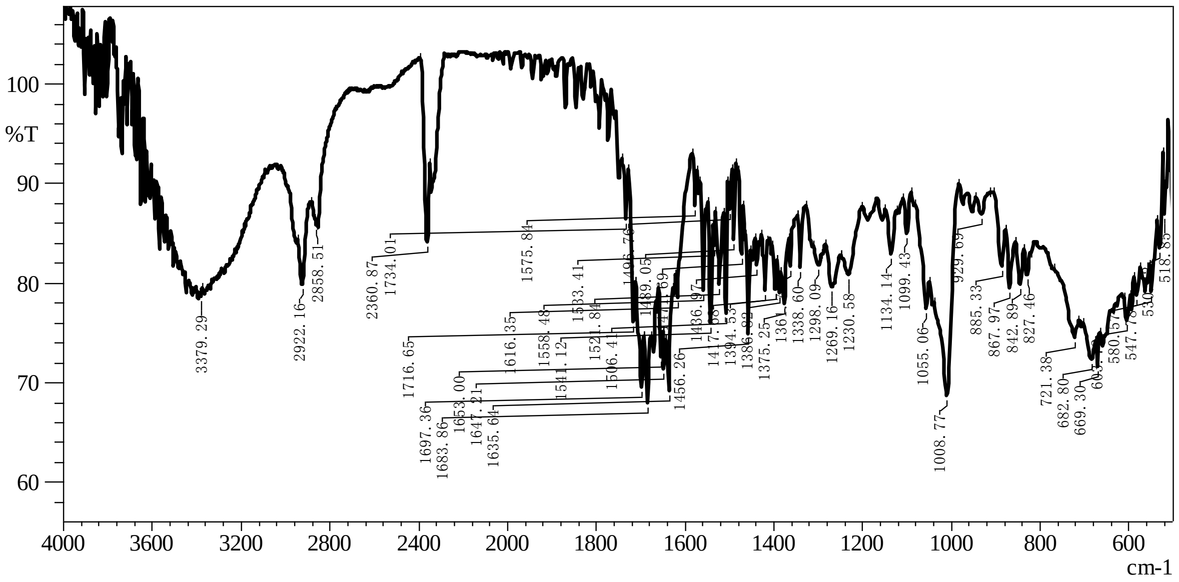

Figure S8. Spectral data for MK8383 F (**8**) (continued).
(D) The ^{1}H and enlarged ^{1}H NMR spectrum of MK8383 F (**8**) in Methanol-d_4.

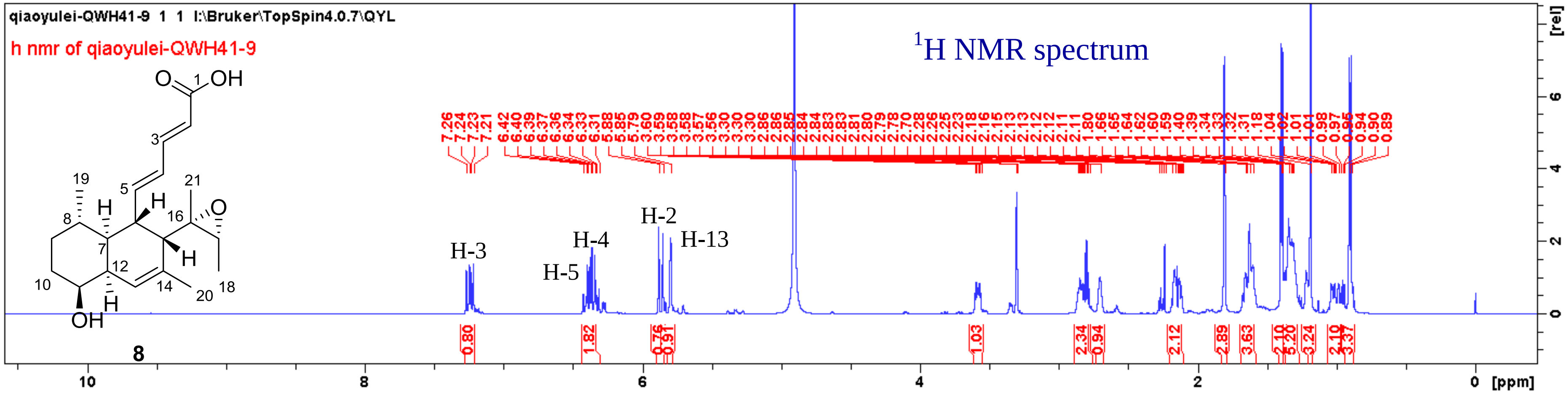

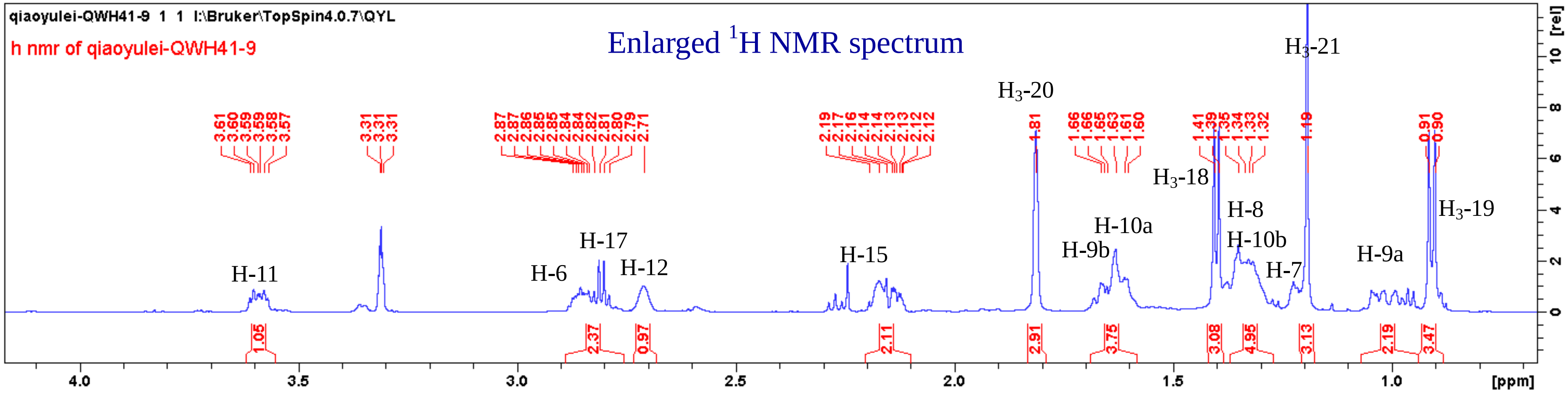

Figure S8. Spectral data for MK8383 F (**8**) (continued).
(E) The ^{13}C and enlarged ^{13}C NMR spectra of MK8383 F (**8**) in Methanol-d_4.

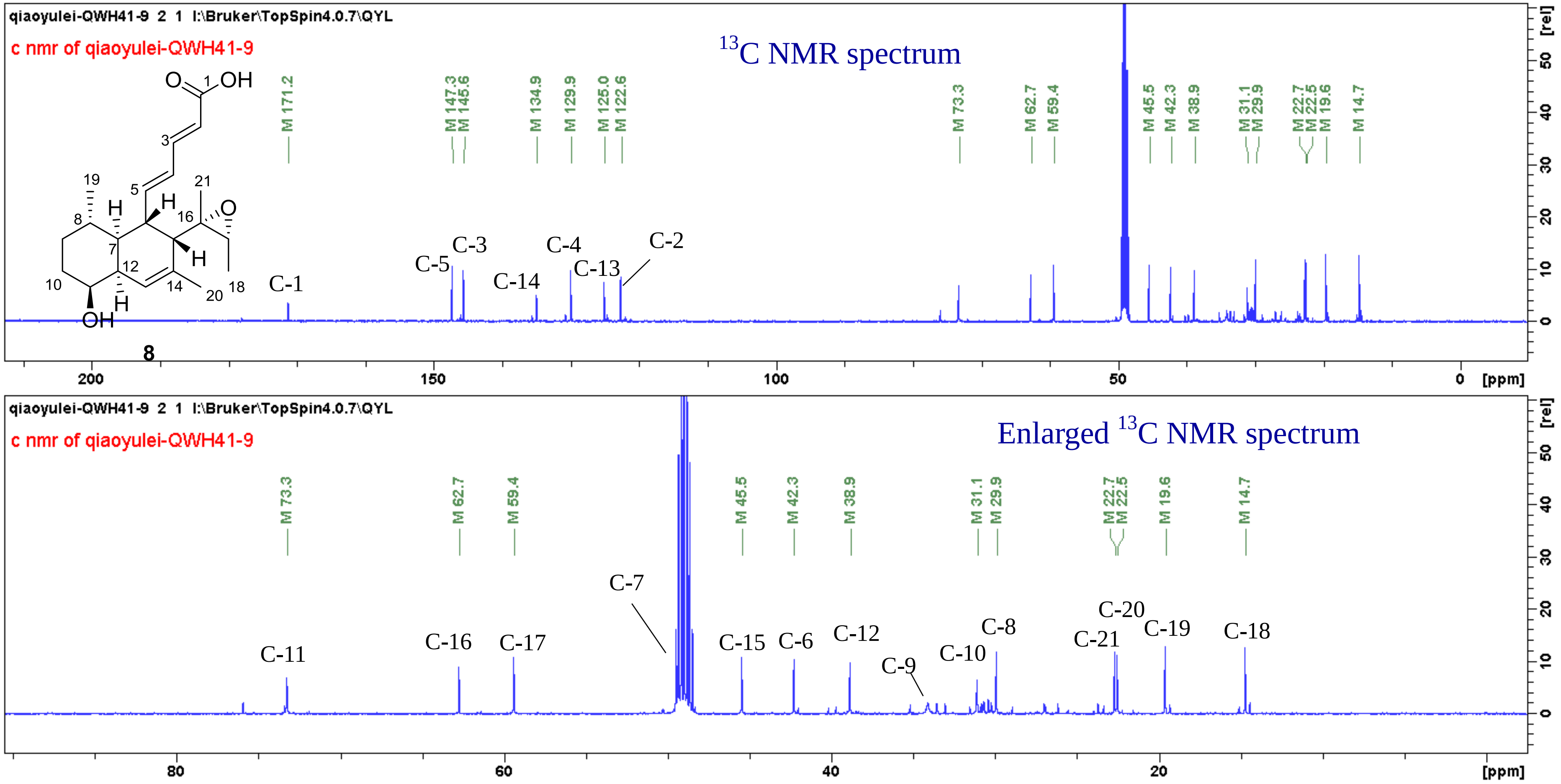

Figure S8. Spectral data for MK8383 F (**8**) (continued).
(F) The HSQC spectrum of MK8383 F (**8**) in Methanol-d_4.

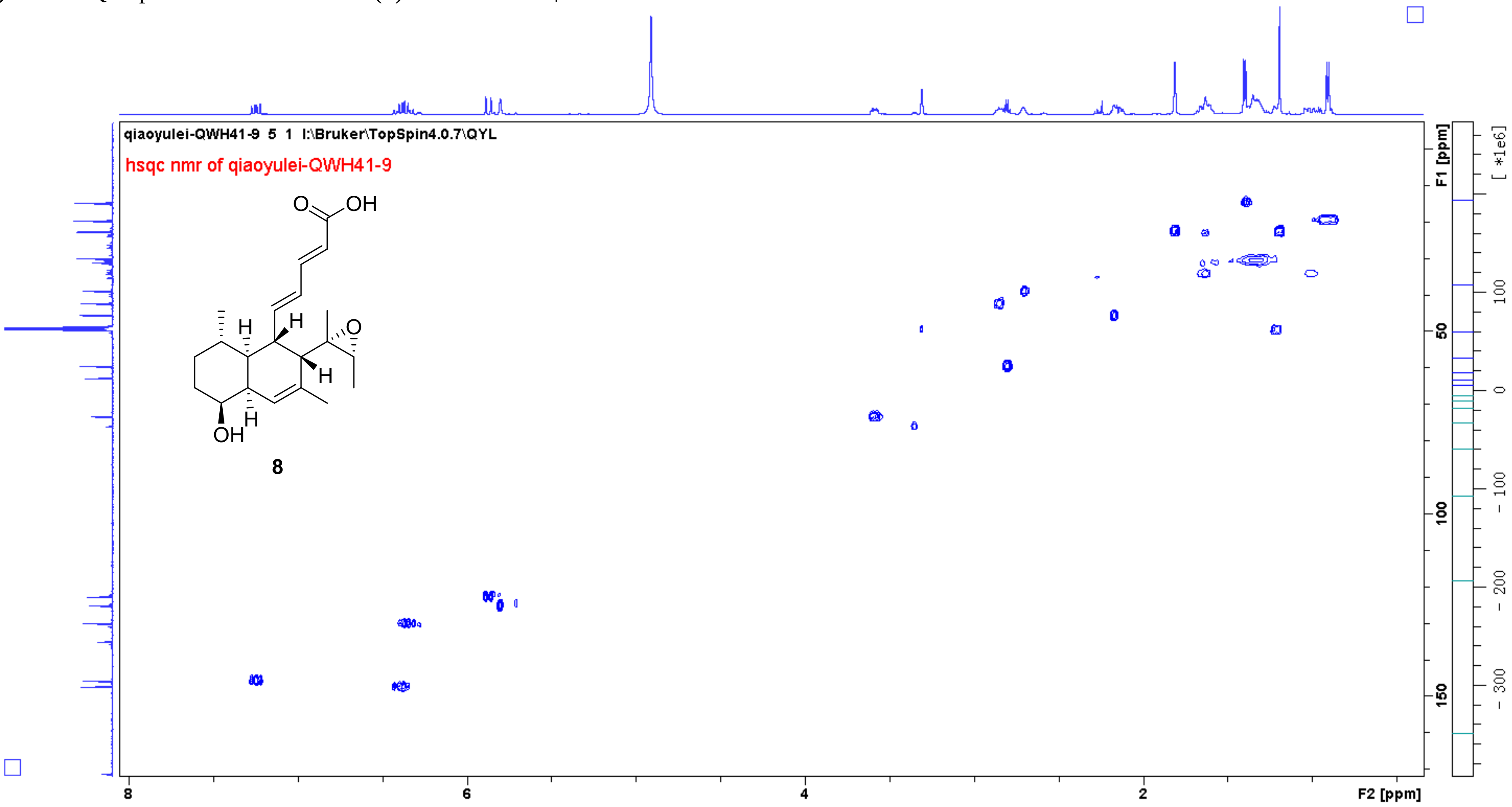

Figure S8. Spectral data for MK8383 F (**8**) (continued).
(G) The ^1H-^1H COSY spectrum of MK8383 F (**8**) in Methanol-d_4.

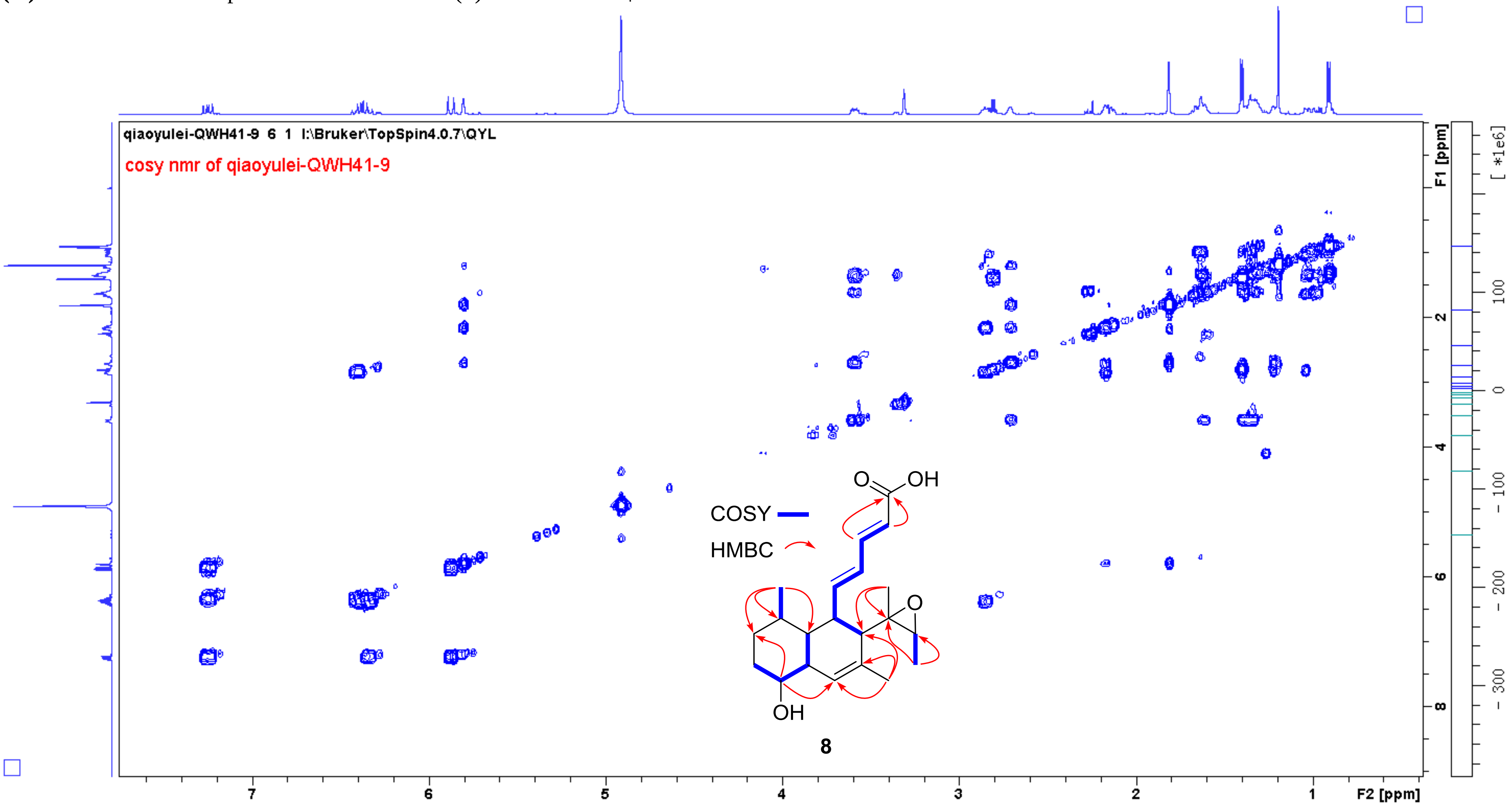

Figure S8. Spectral data for MK8383 F (**8**) (continued).
(H) The HMBC spectrum of MK8383 F (**8**) in Methanol-d_4.

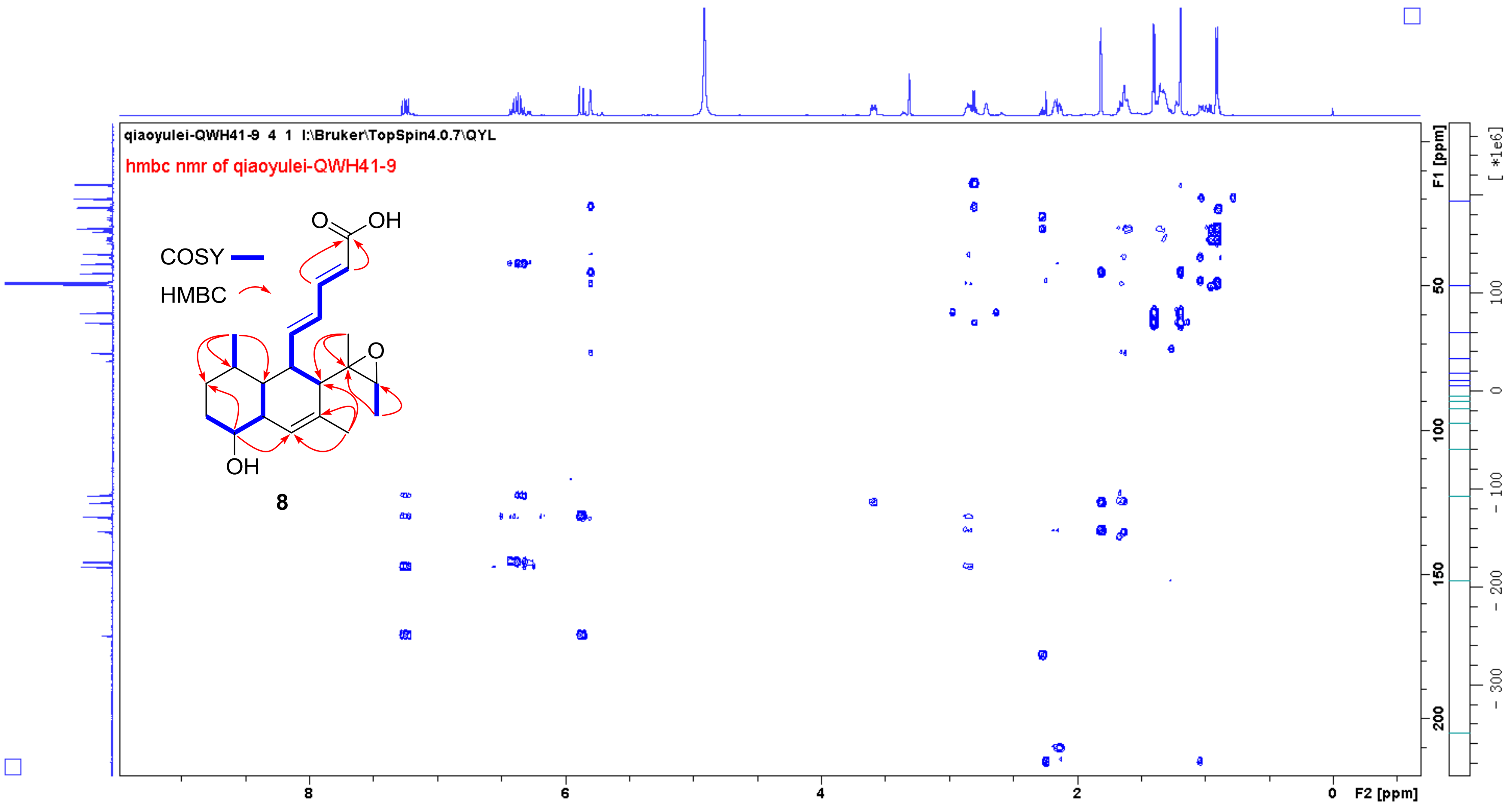

Figure S8. Spectral data for MK8383 F (**8**) (continued).
(I) The NOESY spectrum of MK8383 F (**8**) in Methanol-d_4.

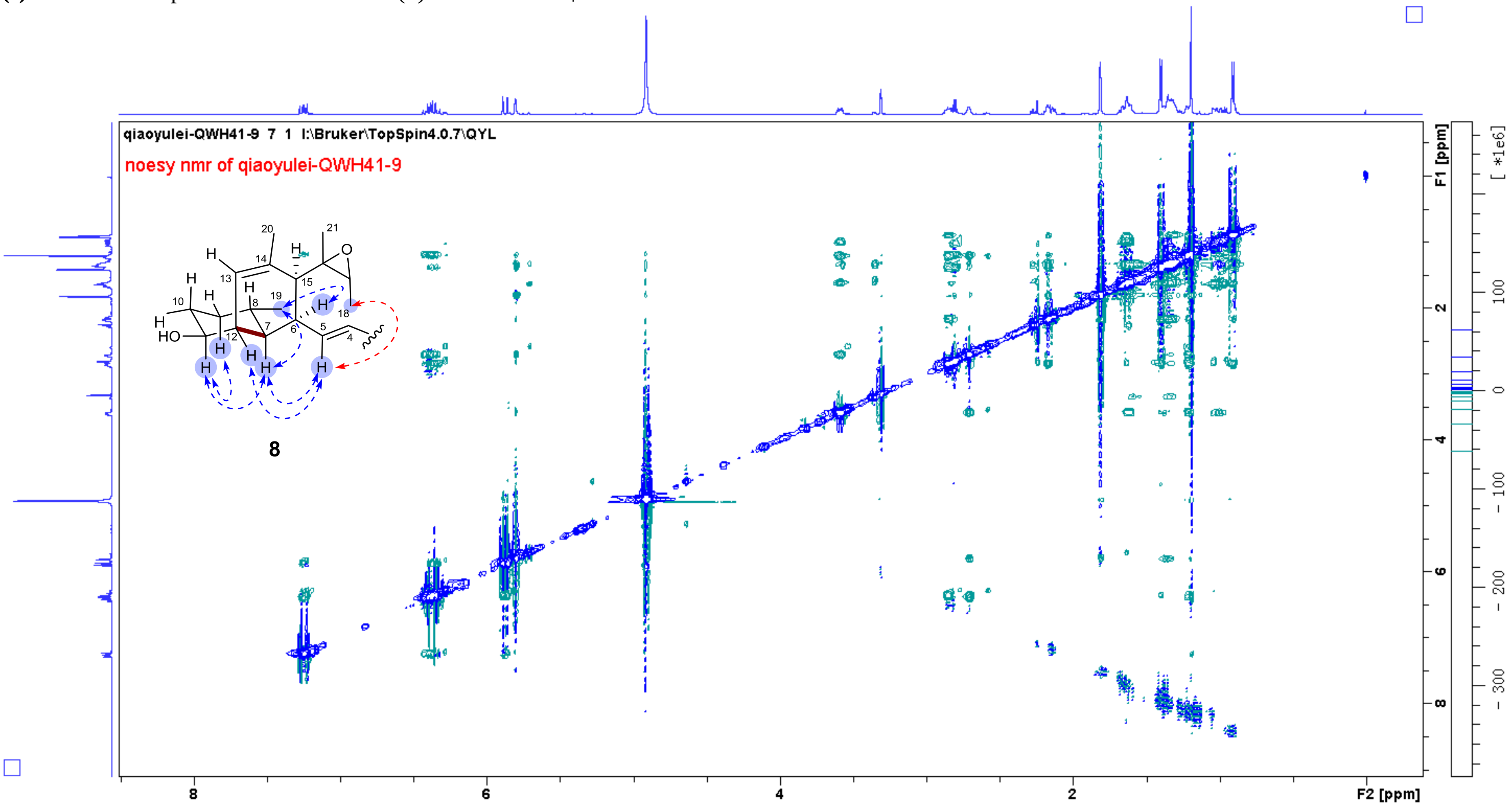

Figure S9. Spectral data for MK8383 G (**9**)

(**A**) HRESIMS

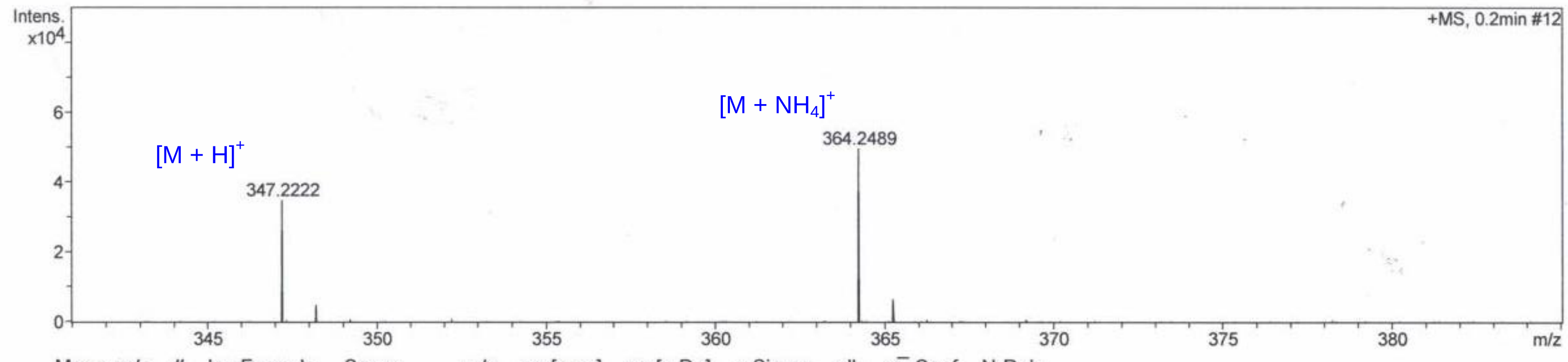

Meas. m/z	#	Ion Formula	Score	m/z	err [ppm]	err [mDa]	mSigma	rdb	e⁻ Conf	N-Rule
347.2222	1	C21H31O4	100.00	347.2217	-1.6	-0.5	45.2	6.5	even	ok
364.2489	1	C21H34NO4	100.00	364.2482	1.8	0.7	53.6	5.5	even	ok

(**B**) UV

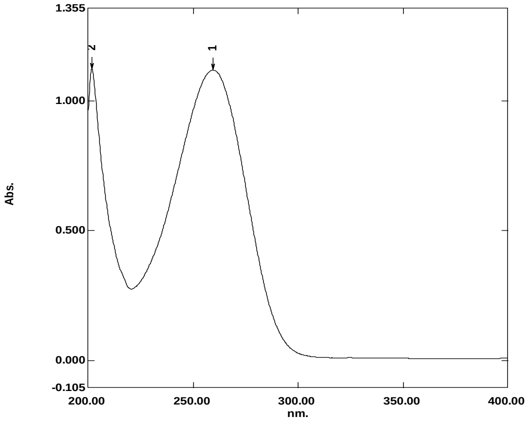

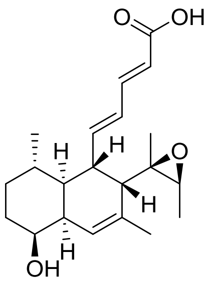

9

Chemical Formula: $C_{21}H_{30}O_4$
Exact Mass: 346.2

(**C**) IR

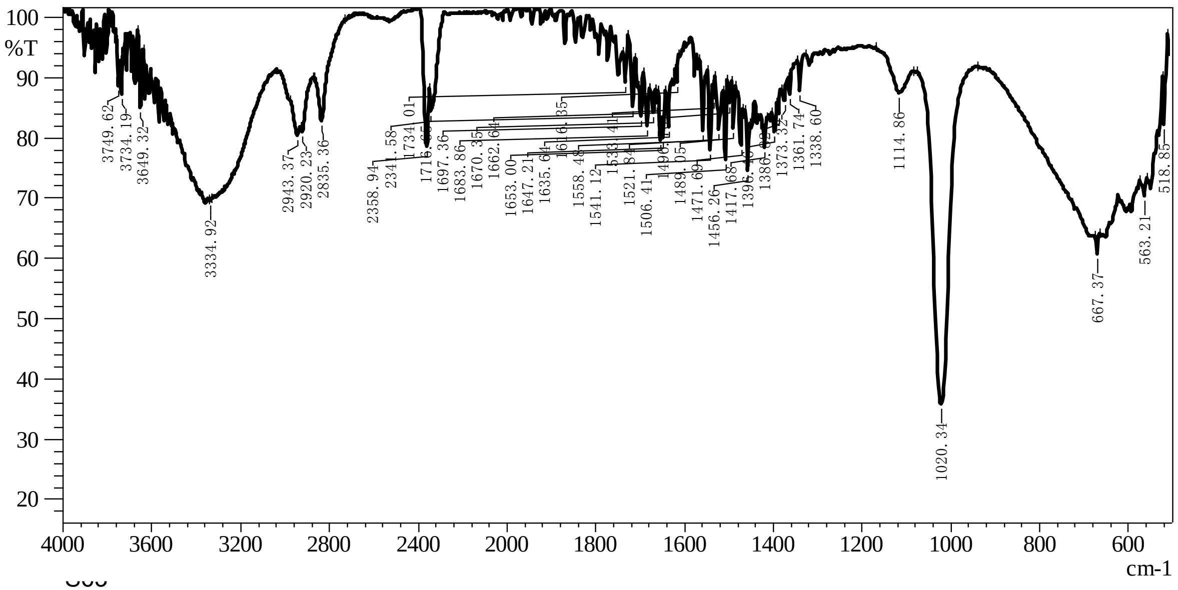

Figure S9. Spectral data for MK8383 G (**9**) (continued).

(D) The ^{1}H and enlarged ^{1}H NMR spectrum of MK8383 G (**9**) in DMSO-d_6.

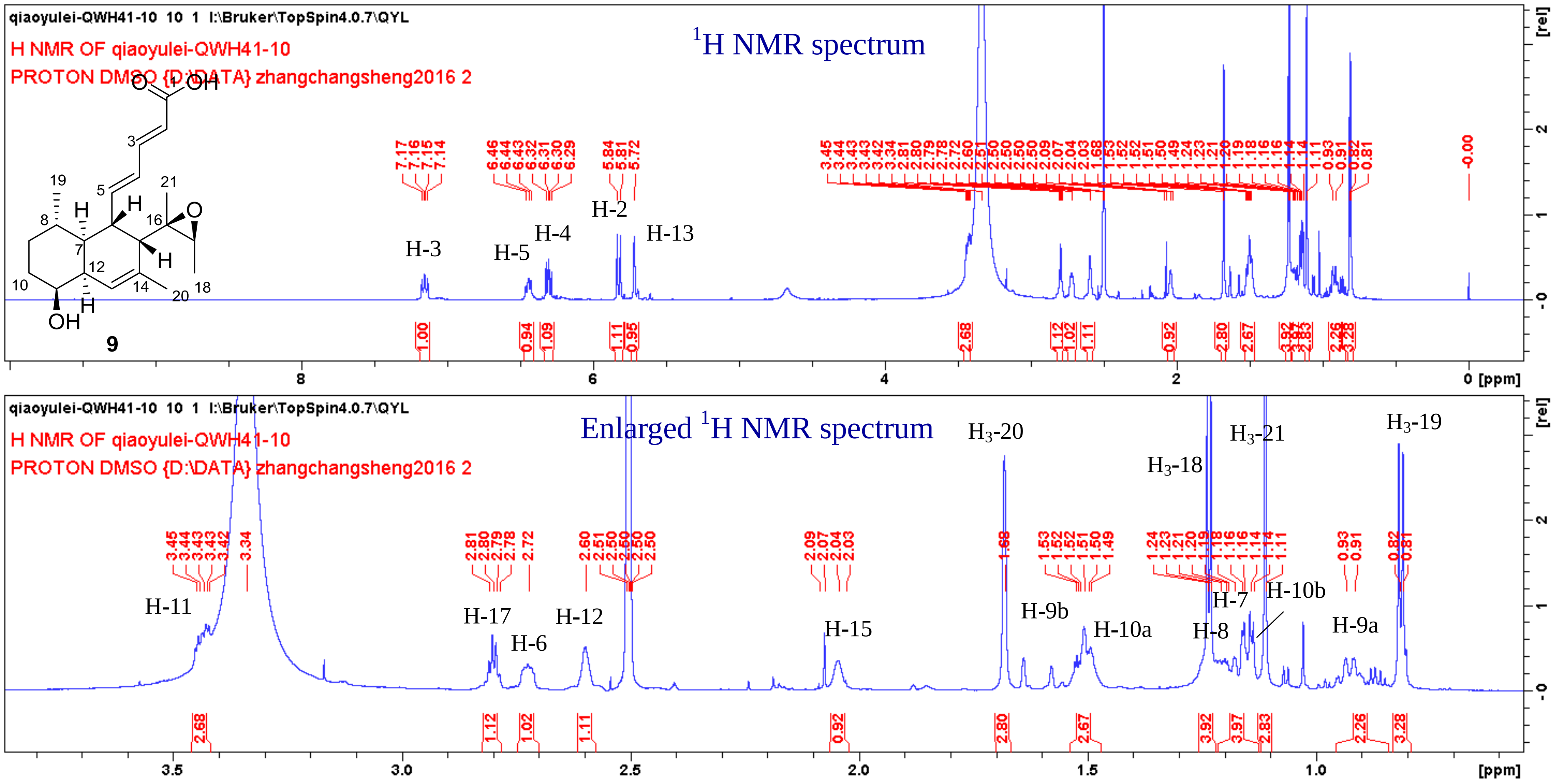

Figure S9. Spectral data for MK8383 G (**9**) (continued).
(E) The ^{13}C and enlarged ^{13}C NMR spectra of MK8383 G (**9**) in DMSO-d_6.

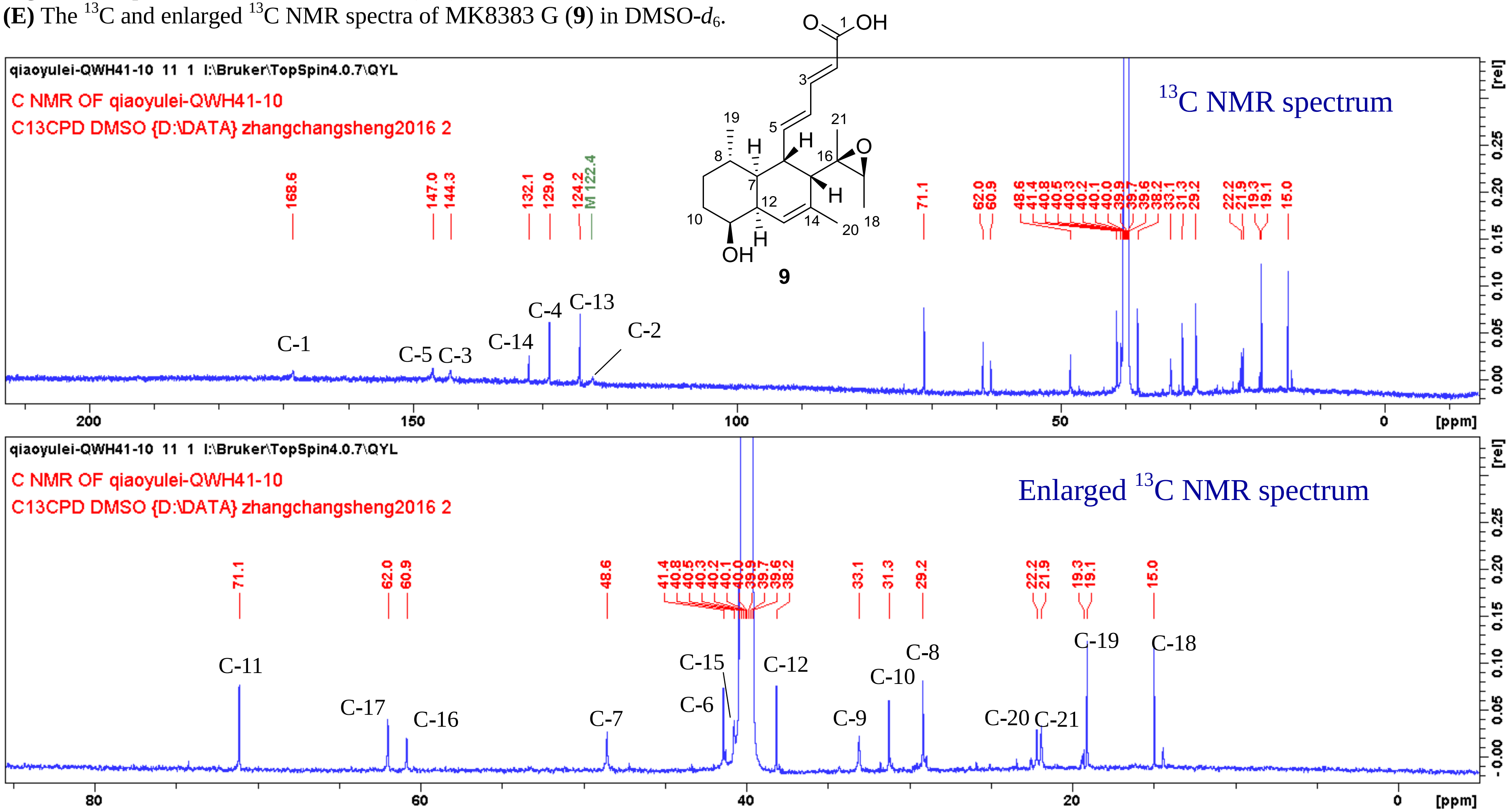

Figure S9. Spectral data for MK8383 G (**9**) (continued).
(F) The HSQC spectrum of MK8383 G (**9**) in DMSO-d_6.

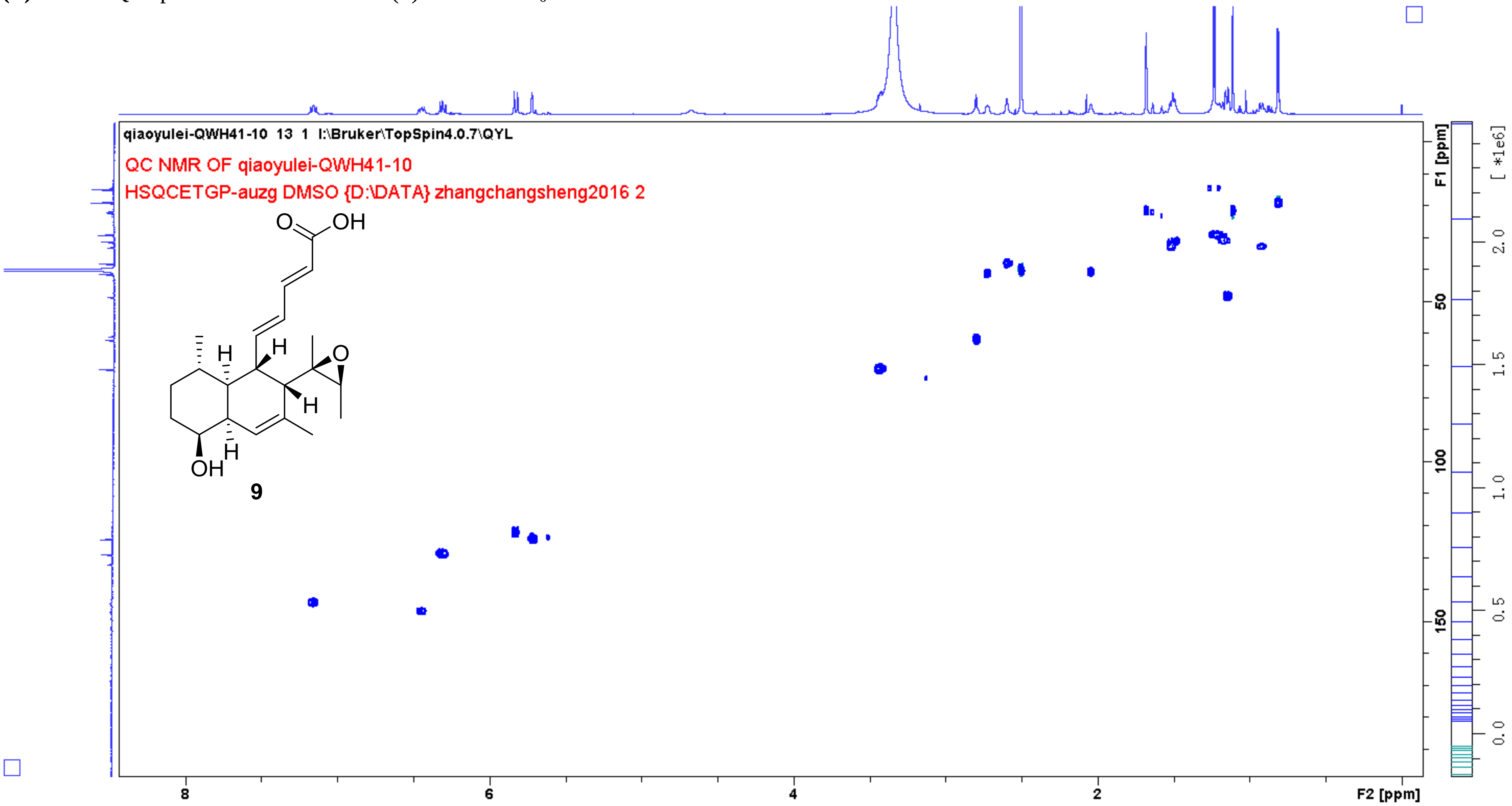

Figure S9. Spectral data for MK8383 G (**9**) (continued).
(G) The ^{1}H-^{1}H COSY spectrum of MK8383 G (**9**) in DMSO-d_6.

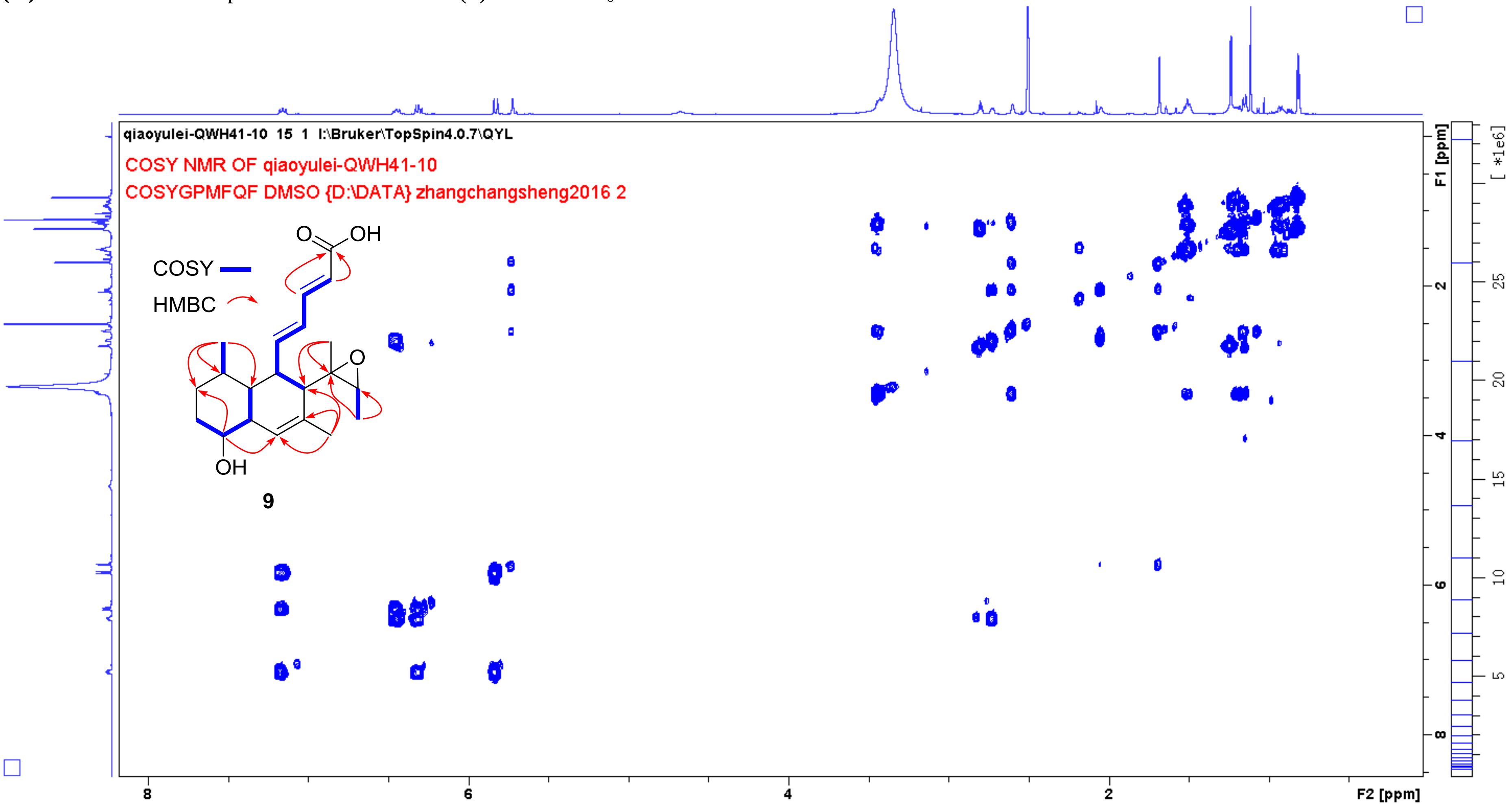

Figure S9. Spectral data for MK8383 G (**9**) (continued).
(H) The HMBC spectrum of MK8383 G (**9**) in DMSO-d_6.

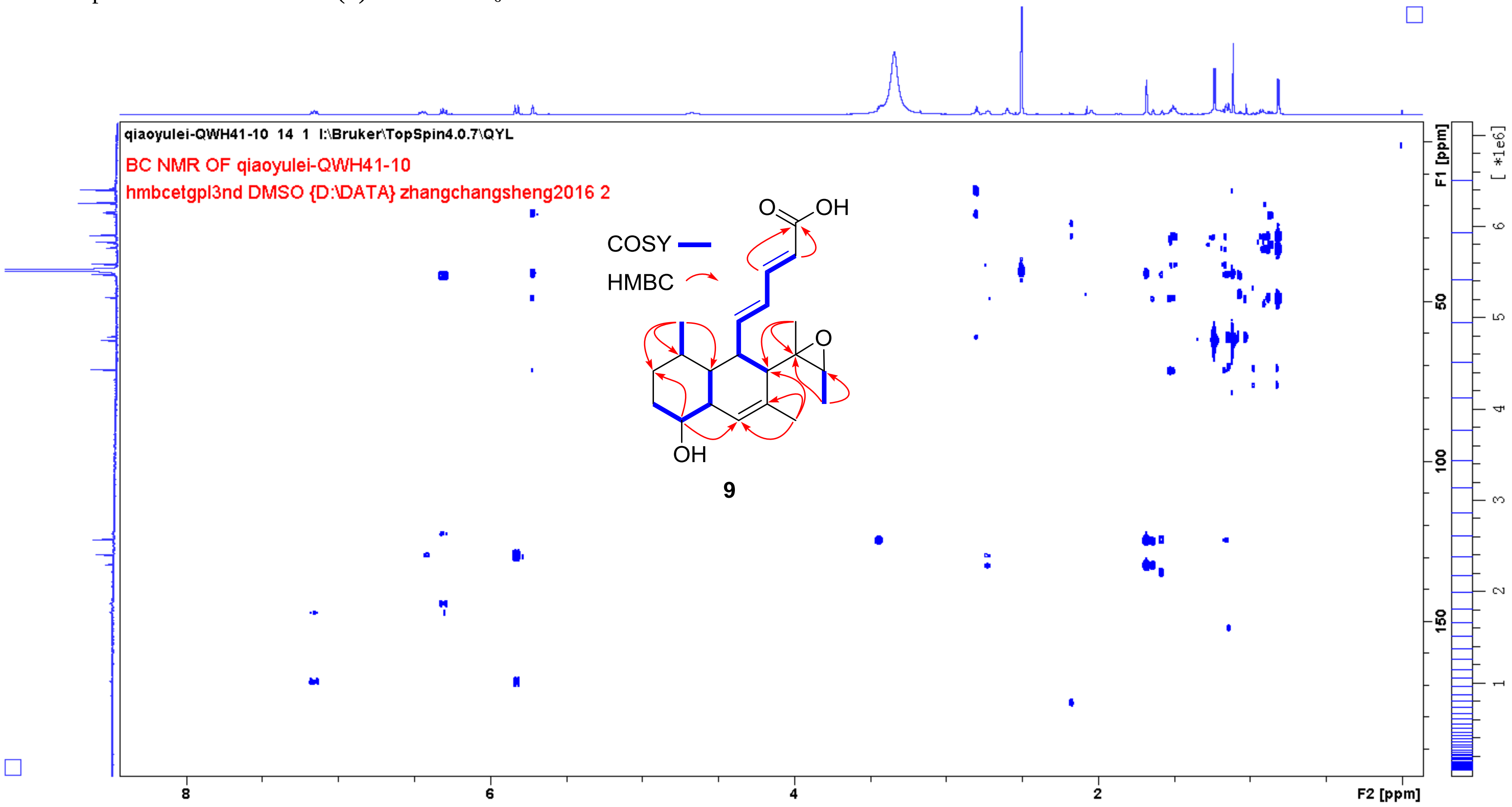

Figure S9. Spectral data for MK8383 G (**9**) (continued).

(I) The HMBC spectrum of MK8383 G (**9**) in DMSO-d_6.

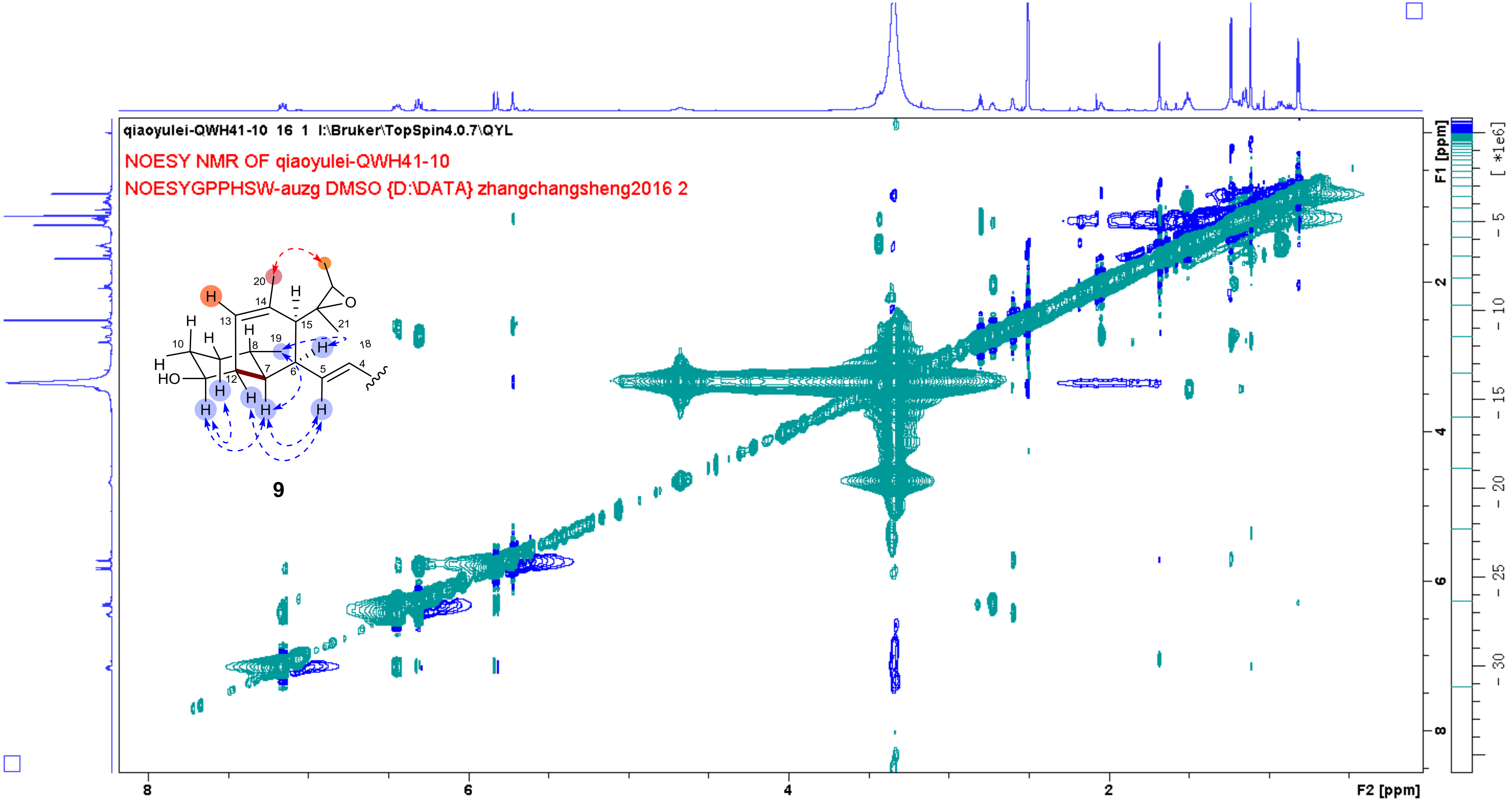

Figure S10. Spectral data for MK8383 I (**10**).

(A) HRESIMS

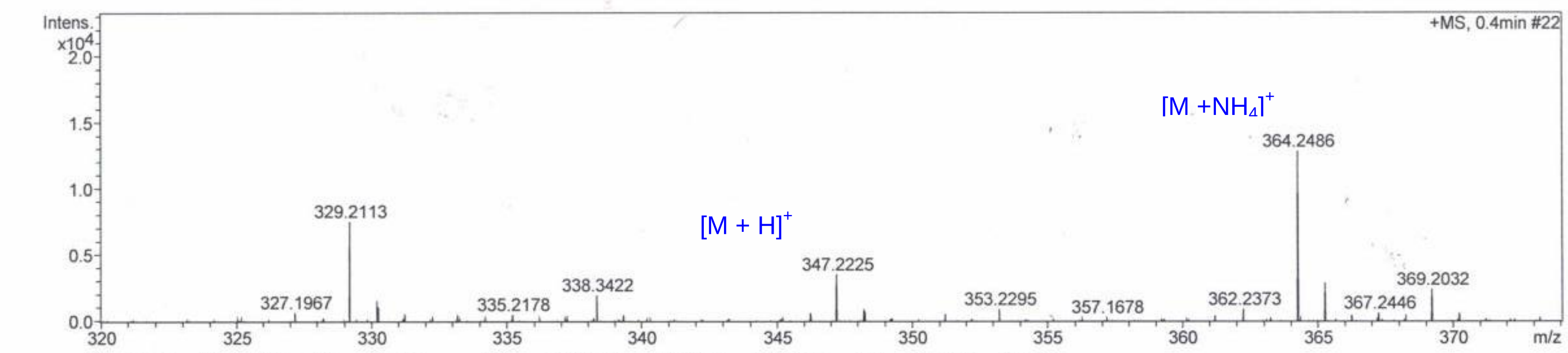

Meas. m/z	#	Ion Formula	Score	m/z	err [ppm]	err [mDa]	mSigma	rdb	e⁻ Conf	N-Rule
347.2225	1	C21H31O4	100.00	347.2217	-2.3	-0.8	19.2	6.5	even	ok
364.2486	1	C21H34NO4	100.00	364.2482	-1.1	-0.4	5.0	5.5	even	ok

(**B**) UV

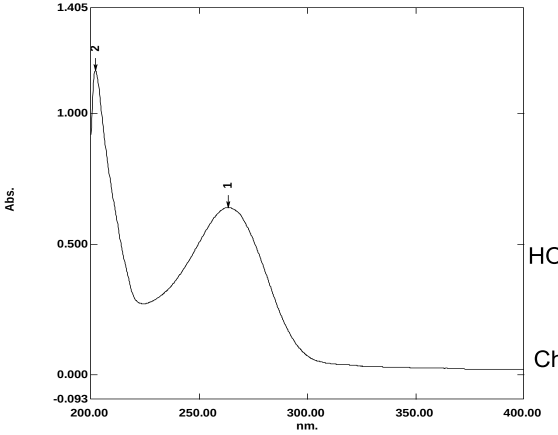

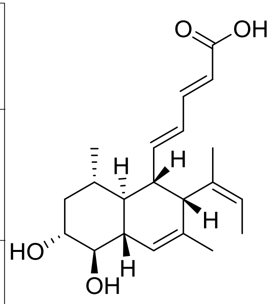

10

Chemical Formula: $C_{21}H_{30}O_4$

Exact Mass: 346.2

(**C**) IR

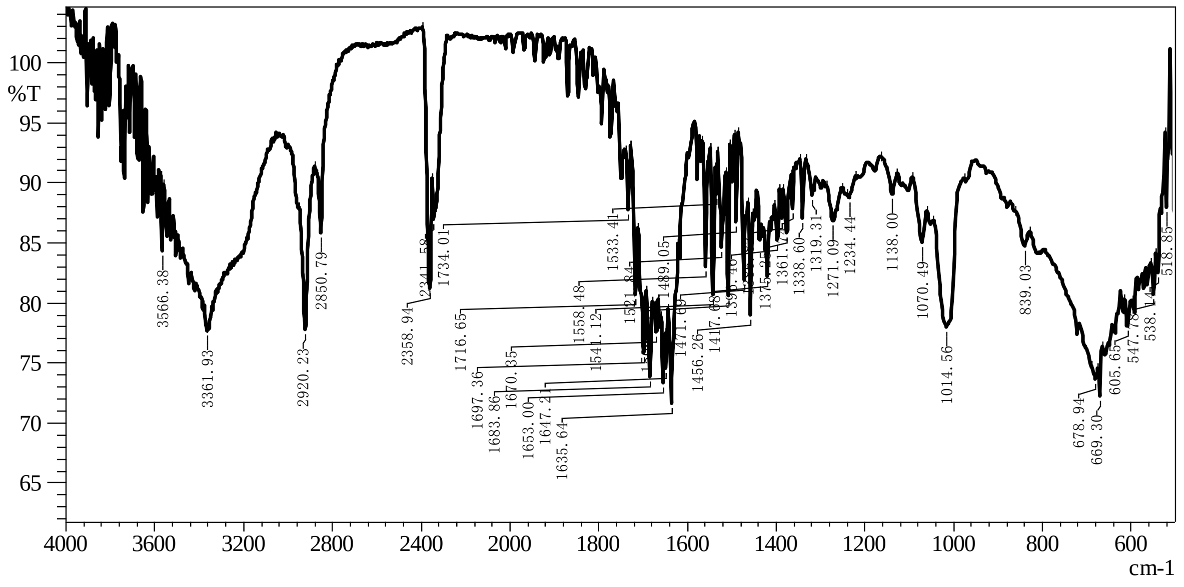

Figure S10. Spectral data for MK8383 I (**10**) (continued).
(D) The ^{1}H and enlarged ^{1}H NMR spectrum of MK8383 I (**10**) in Acetone-d_6.

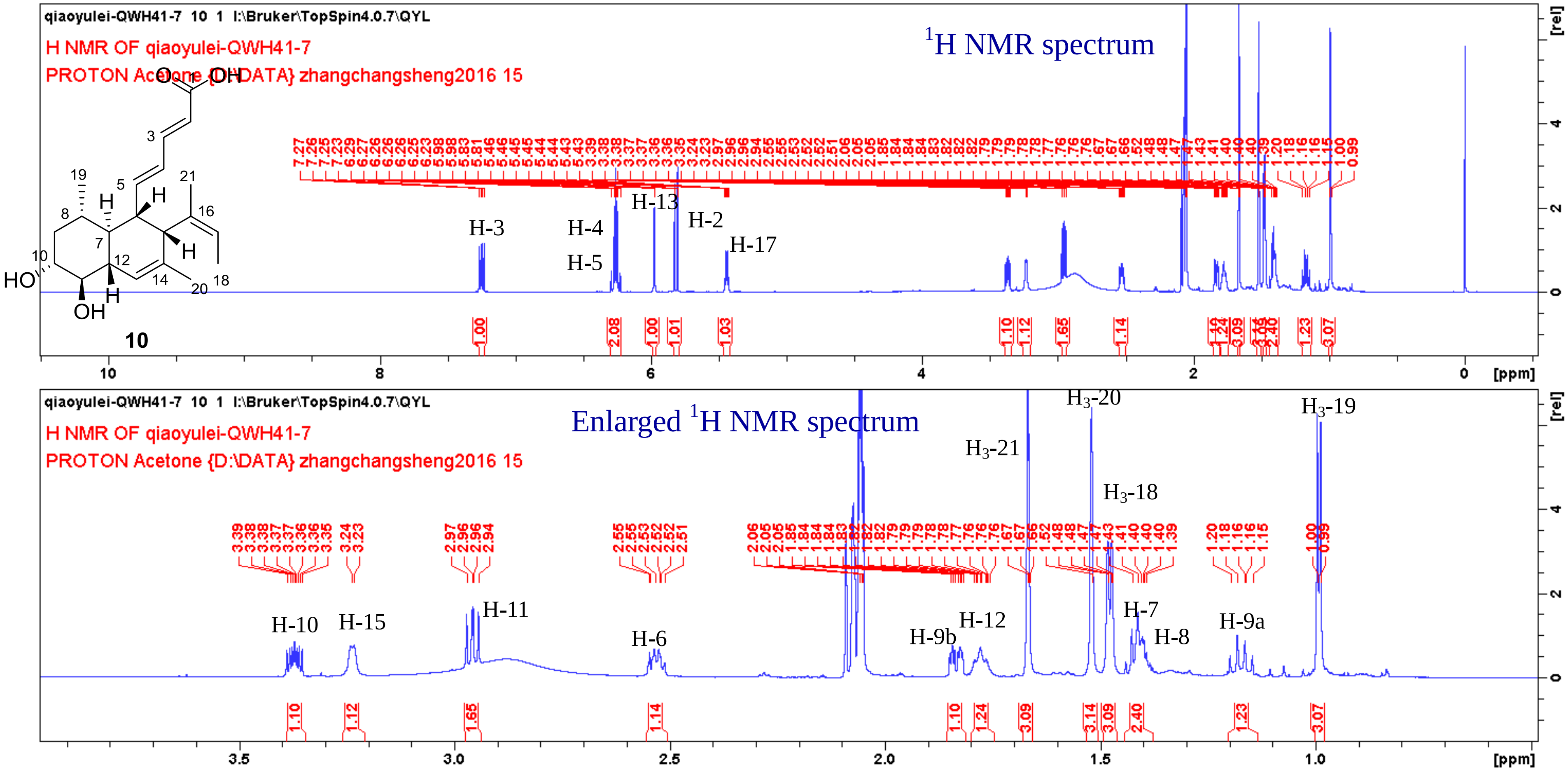

Figure S10. Spectral data for MK8383 I (**10**) (continued).
(E) The ^{13}C and enlarged ^{13}C NMRspectra of MK8383 I (**10**) in Acetone-d_6.

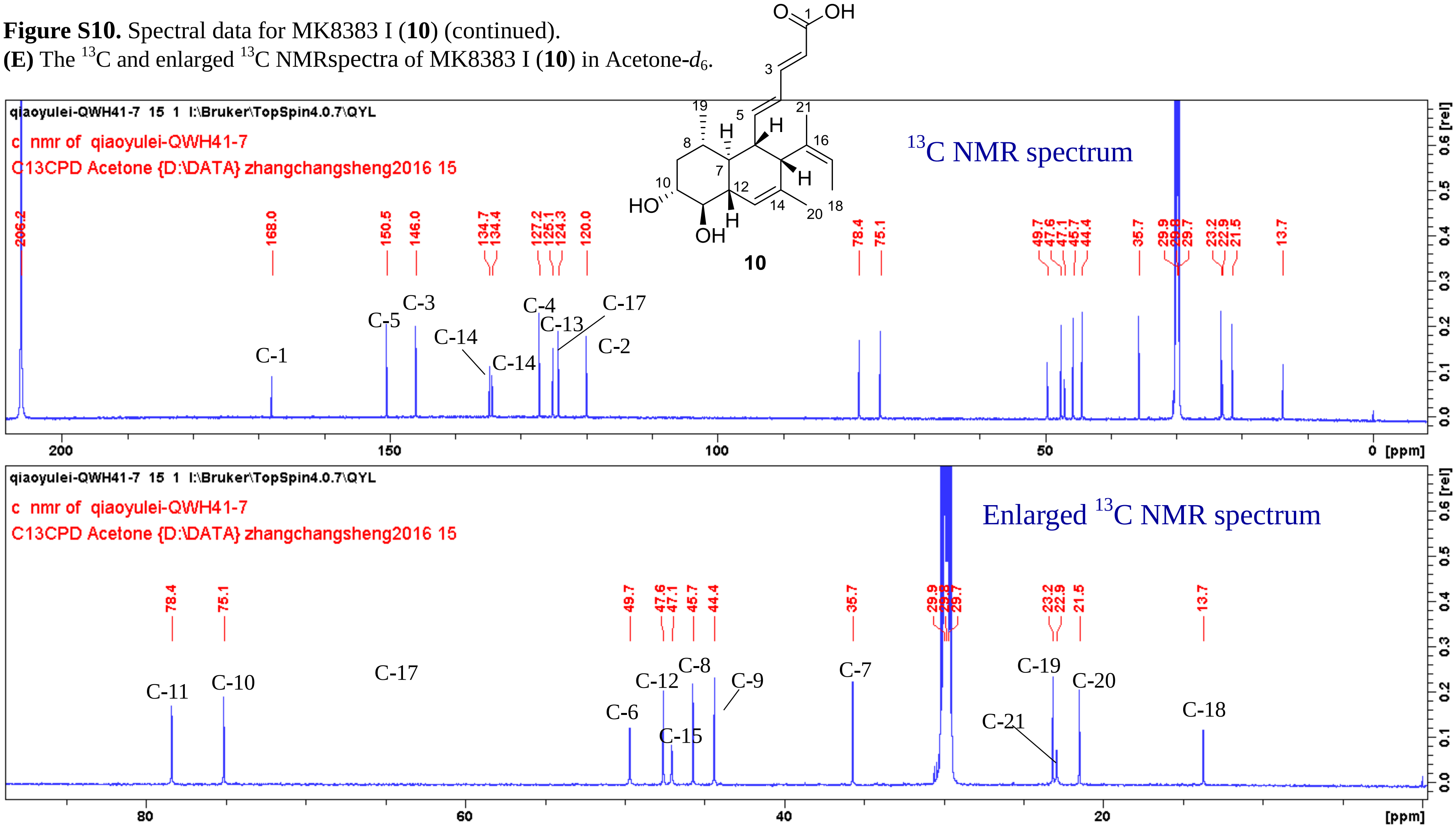

Figure S10. Spectral data for MK8383 I (**10**) (continued).
(F) The HSQC spectrum of MK8383 I (**10**) in Acetone-d_6.

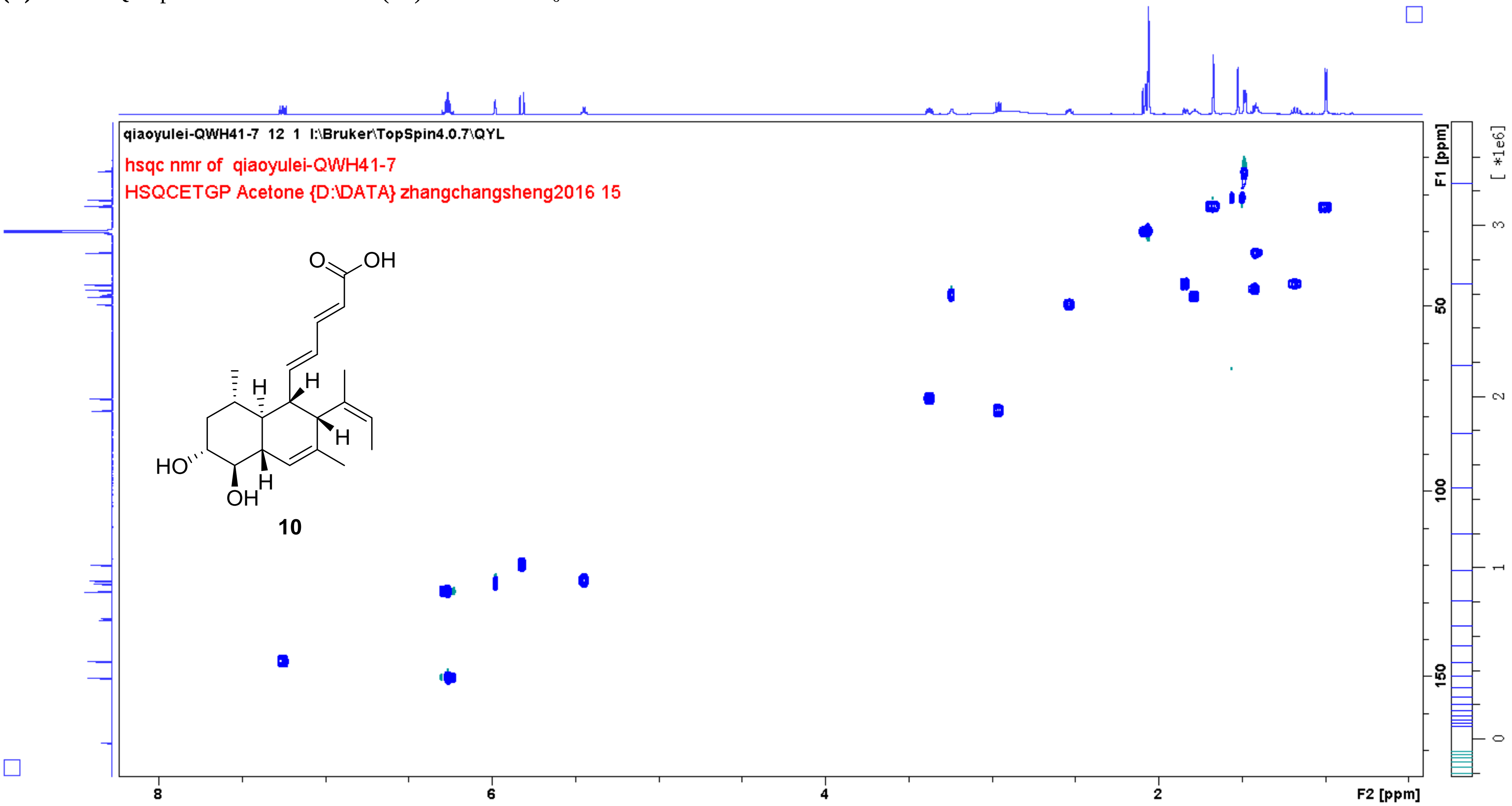

Figure S10. Spectral data for MK8383 I (**10**) (continued).

(G) The ^{1}H-^{1}H COSY spectrum of MK8383 I (**10**) in Acetone-d_6.

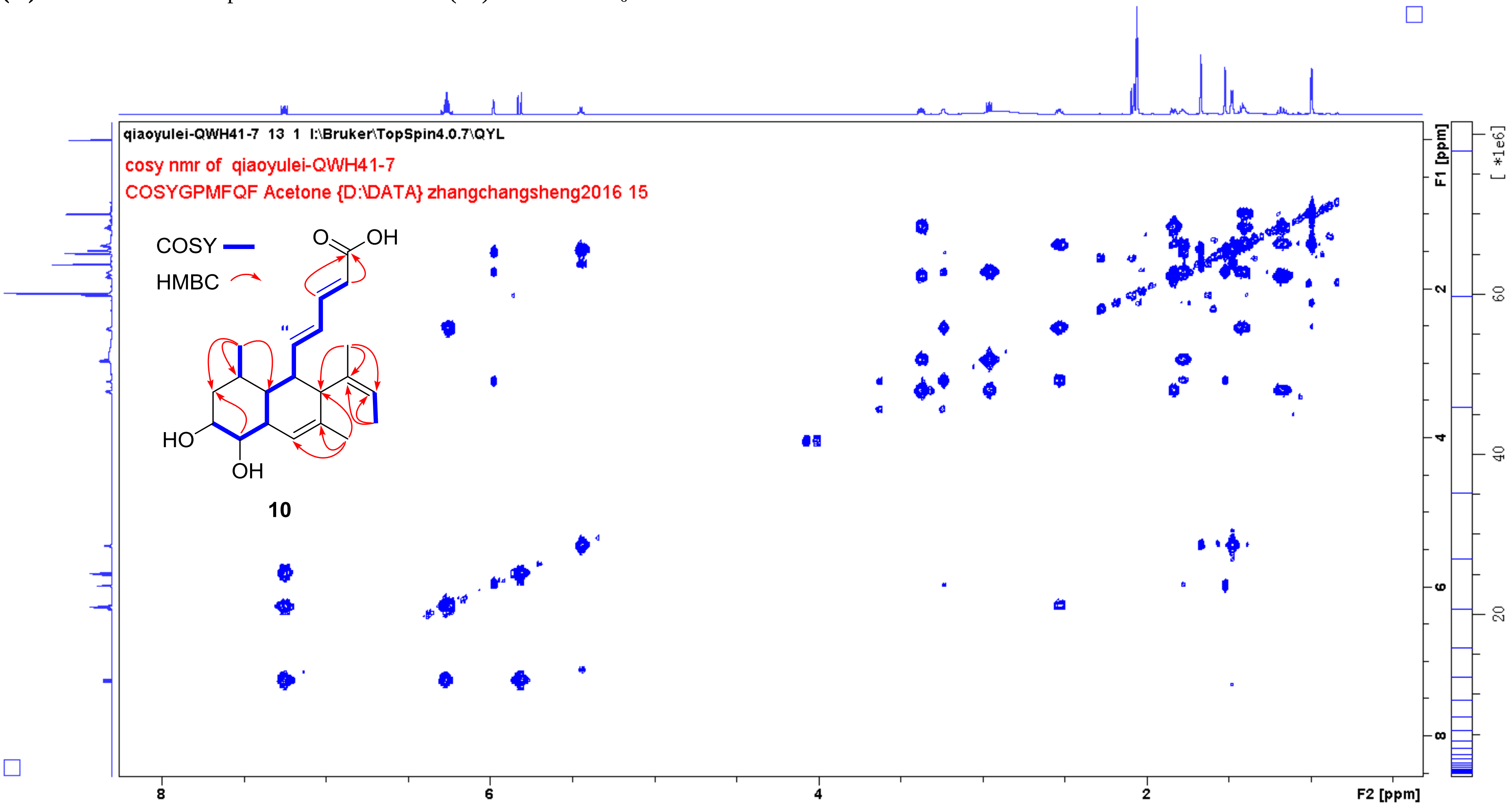

Figure S10. Spectral data for MK8383 I (**10**) (continued).
(H) The HMBC spectrum of MK8383 I (**10**) in Acetone-d_6.

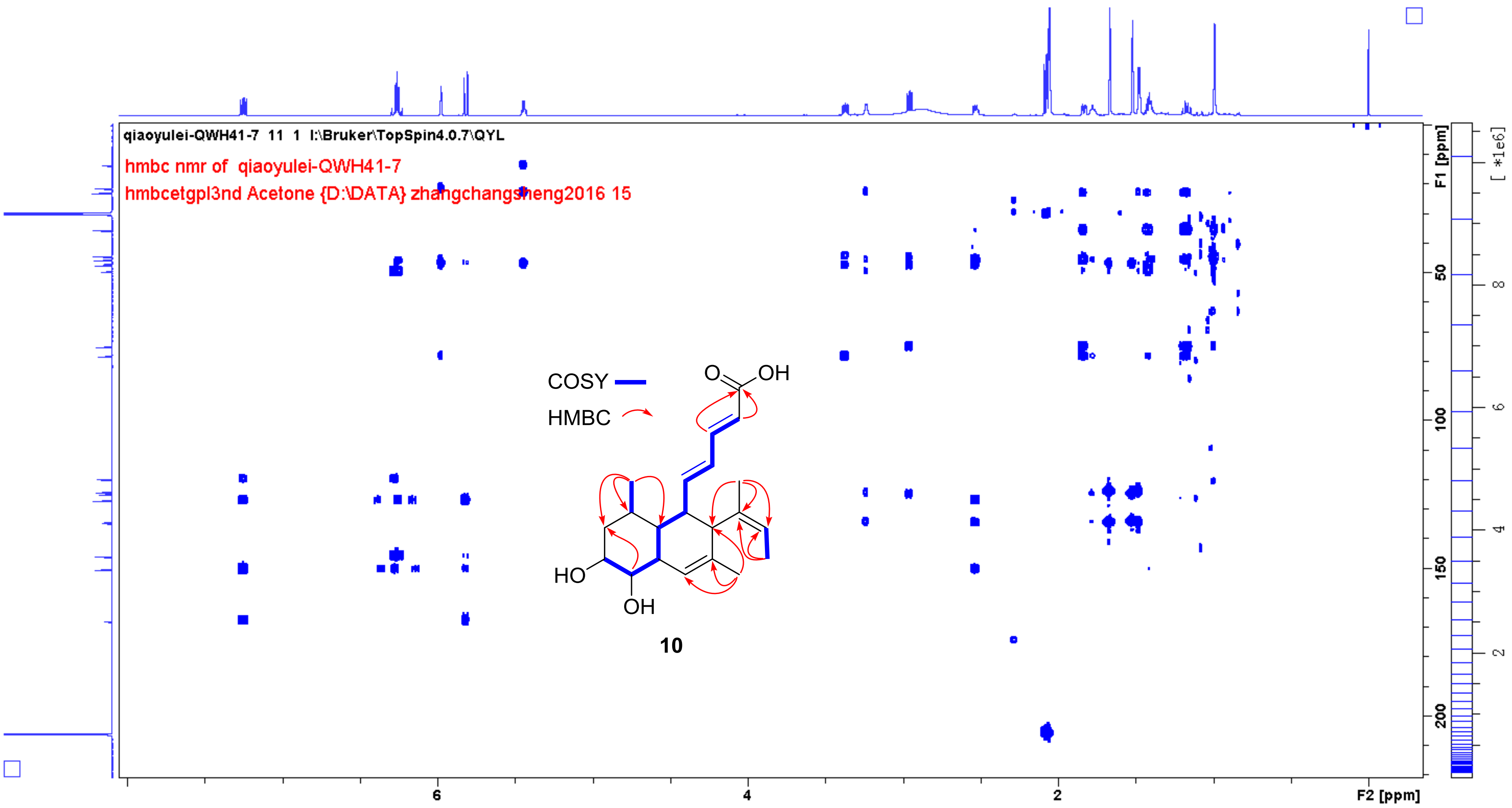

Figure S10. Spectral data for MK8383 I (**10**) (continued).
(I) The HMBC spectrum of MK8383 I (**10**) in Acetone-d_6.

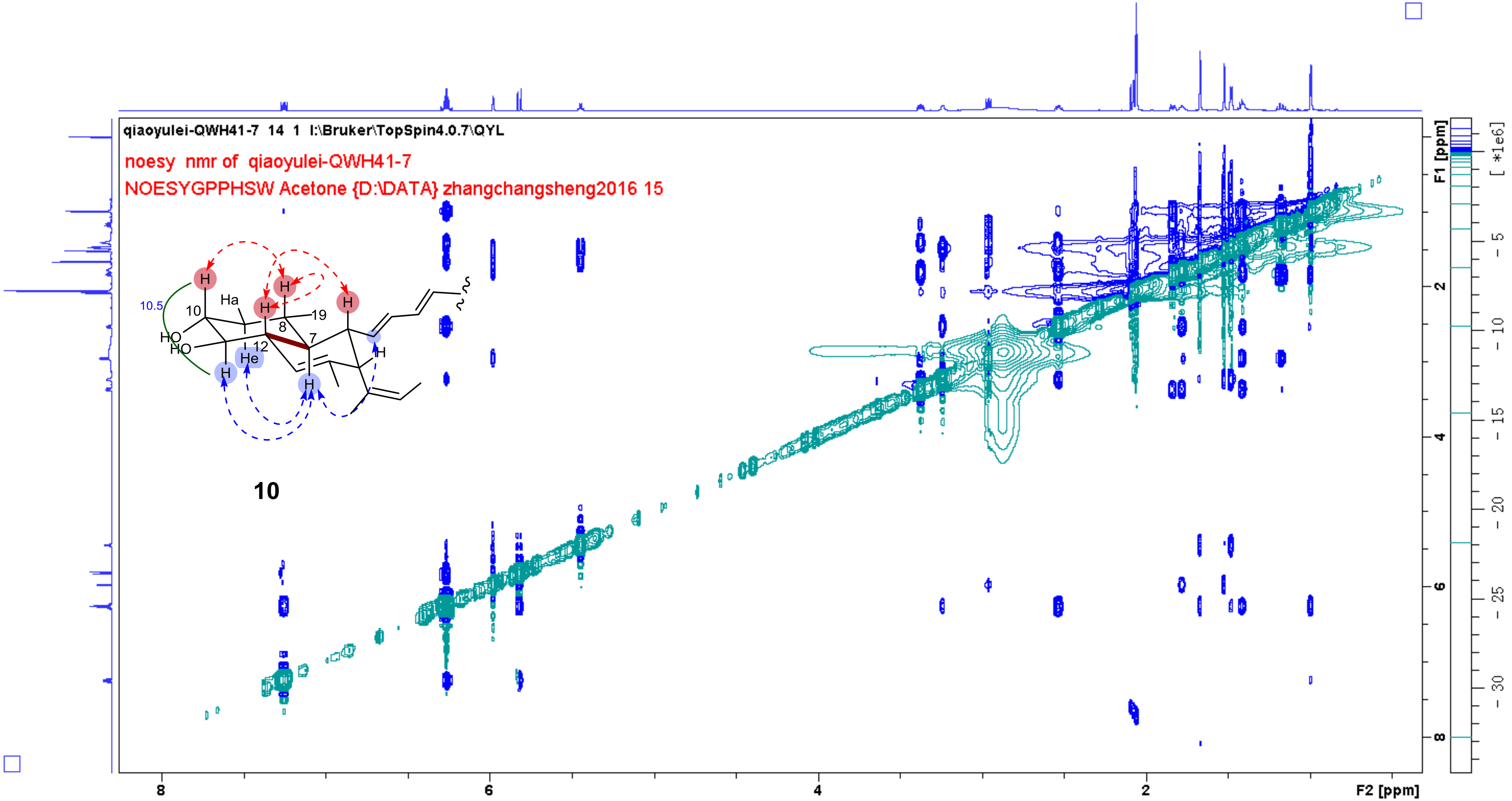

Figure S11. Antifungal activities of compounds **1**, **2**, **4**, **6**, and **10.**

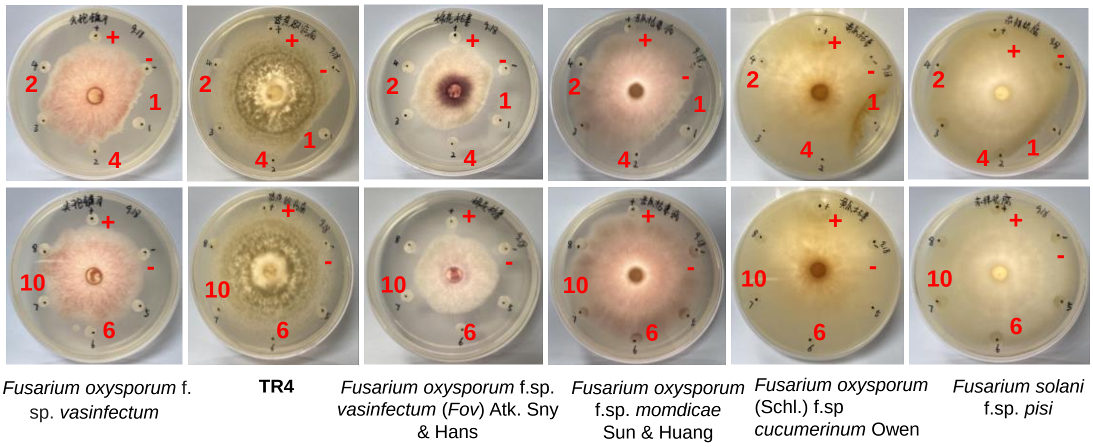

Antifungal activity of compounds **1**, **2**, **4**, **6**, and **10** against *Fusarium oxysporum* f. sp. *Vasinfectum*; *Fusarium oxysporum* f. sp. *cubense* race 4； *Fusarium oxysporum* f. sp. *Vasinfectum* (*Fov*) ATK. Sny & Hans; *Fusarium oxysporum* f.sp. *momdicae* Sun & Huang; *Fusarium. oxysporum* f. sp. *cucumerinu* Owen; *Fusarium solani* f.sp. *pisi* (+: nystatin; -: DMSO; 1, 2, 4, 6, 10 compounds **1**, **2**, **4**, **6**, **10**; no highlight: other compounds).
